# A Conserved Structural Signature of the Homeobox Coding DNA in HOX genes

**DOI:** 10.1038/srep35415

**Published:** 2016-10-14

**Authors:** Bernard Fongang, Fanping Kong, Surendra Negi, Werner Braun, Andrzej Kudlicki

**Affiliations:** 1 Department of Biochemistry and Molecular Biology, University of Texas Medical Branch, Galveston, TX, USA; 2 Institute for Translational Sciences, University of Texas Medical Branch, Galveston, TX, USA; 3 Sealy Center for Molecular Medicine, University of Texas Medical Branch, Galveston, TX, USA

## Abstract

The homeobox encodes a DNA-binding domain found in transcription factors regulating key developmental processes. The most notable examples of homeobox containing genes are the Hox genes, arranged on chromosomes in the same order as their expression domains along the body axis. The mechanisms responsible for the synchronous regulation of Hox genes and the molecular function of their colinearity remain unknown. Here we report the discovery of a conserved structural signature of the 180-base pair DNA fragment comprising the homeobox. We demonstrate that the homeobox DNA has a characteristic 3-base-pair periodicity in the hydroxyl radical cleavage pattern. This periodic pattern is significant in most of the 39 mammalian Hox genes and in other homeobox-containing transcription factors. The signature is present in segmented bilaterian animals as evolutionarily distant as humans and flies. It remains conserved despite the fact that it would be disrupted by synonymous mutations, which raises the possibility of evolutionary selective pressure acting on the structure of the coding DNA. The homeobox coding DNA may therefore have a secondary function, possibly as a regulatory element. The existence of such element may have important consequences for understanding how these genes are regulated.

Hox genes encode a group of transcription factors, responsible for developmental processes and establishment of the body plan^1^,^2^,^3^,^4^. All Hox genes and many other developmental transcription factors contain the homeobox, a DNA sequence encoding the functional DNA-binding domain. Hox genes are known for their colinearity: conserved arrangement on chromosomes that is the same as their order of activation along the body axis. The regulation is very precise, for example, the regions of activity of Hox genes are tightly confined to specific rhombomeres^5^,^6^,^7^,^8^,^9^ or to segments of the vertebrate anteroposterior body axis^10^. The vertebrate Hox genes are synchronized: the expression domains of paralogs from the A, B, C and D clusters are virtually identical^11^,^12^,^13^.

Despite 35 years of active research, the mechanisms of Hox gene regulation have remained elusive. Hox genes tend to be inhibited by more posterior ones, but this process appears not to be universal outside of vertebrates and is likely secondary to the yet unknown original mechanism of regulation^14^,^15^. It has been argued that chromatin structure^16^,^17^ and histone demethylation^18^,^19^,^20^,^21^ play important roles in activation of Hox genes, but the mechanism precisely directing chromatin modifications to specific loci at the right time remains mysterious. Ultraconserved regions and regulatory elements have been found within the coding sequences of Hox genes^22^,^23^, but the key questions remain unanswered. It is unknown what mechanism could be responsible for the exceptional synchronous colinearity of Hox gene clusters and the conserved synteny of other pairs of groups of homeobox-containing genes, however the topology of chromatin has been proposed to play a role in regulation of these genes^24^. Chromatin topology may depend on CTCF binding sites or long non-coding RNAs, however neither was confirmed to play a primary role in regulation of chromatin in the Hox clusters^25^,^26^. It is therefore possible that discovering a new DNA element will lead towards deciphering the regulation of genes within the Hox clusters.

Here, we report the discovery of a conserved feature of the DNA coding for Hox genes and certain other developmental transcription factors. While the function of the feature and associated mechanisms remain unknown and no conclusive statements concerning their specific role can be made without targeted genetic studies, statistical arguments point to significance of the motif and to its possible association with developmental processes and with regulation of chromatin structure.

Given the coincidence between presence of the homeobox domain and the unique evolutionary and regulatory properties of a gene, it is reasonable^27^,^28^ to hypothesize that the homeobox may be directly involved in regulation of Hox genes, or more specifically that the homeobox DNA sequence itself plays a role in regulating the gene it is contained in. While the DNA sequence of the homeobox is not ultraconserved^22^,^29^,^30^, a direct link may exist between the coding sequence of the homeobox and certain structural properties of DNA in this region. The local structure of chromatin is known to depend on the GC content, which can also mark transcriptionally active regions^31^, and on other quantitative characteristics, e.g. the Hydroxyl Radical Cleavage (HRC). In this work, we show that the HRC pattern in the homeobox coding DNA displays a significant structural property that is conserved not only between Hox genes within a species but also between distant species. The mechanistic role of the structural feature remains unknown, however one possible explanation of its conservation is its putative function in regulation of the Hox genes. The possibility of regulatory elements embedded in coding sequences has been explored among others by ref. 32, who identified thousands of possible examples of such sites and argued that dual encoding of amino acid and regulatory information may be a fundamental feature of genome evolution. A possible role in transcriptional regulation may be either as a direct transcription factor binding site or indirect, as a locus important for directing epigenetic modifications or affecting chromosomal conformation. Other explanations of the phenomenon may include a role in subsequent transcriptional or post-transcriptional processes.

## Results

### The GC content of the homeobox in Hox genes

We calculated the average GC content within mouse and human homeobox-containing genes, as well as eight Hox genes of the fruit fly *D. melanogaster* (Table 1). In each of the three species, the homeobox-coding DNA is flanked by regions of significantly higher GC content. This observation raises the possibility that the properties of DNA or chromatin within the homeobox coding sequence may serve a conserved biological function. While the hypothetical function is unknown, it is possible that it will be related to recognition of the Hox genes by a regulatory process.

### The Hydroxyl Radical Cleavage motif (HRC3)

To investigate the structure of the homeobox DNA with more detail, we analysed the predicted HRC pattern of genes containing these sequences. The HRC is an important parameter correlated with the local structure of chromatin, corresponding to the width of the minor groove of DNA^33^. HRC provides information on the local shape and structure of the DNA helix and has been shown to correlate with functional non-coding regions in the genome^34^. The HRC can be reliably estimated from the sequence of DNA^35^.

**Figure 1:**
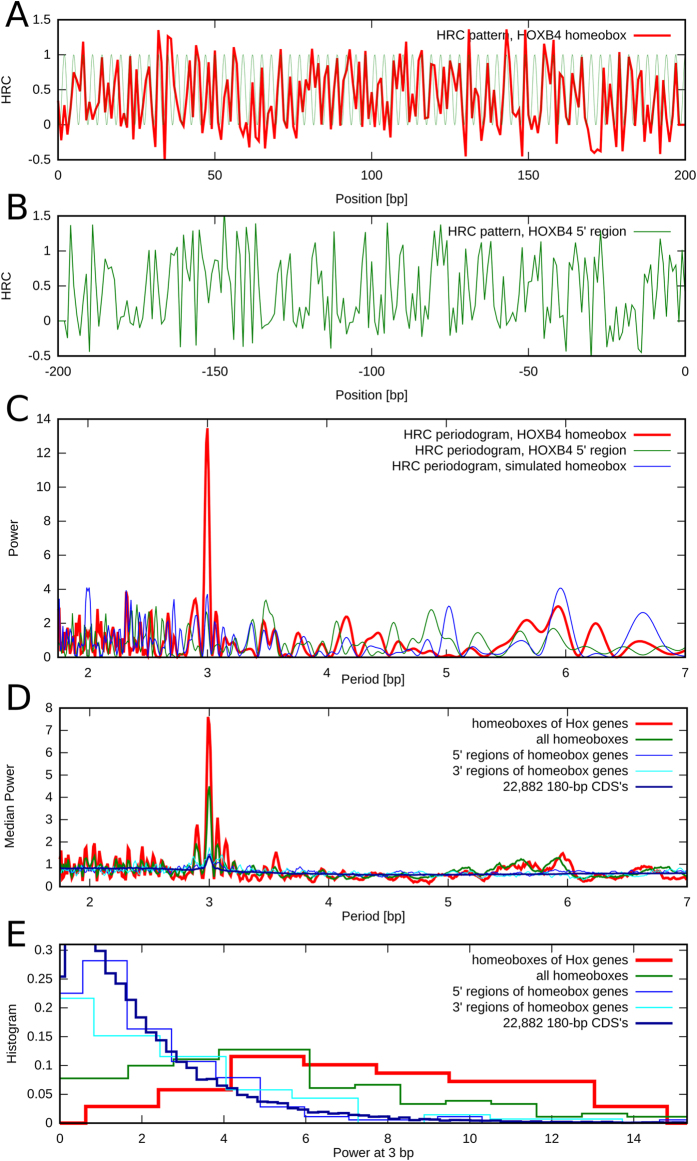
The periodicity of hydroxyl cleavage pattern within the homeobox. (**A**) The pattern of HRC in the mouse HoxB4 homeobox coding sequence (red) shows a period of three base pairs (dotted line represents a harmonic oscillation with a 3 bp period; note that the two plots are consistently in phase with one another). (**B**) The periodic signature is absent in other regions of the gene. (**C**) The periodograms of the homeobox HRC (red), the HRC of a coding region adjacent to the homeobox (green) and the HRC of a simulated DNA sequence coding for the same protein sequence of the homeodomain but using different codons (blue). The highly significant peak is present only in the actual homeobox. (**D**) The HRC3 patterns in homeobox DNA are more prevalent than in other coding sequences. The median periodogram of HRC of the homeoboxes of mouse Hox genes (red), all homeobox genes (green), outside of homeobox in homeotic genes (blue: 180 bp adjacent towards 5′ end, teal: between homeobox and the 3′ end), and randomly chosen coding sequences (dark blue). (**E**) Histograms of periodicity score at T = 3 base pairs.

The HRC pattern of the homeobox region of the mouse gene HoxB4 is presented as an example in the top panel of Fig. 1. A striking feature of the homeobox is the 3-base pair periodicity (the “HRC3” signature), which is absent outside of the homeobox. For comparison, the predicted HRC pattern of the coding sequence adjacent to the homeobox toward the 5′ end of the gene is shown in panel B of Fig. 1. We quantified the significance of the periodic pattern by computing the periodogram^36^,^37^,^38^ of the data, defined as an estimate of the amplitude of the harmonic oscillation best fitting the signal within the interval [*a*,*b*], as a function of the period *T:*


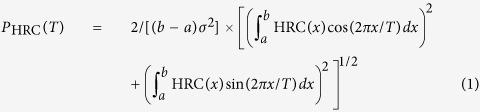


The significance *p* of *P*_*HRC*_ at a given period *T* reads: *p* = (1 − *P*/n)^n − 1 ^36,39 (if sample variance is used as σ in the calculation) and can be approximated as *p* = exp(−*P*)^40^,^41^. For the homeobox of HoxB4, the periodogram shows a very significant (p < 1.e-5) peak corresponding to a period of three base pairs in the homeobox (red graph in Fig. 1C). The periodogram of the HRC in the 180-bp region adjacent to the homeobox in the coding sequence (green curve in Fig. 1C) does not have a significant peak at 3 bp. The periodicity analysis of all mouse Hox genes reveals the same HRC3 feature significant in 37 out of 39 homeobox-coding sequences of mouse Hox genes, with the exception of two genes: HoxA13 (p = 0.219) and HoxD8 (p = 0.104), see Table 2. For32 mouse Hox genes the significance is below 0.01, and for 21 genes p < 0.001. Examples of Hox genes in which the periodicity is significant but less prominent are HoxC13, HoxC8, HoxD13, close homologs of HoxA13 and HoxD8 (see also Supplementary Figure File SF1). Note that ortholog groups 8 and 13 are arguably the fastest evolving Hox genes, and are expected to have diverged the most from the postulated original *Urhox* gene^14^. In general, the HRC3 signature is significantly stronger in anterior Hox genes (Hox 1–7) compared to posterior ones (p = 0.0039 in mouse; p = 0.0048 in human; t-test).

The results point to selective pressure favouring the three-base pair periodicity of HRC within the homeobox. There are two possible explanations for the observed phenomenon: it can be either a consequence of a conserved amino acid sequence or the selection of codons within the homeobox. In the former case, the HRC3 pattern would be associated with any DNA sequence coding for the protein sequence of the homeodomain. Conversely, if the pressure is indeed on the properties of the chromatin, the pattern would be weakened or disrupted by synonymous mutations. To verify that codon selection does significantly contribute to the HRC pattern in homeoboxes, we simulated 1,000 DNA sequences with synonymous mutations for each amino acid, and we predicted the HRC pattern for the simulated sequences. The simulated DNA sequences were generated so that they would be coding for the same protein sequence, and use codon frequencies that are typical for all mouse CDS (coding DNA sequences); such comparison may show that evolutionary pressure on codon selection exist within the homeoboxes. We observed that in the simulated DNA sequences coding for the same homeodomain amino acid sequence, the periodicity of the HRC3 pattern is greatly disrupted. The result confirms that generally a strong bias exists towards using codons that maximize the HRC3 signal in the homeoboxes of mouse Hox genes. Specifically, for 36 out of 39 Hox genes, the fraction of random sequences that would have produced stronger HRC3 signal than the one actually observed in the homeobox of the gene is much lower than the expected value of 0.5. The average fraction is 0.134, and the median is 0.030. For genes with strong HRC3 signal (P_HRC3_ > 6), the average and median are 0.031 and 0.008, respectively. This result suggests that the codon selection effect is especially strong in the genes with highly significant HRC3 feature, although causation cannot be determined based on the presented data.

The simulation results are presented in Supplementary Table S1, and in Supplementary Figure S1 that also shows the relation between the HRC3 amplitude and the codon selection effect. Supplementary Table S2 shows details of codon usage within the homeoboxes of Hox/Antp-Ubx clusters in mouse, human and fly. Codon bias specific to homeoboxes exists in all three species, moreover the homeobox to whole-exome codon usage ratios are virtually identical between mouse and human (Pearson Correlation Coefficient, PCC = 0.97) and also similar in *Drosophila* (PCC = 0.38, p = 0.018), see Supplementary Figure S2; in most cases GC-rich codons are favoured in the homeoboxes.

### The HRC3 motif in mouse homeobox-containing genes outside of the Hox clusters

Analysis of additional 119 mouse homeobox-containing genes outside of the Hox clusters reveals the presence of the same periodic signature in a large fraction of these genes (see Fig. 1D,E, Supplementary Figure File SF2, and Supplementary Table S3). Homeobox genes in animals are categorized into several classes based on evolutionary relationships and additional domains^42^. Here, we compared the average amplitudes of the HRC3 pattern for homeoboxes of genes from the ANTP, LIM, POU, ZF, PRD and TALE categories. Intriguingly, while every class contains genes with a significant HRC3 signature in the homeobox, the pattern is most prevalent in the ANTP class, which includes the NK, Hox, and ParaHox groups of genes. The average P_HRC3_ for mouse ANTP genes is 6.62, while for the other classes it is much lower (2.80, 3.17, 2.65, 2.88 and 3.43 respectively for the LIM, POU, ZF, PRD and TALE categories). The systematic difference between genes in different classes suggests that the HRC3 signature may be functional in homeobox-containing genes that are at least partially organized in clusters with conserved synteny or in conserved pairs (as NKX2.1—NKX2.8 or NKX2.2—NKX2.4).

If the HRC3 feature in homeobox genes is indeed related to the clustering of those genes on chromosomes, one may expect a general correlation between the HRC3 signature of a gene and presence of other homeobox genes in its chromosomal neighborhood. The relation between distance to the nearest homeobox-containing gene and the amplitude of the HRC3 signal is shown as scatterplot in Supplementary Figure S3. Indeed, the mouse genes that have other homeobox genes in their vicinity (within 300kbp) tend to display stronger HRC3 signatures. The dependence is statistically significant, with a p-value of 2.16e-7 (Wilcoxon rank sum test), or 5.14e-8 (t-test). Intriguingly, the difference between isolated and clustered homeobox-containing genes remains significant even after removing all Hox genes from the analysis (p = 0.017, Wilcoxon; p = 0.012, t-test).

While the HRC3 pattern is present in most homeoboxes, it is not common outside of the homeobox coding DNA. The difference between homeoboxes and other coding sequences is evidenced by the periodograms of the HRC patterns calculated for 22,882 randomly chosen 180-bp coding sequences from the mouse genome. The results are summarized in Fig. 1D,E, respectively showing the median periodograms and the histograms of the most significant periods of HRC in: the homeobox of mouse Hox genes, the homeobox of all homeobox-containing genes, in the 5′ and 3′ adjacent regions and in randomly selected coding sequences. Note that the 3 bp period is exceptional-no other significant periodicities (in the range between 2 bp and 7 bp) are common either to the Hox genes or to other coding sequences. The HRC3 signal in randomly chosen coding sequences is very significantly smaller than in all homeoboxes, and than in the ANTP class genes (p-value < 10^−11^ in both cases). On the other hand, the histogram of P_HRC3_ in the coding regions of homeobox-containing genes outside of homeobox is not remarkably different from that of randomly chosen coding sequences.

**Figure 2:**
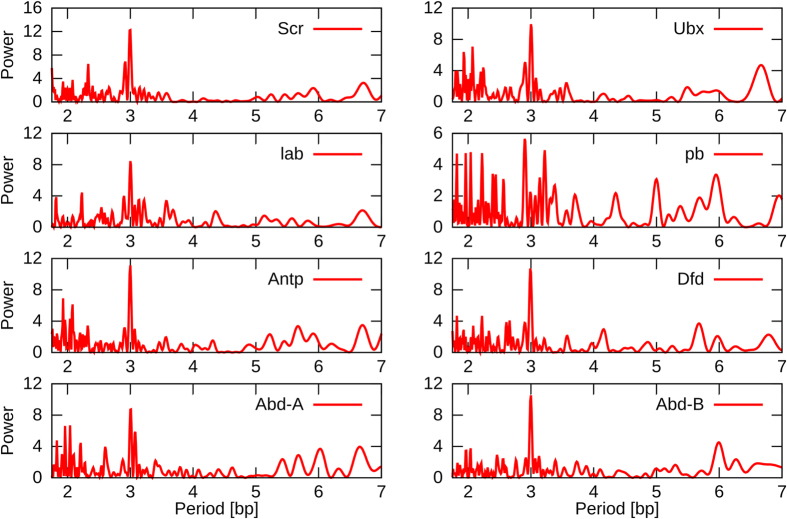
Periodograms of HRC in homeoboxes of fly Hox genes. The same pattern as in mammalian genes is present, revealing evolutionary conservation of the HRC3 periodic structural signature.

The HRC3 patterns of human homeoboxes are virtually identical to the patterns observed in mouse, and so are the periodograms at 3 bp (see Table 2, Table S4, and Supplementary Figure Files SF1 and SF2). To test whether the periodicity in the pattern is conserved even beyond vertebrate animals, we analyzed the HRC in the homeoboxes of the long-germ insect *D. melanogaster*. The 3 bp period is highly significant in seven out of the eight ANTP/Hox genes (Fig. 2, Supplementary Table S4). It is weaker in the Proboscipedia gene, whose HRC pattern is similar to the homologous mouse genes HoxA2 and HoxB2 (compare Fig. 2 and Supplementary Figure SF1), which may suggest the presence of a functional variant of the structure of the homeobox DNA.

### Evolutionary conservation of the HRC3 signature

The results presented for human, mouse and Drosophila suggest that the pattern may be conserved in the Hox genes also in other animal species. To test this hypothesis, we analysed the HRC3 pattern of homeoboxes in Hox gene homologs, as defined by *GENBANK,* of several additional metazoan species. In each organism, we identified the Hox genes and in each gene we computed the amplitude and significance of the HRC3 signature of the 180-bp sequence aligned with the homeobox. The results are presented in Table 3 and Supplementary Table S4. The data suggest that the pattern is generally conserved in vertebrate and invertebrate species with true segmentation of their bodies, while it may be less significant in non-segmented animals, as mollusca or tunicates.

### Functions of other genes with the HRC3 signature

**Figure 3:**
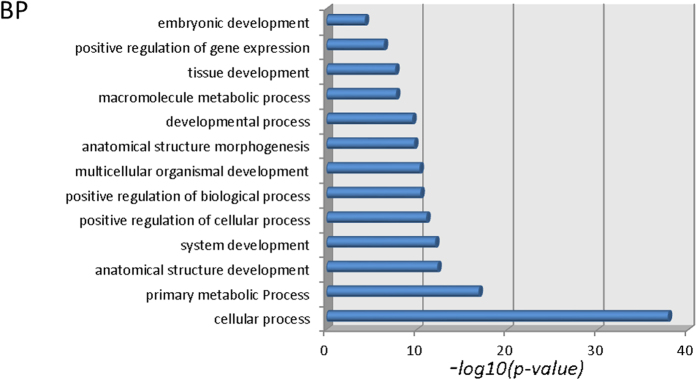
The GO biological processes significantly enriched in mouse genes containing the HRC3 signature (P_HRC3_ ≥ 10). Note the high prevalence of processes associated with development.

If the HRC3 signature is indeed recognized by an unknown molecular mechanism related to the function of Hox genes and certain other homeobox-containing genes, it is possible that such mechanism may also be employed by other genes and processes. To address this question, we searched for the HRC3 signature in the sequences of all mouse coding genes. Since this search was not restricted specifically to the 180-bp homeobox sequence but rather included the entire transcribed gene, this analysis required a more strict threshold on significance of detection to avoid a large number of false positives. We performed functional annotation enrichment analysis for mouse genes with a threshold of P_HRC3_ = 6 (p < 0.00248) and P_HRC3_ = 10 (p < 4.54e-5), using the DAVID (Database for Annotation, Visualization and Integrated Discovery, http://david.abcc.ncifcrf.gov/) web server. We uploaded the gene list of genes containing HRC3 signature at P_HRC3_ ≥ 6 (8000 genes) and P_HRC3_ ≥ 10 (5692 genes) to the server and set the background as the total number of genes present in the mouse genome (version GRCm38.p1) annotation. Moreover, only GO terms containing at least 200 of the input genes and a q-value (Benjamini corrected p-value) < 0.05 were selected. The Biological Processes most significantly enriched among those genes are summarized in Fig. 3; the complete results are presented in Supplementary Table S5 and in Supplementary Figure S4 depicting the top enriched Molecular Functions and Cellular Components. The analysis shows a highly significant enrichment of processes related to development (GO:0009888, GO:0048731, GO:00325020) as well as regulation of gene expression and metabolic processes (GO:0010628, GO:0031325, GO:0044260). One of the molecular functions significantly enriched in genes containing the HRC3 signature is DNA binding (GO:0003677). This observation led us to testing whether the HRC3 motif may significantly overlap with sequences coding for binding domains other than homeobox. To this end, we searched for HRC3 signatures overlapping with other DNA binding domains, as defined by *InterPro*, the database of protein families, domains and functional sites^43^. The overlap enrichments (relative to sequences coding for binding domains positioned randomly in the exome) are presented in Supplementary Table S6: while the interpretation is not straightforward due to different sizes of these domains and different levels of homology, the results may suggest that some domains (Forkhead, bHLH) also tend to overlap with the HRC3 signatures, while others (Ets, Pou) do not have a significant overlap (simulated overlaps equalled or exceeded the actual values). It is therefore possible that a yet unknown function of the HRC3 element exists that is not limited to the homeobox-containing genes but is also affecting certain other classes of transcription factors.

### Relation to nucleosome positions and ultraconserved regions

To test whether any putative function of the HRC3 signature may be related to the nucleosome occupancy, we predicted the nucleosome-rich sites in mouse Hox clusters^44^,^45^ and found that on average the nucleosome occupancy within the homeobox does not differ from other coding regions in the Hox genes (See Supplementary Figure S5). This suggests that even if the HRC3 feature is indeed functional, its mechanism is not likely to be directly related to the nucleosome occupancy along its sequence.

Intriguingly, in some of the genes (e.g. HOXA5, HOXB5, HOXC4) a second, shorter HRC3 region exists outside of the homeobox that may coincide with an ultraconserved region (UCR) identified by ref. 22 (see Supplementary Figure S6). This observation may suggest that while a HRC3 signature is required in Hox genes, in some cases it has moved outside of the homeobox, however it is based on a small sample of short periodic sequences and is therefore inconclusive.

### Functional correlations of genome-wide loci with the HRC3 signature

One possible function of sequences carrying the HRC3 signature could be recruiting transcription factors or other proteins to the specific chromosomal loci. To check if such function could be valid, we investigated the overlaps between the HRC loci and known binding sites inferred from ChIP-seq experiments, obtained by the ENCODE project^46^. Hox genes and other developmental transcription factors are regulated primarily during embryonic development. Since the data available from ENCODE are not collected in embryonic tissue, we analyzed the genome-wide distribution of HRC3 loci, without restricting it to developmental transcription factors. We have analyzed ENCODE data for 161 DNA binding proteins, to note that some binding sites are significantly enriched in the HRC3 motifs, while others are not (Table 4, Supplementary Table S7). The enrichments (estimated by comparing with simulated distributions of loci) range from less than 1 (significant depletion) to over 6-fold enrichment ratio. This observation is consistent with the HRC3 sites being involved in specific cellular or systemic functions, at the same time suggesting that HRC3 is *not* a general mark of a process (as chromatin accessibility) that would affect all TFs equally. Notably, the DNA binding proteins most significantly coinciding with HRC3 include proteins involved in epigenetic modifications of chromatin, such as SUZ12-chromatin silencing; KDM5B, KDM5A, PHF8–histone demethylases; EZH2, RBBP5–histone methyltransferases; SAP30, HDAC1, HDAC6–histone deacetylases; CHD1, SMARCB1-chromatin organization modifier and also CTCF–a transcriptional repressor and a key regulator of chromatin architecture; etc. While this result is not sufficient to draw conclusions concerning the role of HRC3, it is consistent with the possibility of the motif being important in regulation of chromatin modifications and control of the epigenetic state of the cell and in agreement with studies in *Drosophila* showing that histone modifications are responsible of defining the segmental regulatory domains^47^.

## Discussion

We analyzed the DNA sequences coding for the homeodomains of metazoan Hox genes using a new computational approach that combines sequence alignment, prediction of structural features and spectral analysis. We have discovered a three-base-pair periodic signature (“the HRC3 pattern”) in the hydroxyl radical cleavage profiles of the homeobox DNA. The hydroxyl radical cleavage profile correlates with local structural properties and bendability of the double-stranded DNA. The discovered phenomenon of characteristic periodicity of HRC (HRC3) is present in Hox genes of human, mouse, and fly and other segmented bilaterian animals. In human and mouse the signature is also found in other homeobox-containing genes, especially in genes that have other homeobox genes in their chromosomal neighborhoods. The conservation of the HRC3 pattern both between genes within a species and between distant species of metazoans raises the possibility that the structural feature arose early in the evolution, although it cannot be determined whether it was present in the postulated ancestral, Pre-Cambrian *Urhox* or *ProtoANTP* gene, from which all extant Hox genes are thought to have evolved^42^. The signature is also not universal as it is absent in homeoboxes of many mammalian genes that are not members of the ANTP class; it is also only marginally significant in the Hox gene orthologs in some non-segmented organisms, as the mollusk *Octopus vulgaris*^48^.

We have shown that even synonymous mutations will disrupt the HRC3 pattern. Its observed persistence, along with the different GC content in the homeoboxes, constitutes evidence that the pattern may play a role in the codon selection within these genes, and suggests that the remarkable conservation is due to evolutionary pressure on the structural properties of the homeobox coding DNA. Similar effect on codon usage has been reported for exonic binding sites of the CTCF and NRSF(REST) transcription factors^32^. While the biophysical nature of the HRC3 signature remains unknown, it is likely that the HRC3 pattern is characteristic of a DNA structure that serves as a regulatory element within the Hox clusters. Indirect evidence has been presented suggesting that some DNA-binding proteins may indeed be backbone conformation-specific, rather than DNA sequence-specific^35^,^49^, also periodic features have been recently indicated as functionally significant, e.g. in selection of transcription start site^50^. If the entire homeobox constitutes a regulatory element, it would thus play a dual role, both in regulating the targets of Hox genes and in regulating expression of the Hox genes themselves. This double function could make the homeobox a perfect material for a logical element that has evolved into the basic building block of the circuitry encoding and executing the complex logic of developmental programs. Our discovery may provide a key step towards understanding the molecular basis for the colinearity and synchronization of Hox genes, the conserved synteny of other homeobox-containing transcription factors, and its relation to the intricately regulated somite clock^51^,^52^,^53^,^54^,^55^. The observed significance of HRC3 signature is significantly higher in anterior Hox genes than in posterior ones; if the motif is indeed involved in regulation of the Hox genes, such difference may be explained by the stronger conservation of the anterior body plan than posterior across the animal kingdom.

Genome-wide analysis points to highly significant (up to over 6-fold) enrichment of HRC3 signatures among binding sites of proteins involved in chromatin organization, and histone modification. These coincidences suggest that if the HRC3 signature indeed plays a role in transcriptional regulation of genes, a possible mechanism of action could involve directing epigenetic modifications to specific genomic loci.

Consequently, studying the HRC3 signature may also lead towards an explanation why all genes in the collinear Hox clusters contain the homeobox domain. Preliminary enrichment-based analysis suggests that signatures related to HRC3 may be associated not only with homeoboxes, but possibly also with several other classes of DNA binding domains. The HRC3 signature may improve our understanding of certain aspects of gene regulation in developmental biology, and is likely to have impact onto other fields, including the study of cancers in which the regulation of developmental genes is disrupted.

## Methods

For Human, mouse and drosophila, we aligned coding DNA sequences obtained from the *Genbank* CCDS and CDS databases^56^ with the consensus homeobox sequence RRRKRTAYTRYQLLELEKEFLFNRYLTRRRRIELAHSLNLTERHIKIWFQNRMKWKEN using *tblastn*^57^ with an expectation threshold of 0.001, and selected the genes for which the alignment length was at least 140 nucleotides. For other species, the selection was based on the species-specific list of Hox genes present in the Homeobox Database^58^ that have sequences in *GenBank*. We predicted the HRC patterns using a modified sliding tetramer window algorithm^35^. The original sliding tetramer HRC prediction produces four values of HRC for each position-based on the four overlapping tetramers containing each pair of bases. Rather than using only one of them, we calculated their weighted average, with weights of 1/6, 1/3, 1/3 and 1/6 for the consecutive tetramers. The significance of the 3-bp period is calculated based on the value of the periodogram computed for the 180-bp homeobox sequence at T = 3 bp and Fisher’s test for single frequency^36^,^37^,^59^. The computer programs (written in Perl) to calculate the periodicity of the HRC pattern based on the DNA sequence and to compute the *P*_HRC180_(3) amplitude within identified homeoboxes are provided as Supplementary data (supplementary Program File SP1).

To compare the HRC3 motifs outside of the homeobox with ultraconserved coding regions (UCRs) (Suppl. Figure 4), we computed the periodogram power at 3 bp, *P*_HRC100_(3), over intervals of 100 bp centred on every position in the sequence. We defined periodic HRC intervals as those with *P*_HRC100_(3) equal at least 1.8 over at least 80 consecutive positions in the sequence. These data are overlaid on the UCRs in HoxA, HoxB and HoxC genes reported by ref. 22. To verify that the HRC3 signature is not a consequence of the coding sequence of the homeobox, we calculated the HRC patterns in a family of simulated homeobox sequences coding for the same homeodomain as in the actual genes. We generated the simulated sequences by randomly choosing codons for each amino acid from a distribution reflecting the actual codon frequencies in the entire coding genome (Suppl. Table S3). The lists of homeobox genes in the ANTP, LIM, POU, ZF, PRD and TALE classes are derived from the HomeoDB2 database^58^.

The enrichments of ENCODE binding sites have been calculated using BEDTools suite of utilities for comparing genomic features^60^. To compute the overlap between features (peaks of HRC3 and chromosomal position of DBDs for each family), we used the “*closest*” option of bedtools with the human genome hg19 and chose features for which the distance is zero (at least one overlapping position).

## Additional Information

**How to cite this article**: Fongang, B. *et al*. A Conserved Structural Signature of the Homeobox Coding DNA in HOX genes. *Sci. Rep.* **6**, 35415; doi: 10.1038/srep35415 (2016).

## Supplementary Material

Supplementary Information

## Figures and Tables

**Table 1:** The average GC content of the coding sequences in the different regions of Hox genes.

Species	GC 5′ region	GC Homeobox	GC 3′ region	5′ > Hbx pval	3′ > Hbx pval
Human	0.654+/−0.058	0.524+/−0.057	0.624+/−0.068	1.6e-12	6.6e-7
Mouse	0.639+/−0.056	0.528+/−0.052	0.605+/−0.066	1.3e-14	3.7e-6
Fly	0.619+/−0.021	0.542+/−0.031	0.606+/−0.036	4.0e-4	2.7e-2

The GC content within the homeobox is significantly lower than outside of it; a pattern preserved between vertebrate and invertebrate animals. In the averages we included coding regions 3′ of the homeobox only if they were at least 60 bp long. The significances (t-test) of the differences are listed in the last two columns.

**Table 2:** The HRC3 signature in mouse and human Hox genes.

Gene	Mouse			Human		
	P_HRC3_	P-value	GC	P_HRC3_	P-value	GC
HOXA1	5.540	3.90E-03	0.54	5.995	2.50E-03	0.55
HOXA2	3.540	2.90E-02	0.50	3.771	2.30E-02	0.49
HOXA3	10.684	2.30E-05	0.61	10.635	2.40E-05	0.60
HOXA4	10.599	2.50E-05	0.57	12.757	2.90E-06	0.58
HOXA5	5.079	6.20E-03	0.49	4.617	9.90E-03	0.49
HOXA6	10.689	2.30E-05	0.57	11.736	8.00E-06	0.61
HOXA7	14.362	5.80E-07	0.59	15.833	1.30E-07	0.61
HOXA9	7.268	7.00E-04	0.53	7.464	5.70E-04	0.51
HOXA10	8.846	1.40E-04	0.50	9.232	9.80E-05	0.51
HOXA11	5.735	3.20E-03	0.46	6.545	1.40E-03	0.46
HOXA13	*1.517*	*2.20E-01*	0.47	*1.000*	*3.70E-01*	0.45
HOXB1	6.128	2.20E-03	0.56	4.615	9.90E-03	0.53
HOXB2	6.449	1.60E-03	0.59	7.125	8.10E-04	0.59
HOXB3	11.396	1.10E-05	0.59	13.075	2.10E-06	0.60
HOXB4	13.465	1.40E-06	0.60	11.062	1.60E-05	0.60
HOXB5	8.118	3.00E-04	0.56	11.100	1.50E-05	0.60
HOXB6	11.421	1.10E-05	0.59	11.195	1.40E-05	0.59
HOXB7	11.473	1.00E-05	0.56	11.789	7.60E-06	0.57
HOXB8	7.122	8.10E-04	0.54	6.490	1.50E-03	0.54
HOXB9	7.762	4.30E-04	0.48	7.240	7.20E-04	0.47
HOXB13	5.420	4.40E-03	0.54	6.938	9.70E-04	0.57
HOXC4	7.682	4.60E-04	0.53	7.102	8.20E-04	0.52
HOXC5	11.849	7.10E-06	0.51	11.590	9.30E-06	0.51
HOXC6	8.671	1.70E-04	0.56	9.285	9.30E-05	0.57
HOXC8	4.261	1.40E-02	0.48	4.004	1.80E-02	0.47
HOXC9	9.494	7.50E-05	0.49	10.092	4.10E-05	0.49
HOXC10	4.892	7.50E-03	0.42	4.892	7.50E-03	0.42
HOXC11	5.699	3.30E-03	0.46	7.817	4.00E-04	0.47
HOXC12	9.925	4.90E-05	0.51	7.857	3.90E-04	0.52
HOXC13	3.359	3.50E-02	0.56	4.255	1.40E-02	0.57
HOXD1	4.137	1.60E-02	0.45	3.541	2.90E-02	0.46
HOXD3	12.070	5.70E-06	0.59	8.328	2.40E-04	0.57
HOXD4	6.594	1.40E-03	0.51	6.046	2.40E-03	0.50
HOXD8	*2.260*	*1.00E-01*	0.46	*0.653*	*5.20E-01*	0.44
HOXD9	5.634	3.60E-03	0.50	5.397	4.50E-03	0.49
HOXD10	8.345	2.40E-04	0.48	7.509	5.50E-04	0.47
HOXD11	6.070	2.30E-03	0.45	6.389	1.70E-03	0.45
HOXD12	9.766	5.70E-05	0.50	8.711	1.60E-04	0.50
HOXD13	3.192	4.10E-02	0.46	*2.803*	*6.10E-02*	0.45

Columns contain gene name, the HRC3 amplitude PHRC3, its significance and the GC content in mouse and in human for homeoboxes of 39 Hox genes.

**Table 3:** The HRC3 signature in Hox genes of metazoan species.

Species	Type	Hox genes					Median	
		N_All	P_HRC3_ > 3	%	P_HRC3_ > 6	%	P_HRC3_ > 3	p-value
*D. melanogaster*	S	8	7	88	7	86	10.1	4.23 × 10^−5^
*T. castaneum*	S	8	7	88	7	86	5.2	0.00552
*C. intestinalis*	U	9	5	56	0	0	4.1	0.01663
*D. rerio*	V	47	45	96	14	30	4.29	0.01373
*G. gallus*	V	29	16	55	10	34	4.95	0.00707
*H. sapiens*	V	39	36	92	27	69	7.24	0.00814
*M. musculus*	V	39	37	95	25	64	7.27	0.0005
*O. vulgaris*	M	8	5	62	1	12	3.48	0.0309

Columns contain species, the number of Hox genes considered, absolute and relative numbers of Hox genes with significant (P_HRC3_ > 3; p < 0.05) and highly significant (P_HRC3_ > 6.0; p < 0.00248), the median HRC3 amplitude P_HRC3_, and median significance for the species. “Organism Types” in column 2 are as follows: S-segmented invertebrate, V-vertebrate, M-mollusk, U-unsegmented invertebrate. Detailed information on individual genes is provided in Supplementary Table S4.

**Table 4:** Transcription Factor Binding Sites (TFBS) from the ENCODE project with peaks overlapping and non overlapping HRC3 in human genome version hg19.

TFBS	Binding Sites	HRC3 Overlap	HRC3 Ratio	SIM Fold	SIM Min	SIM Median	SIM Mean	SIM Max
Examples of DNA binding proteins significantly enriched in HRC3 loci								
* EZH2*	14818	2028	0.1368	6.0864	283	333.5	333.2	388
* RBBP5*	19205	2121	0.1104	5.9445	299	358.5	356.8	406
* SUZ12*	5772	598	0.1036	8.0236	49	74	74.53	110
* SAP30*	8399	794	0.0945	6.7345	84	118.5	117.9	157
* HDAC1*	10390	945	0.0909	6.2582	121	149	151	198
* PHF8*	17247	1494	0.0866	5.8087	212	256.5	257.2	308
* UBTF*	13613	1131	0.0830	6.8173	132	166.5	165.9	206
* HMGN3*	13061	1034	0.0791	5.7797	137	177.5	178.9	217
* E2F1*	17997	1392	0.0773	5	219	278	278.4	327
* KDM5B*	12943	963	0.0744	5.3233	139	180	180.9	217
* CHD1*	16981	1254	0.0738	5.0240	206	247	249.6	305
* SMARCB1*	8485	625	0.0736	4.4770	97	141	139.6	184
* HDAC6*	1110	81	0.0729	8.8621	2	9	9.14	18
* SP4*	5352	382	0.0713	4.4444	59	85	85.95	117
* CTBP2*	6537	459	0.0702	4.6181	63	99	99.39	143
* CTCF*	162209	4733	0.0291	3.1590	1385	1496	1498	1594
Examples of TFs with no enrichment of HRC3 in binding loci								
* FOS*	131528	1047	0.0079	1.0335	918	1012	1013	1114
* BATF*	32419	239	0.0073	1.2513	154	191.5	191	234
* MAFF*	47076	346	0.0073	1.0282	285	336	336.5	401
* MAFK*	84087	618	0.0073	1.0363	540	596.5	596.3	650
* RPC155*	2806	19	0.0067	0.5521	21	34	34.41	54
* FOXA1*	89906	604	0.0067	0.9596	532	630	629.4	707
* BDP1*	791	5	0.0063	0.8605	0	5.5	5.81	13
* PRDM1*	4574	26	0.0056	0.8373	15	31	31.05	47
* FOXA2*	40866	227	0.0055	0.8156	225	277	278.3	323
* FAM48A*	4087	16	0.0039	0.644	13	24	24.82	43

For each TFBS the total number of peaks is represented as well as peaks overlapping with HRC3. The HRC3 Ratio is the proportion of HRC3 peaks present in the TFBS Chip-Seq data. To compute the significance of the testing, each TFBS was shuffled 100 times and the number of peaks overlapping with the HRC3 data was computed using bedtools. The median number of overlapping peaks (SIM Median column) then represents the number of overlapping due to random effect. Typically, statistically significant overlapping peaks (top) will have their SIM Median values lower than their HRC3 Overlap. The complete list of the 161 TFBSs is presented in Table S7.
