# Supplementary material for: A Conserved Structural Signature of the Homeobox Coding DNA in HOX genes
Source: Sci Rep. 2016 Oct 14;6:35415. doi: 10.1038/srep35415 (PMC5064350; doi:10.1038/srep35415)
Supplement: Supplementary Information [file srep35415-s1.pdf]

A Conserved Structural Signature of the Homeobox Coding DNA in HOX genes

Bernard Fongang[1,2], Fanping Kong[1], Surendra Negi[1], Braun Werner[1] and Andrzej Kudlicki[1,2,3] *

[1] Department of Biochemistry and Molecular Biology, University of Texas Medical Branch, Galveston, TX, USA

[2] Institute for Translational Sciences, University of Texas Medical Branch, Galveston, TX, USA

[3] Sealy Center for Molecular Medicine, University of Texas Medical Branch, Galveston, TX, USA

[*] Corresponding author

Email address: askudlic@utmb.edu (AK)

Supporting information

Supplementary Figure S1. Dependence between codon selection effect and the magnitude of the HRC3 periodic signal. X-axis: P_{HRC3} of the 180-bp sequence aligned with the homeobox. Y-axis: Fraction of simulated synonymous sequences (out of 1,000 for each gene) with P_{HRC3} smaller than in the actual mouse gene.

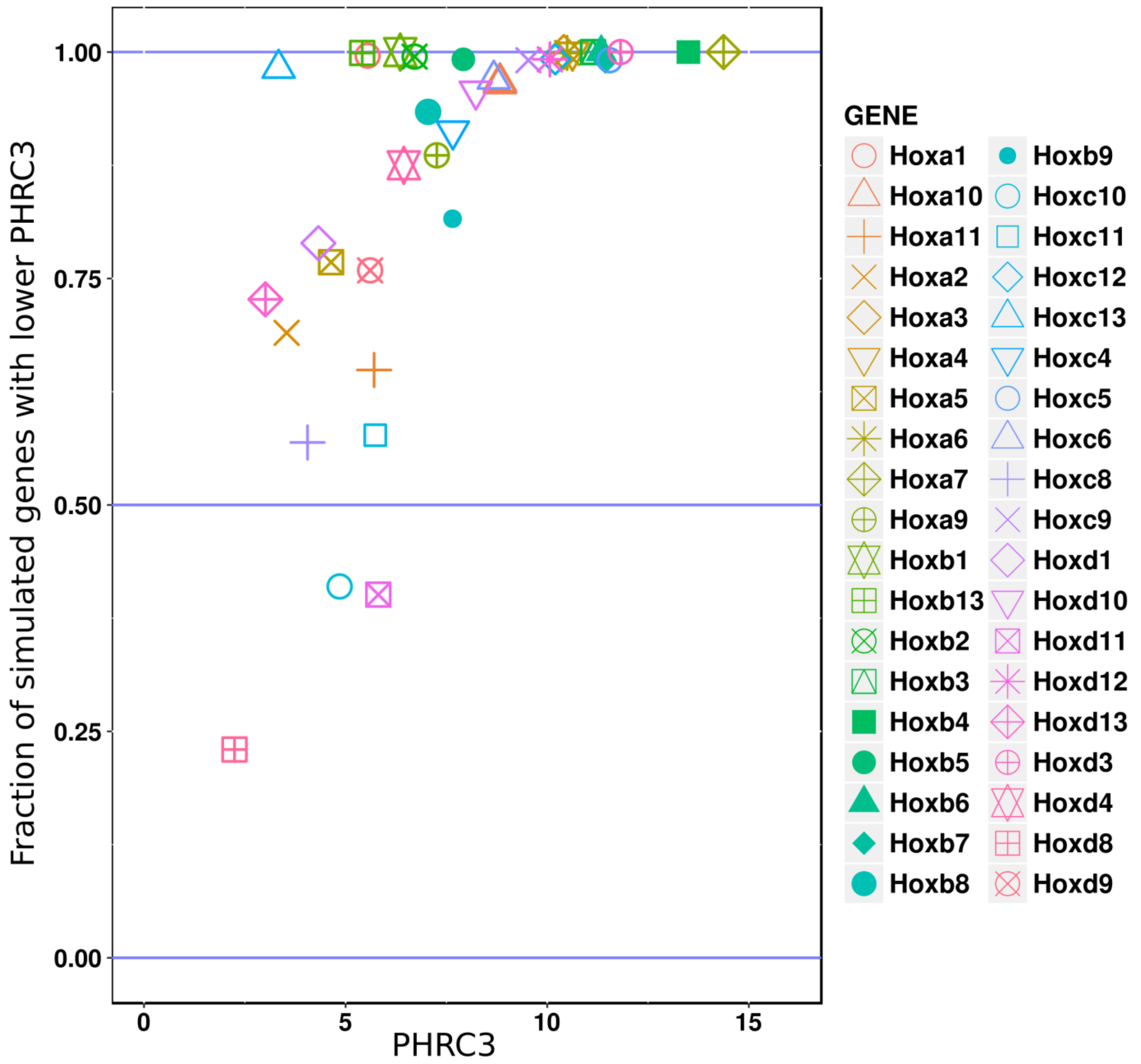

Supplementary Figure S2. Codon bias in homeoboxes of mouse, human and Drosophila. Left panel: the ratio of codon usage in homeoboxes of mouse Hox genes to usage in all mouse proteins (x-axis) is almost identical to the ratio in human (y-axis). Each dot represents one codon. Right panel: the same comparison between codon biases in mouse and fly shows significant correlation.

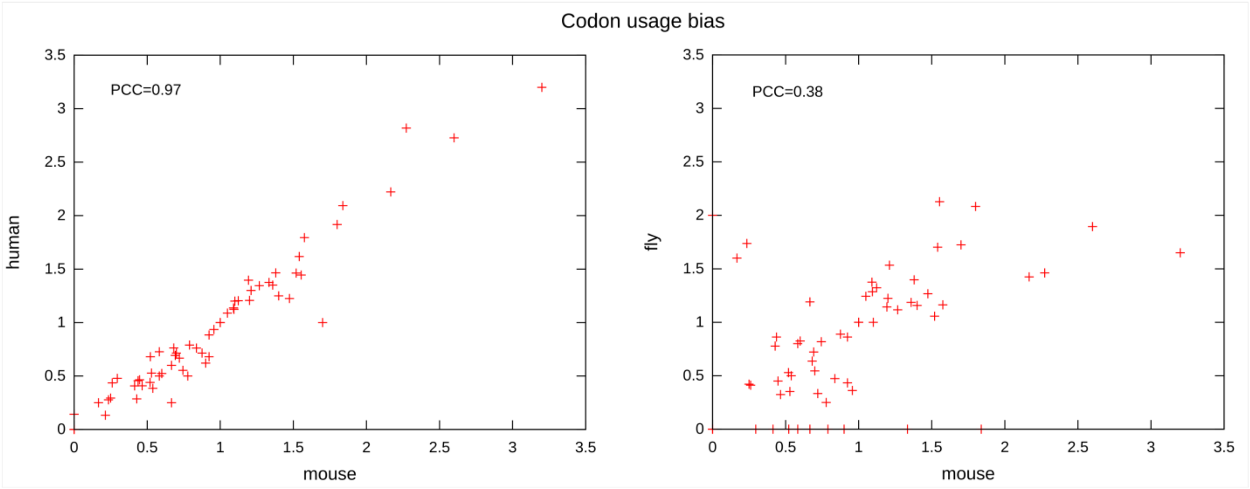

Supplementary Figure S3. Dependence between clustering of mouse homeobox-containing genes and the magnitude of the HRC3 periodic signal. X-axis: distance to the nearest homeobox gene on the same chromosome (distance between centers of coding sequences according to mm10). Y-axis: P_{HRC3} of the 180-bp sequence aligned with the homeobox.

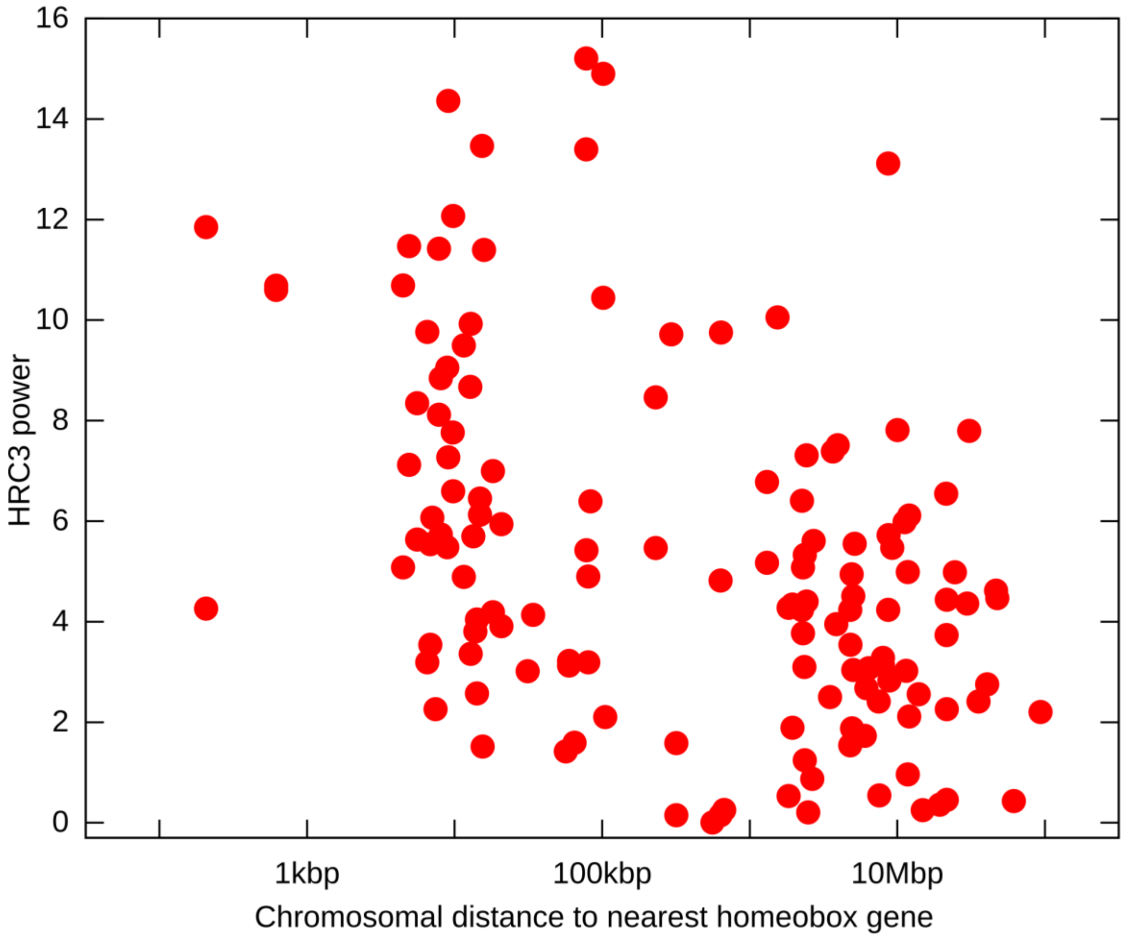

Supplementary Figure S4. Gene Ontology Enrichment for mouse genes with PHRC3 > 10. The figure shows the top enriched Molecular Function and Cellular Component.

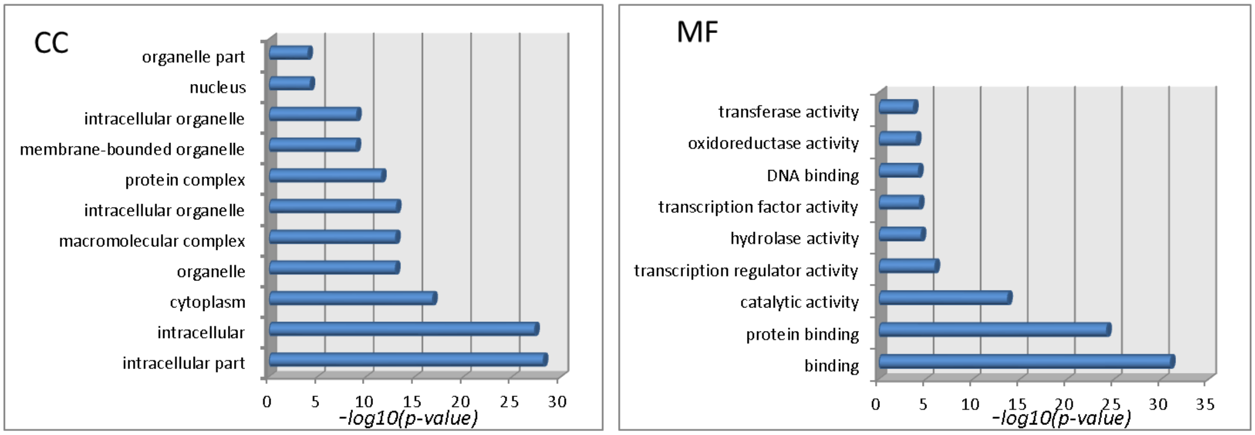

Supplementary Figure S5. Predicted nucleosome occupancy in Hox genes, plotted against the position relative to the homeobox. The blue curve represents the nucleosome occupancy, averaged over all Hox genes (aligned to homeobox position), errorbars correspond to the standard deviation of gene-to-gene variance. The positions of the homeobox and typical locations of coding sequences are marked by symbols at bottom of the panel.

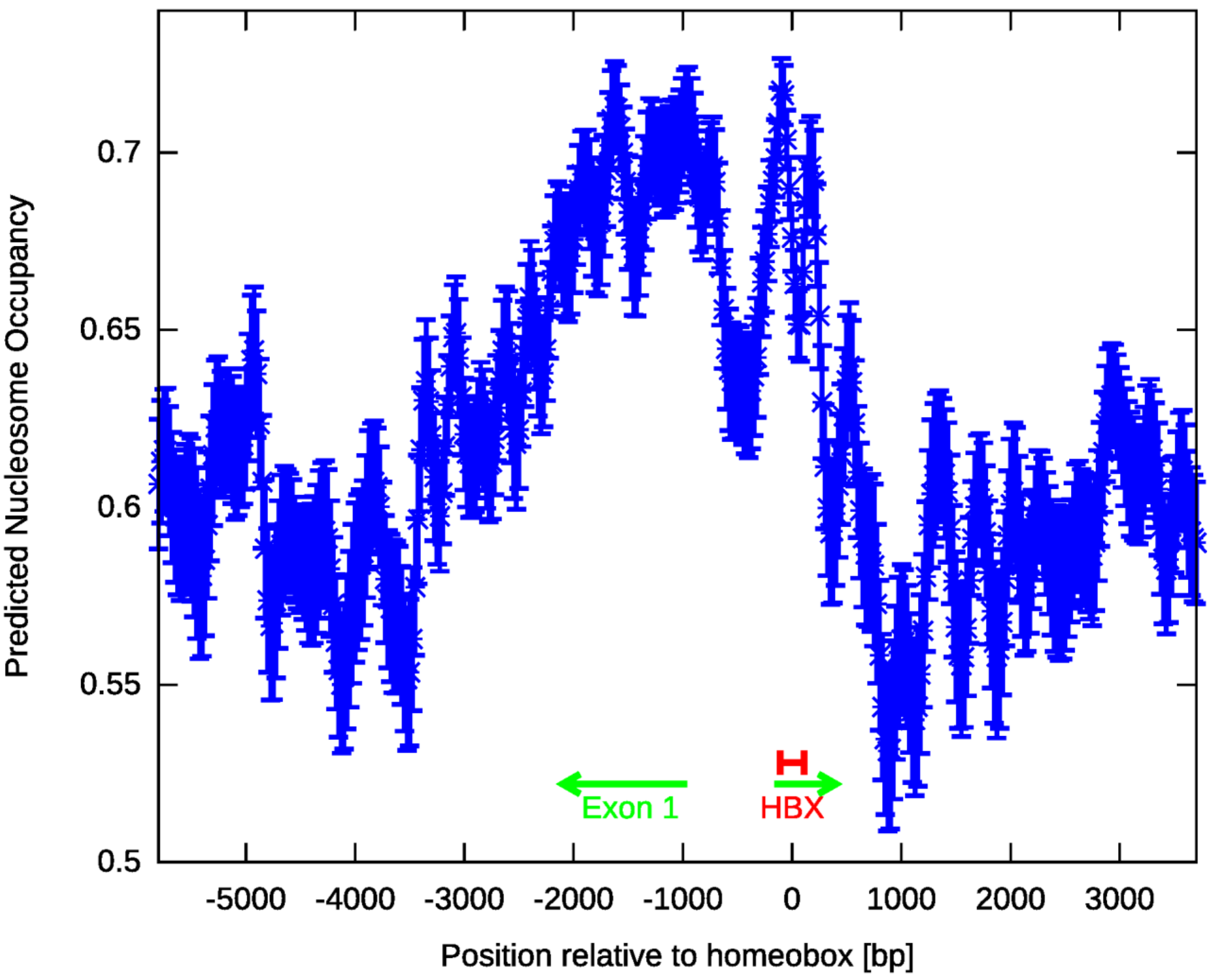

Supplementary Figure S6. Positions of HRC3 and UCR regions in mouse Hox genes.

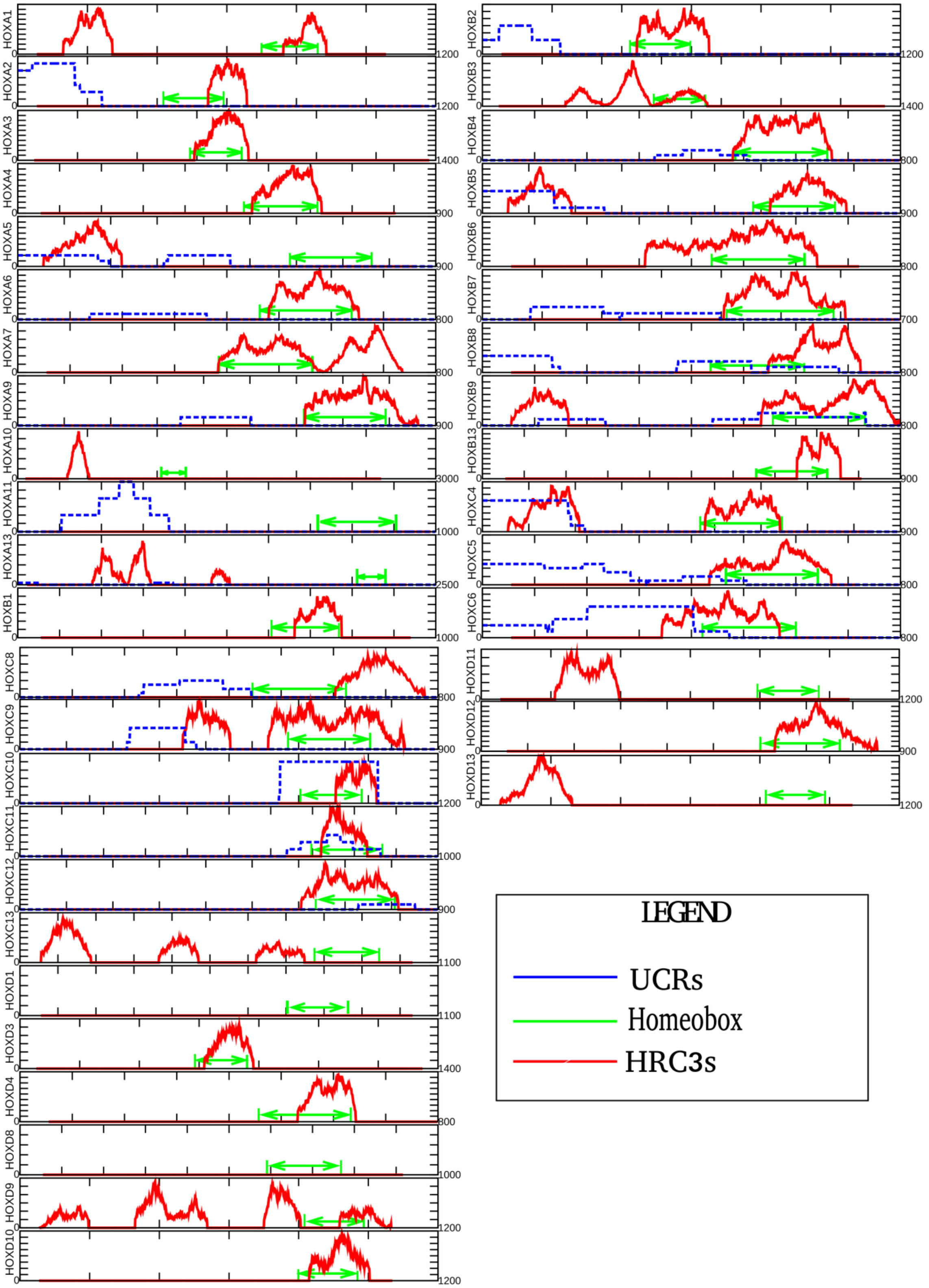

Table S1: Codon selection effect in homeoboxes of mouse Hox genes. Left column: gene name. Center: HRC3 signal (P_{HRC3}) in the homeobox. Right column: Fraction of simulated homeobox sequences with P_{HRC3} higher than in the actual gene.

GENE	P_{HRC3}(hbx)	p(no-codon-bias)
Hoxa1	5.546	0.004
Hoxa10	8.827	0.035
Hoxa10	8.827	0.033
Hoxa11	5.706	0.351
Hoxa2	3.536	0.31
Hoxa3	10.411	0
Hoxa4	10.625	0
Hoxa5	4.639	0.232
Hoxa6	10.494	0.001
Hoxa7	14.373	0
Hoxa9	7.262	0.114
Hoxb1	6.354	0
Hoxb13	5.411	0.001
Hoxb2	6.711	0.005
Hoxb3	11.136	0
Hoxb4	13.501	0
Hoxb5	7.919	0.008
Hoxb6	11.339	0
Hoxb7	11.437	0.011
Hoxb8	7.046	0.066
Hoxb9	7.652	0.184
Hoxc10	4.851	0.59
Hoxc11	5.735	0.423
Hoxc12	10.199	0.008
Hoxc13	3.338	0.018
Hoxc4	7.659	0.087
Hoxc5	11.549	0.009
Hoxc6	8.674	0.03
Hoxc8	4.055	0.431
Hoxc9	9.534	0.009
Hoxd1	4.325	0.211
Hoxd10	8.230	0.043
Hoxd11	5.811	0.599
Hoxd12	10.064	0.008
Hoxd13	3.011	0.273
Hoxd3	11.818	0
Hoxd4	6.442	0.125
Hoxd8	2.244	0.77
Hoxd9	5.608	0.241

Table S2. Codon frequencies genome wide and in homeoboxes of Hox genes.
The genome-wide averages were used in simulating random synonymous mutations of the homeobox coding sequences.

AA	Codon	Human		Drosophila		Mouse	
		Genome	Homeobox	Genome	Homeobox	Genome	Homeobox
*	TAG	0.24	0	0.33	0	0.22	0
*	TAA	0.3	0	0.41	0	0.26	0
*	TGA	0.47	0	0.25	0	0.52	0
A	GCG	0.11	0.3	0.19	0.36	0.1	0.26
A	GCA	0.23	0.1	0.17	0.07	0.23	0.06
A	GCT	0.27	0.11	0.19	0	0.29	0.12
A	GCC	0.4	0.49	0.45	0.57	0.38	0.56
C	TGT	0.46	0.21	0.29	0.25	0.48	0.21
C	TGC	0.54	0.79	0.71	0.75	0.52	0.79
D	GAT	0.46	0.22	0.53	0	0.44	0.13
D	GAC	0.54	0.78	0.47	1	0.56	0.87
E	GAA	0.42	0.3	0.33	0.18	0.4	0.28
E	GAG	0.58	0.7	0.67	0.82	0.6	0.72
F	TTT	0.46	0.35	0.38	0.18	0.43	0.36
F	TTC	0.54	0.65	0.62	0.82	0.57	0.64
G	GGT	0.16	0.04	0.21	0.25	0.18	0.12
G	GGG	0.25	0.17	0.07	0	0.23	0.12
G	GGA	0.25	0.17	0.29	0.25	0.26	0.24
G	GGC	0.34	0.61	0.43	0.5	0.33	0.52
H	CAT	0.42	0.22	0.4	0.33	0.4	0.24
H	CAC	0.58	0.78	0.6	0.67	0.6	0.76
I	ATA	0.17	0.05	0.19	0.08	0.16	0.04
I	ATT	0.36	0.19	0.34	0.12	0.34	0.18
I	ATC	0.47	0.76	0.47	0.8	0.5	0.77
K	AAA	0.43	0.38	0.3	0.13	0.39	0.36
K	AAG	0.57	0.62	0.7	0.87	0.61	0.64
L	CTA	0.07	0.05	0.09	0.08	0.08	0.07
L	TTA	0.08	0.02	0.05	0.08	0.06	0.01
L	TTG	0.13	0.09	0.18	0.13	0.13	0.09
L	CTT	0.13	0.05	0.1	0.05	0.13	0.07
L	CTC	0.2	0.24	0.15	0.15	0.2	0.22
L	CTG	0.4	0.54	0.43	0.51	0.39	0.53
M	ATG	1	1	1	1	1	1
N	AAT	0.47	0.26	0.44	0.36	0.43	0.32
N	AAC	0.53	0.74	0.56	0.64	0.57	0.68
P	CCG	0.11	0.11	0.29	0.5	0.1	0.17

**P**	**CCA**	0.28	0.04	0.25	0.5	0.28	0
**P**	**CCT**	0.29	0.18	0.13	0	0.3	0.27
**P**	**CCC**	0.32	0.67	0.33	0	0.31	0.57
**Q**	**CAA**	0.27	0.18	0.3	0.1	0.25	0.18
**Q**	**CAG**	0.73	0.82	0.7	0.9	0.75	0.82
**R**	**CGT**	0.08	0.04	0.16	0.04	0.09	0.07
**R**	**CGA**	0.11	0.08	0.15	0.12	0.12	0.07
**R**	**CGC**	0.18	0.4	0.33	0.47	0.18	0.39
**R**	**CGG**	0.2	0.26	0.15	0.23	0.19	0.23
**R**	**AGG**	0.21	0.16	0.11	0.07	0.22	0.15
**R**	**AGA**	0.21	0.06	0.09	0.07	0.21	0.09
**S**	**TCG**	0.05	0.16	0.2	0.33	0.05	0.16
**S**	**AGT**	0.15	0.09	0.14	0	0.15	0.1
**S**	**TCA**	0.15	0.02	0.09	0.33	0.14	0.03
**S**	**TCT**	0.19	0.15	0.08	0	0.19	0.15
**S**	**TCC**	0.22	0.25	0.24	0.33	0.22	0.24
**S**	**AGC**	0.24	0.33	0.25	0	0.24	0.32
**T**	**ACG**	0.11	0.31	0.26	0.38	0.11	0.25
**T**	**ACT**	0.25	0.11	0.17	0.09	0.25	0.13
**T**	**ACA**	0.28	0.13	0.2	0.09	0.29	0.13
**T**	**ACC**	0.36	0.45	0.38	0.44	0.35	0.49
**V**	**GTA**	0.12	0.06	0.11	0	0.12	0.07
**V**	**GTT**	0.18	0.05	0.19	0.33	0.17	0.04
**V**	**GTC**	0.24	0.46	0.24	0.5	0.25	0.45
**V**	**GTG**	0.46	0.43	0.47	0.17	0.46	0.44
**W**	**TGG**	1	1	1	1	1	1
**Y**	**TAT**	0.44	0.18	0.37	0.12	0.43	0.2
**Y**	**TAC**	0.56	0.82	0.63	0.88	0.58	0.8

Table S3. HRC3 signal (P_{HRC3}) in the homeobox of 158 mouse genes. For genes with more than one homeobox, the highest value is reported.

GENE	P_{HRC3}
ADNP	0.548
ALX1	0.723
ALX3	4.436
ALX4	6.113
ARX	7.811
BARHL1	4.339
BARHL2	4.238
BARX1	3.540
BARX2	5.551
BSX	5.081
CDX1	4.942
CDX2	2.829
CDX4	2.412
DBX1	4.983
DBX2	1.877
DLX1	4.045
DLX2	2.574
DLX3	3.910
DLX4	5.941
DLX6	3.733
EMX1	5.464
EMX2	1.865
EN1	7.794
EN2	6.778
EVX1	1.419
EVX2	3.807
GBX1	7.387
GBX2	4.988
GSC2	4.468
GSC	8.466
GSX1	6.393
GSX2	3.281
HESX1	0.455
HHEX	2.676
HLX1	5.792
HMX1	7.309
HMX2	9.055
HMX3	5.482
HOXA10	8.846
HOXA11	5.735
HOXA13	1.517
HOXA1	5.540
HOXA2	3.540

HOXA3	10.684
HOXA4	10.599
HOXA5	5.079
HOXA6	10.689
HOXA7	14.362
HOXA9	7.268
HOXB13	5.420
HOXB1	6.128
HOXB2	6.449
HOXB3	11.396
HOXB4	13.465
HOXB5	8.118
HOXB6	11.421
HOXB7	11.473
HOXB8	7.122
HOXB9	7.762
HOXC10	4.892
HOXC11	5.699
HOXC12	9.925
HOXC13	3.359
HOXC4	7.682
HOXC5	11.849
HOXC6	8.671
HOXC8	4.261
HOXC9	9.494
HOXD10	8.345
HOXD11	6.070
HOXD12	9.766
HOXD13	3.192
HOXD1	4.137
HOXD3	12.070
HOXD4	6.594
HOXD8	2.260
HOXD9	5.634
IRX1	0.256
IRX2	0.151
IRX3	0.003
IRX4	4.817
IRX5	1.583
IRX6	0.151
ISL1	3.422
ISL2	5.180
ISX	4.511
LBX1	4.900
LBX2	4.189
LHX1	1.729
LHX2	6.407
LHX3	5.607

LHX4	0.544
LHX5	2.556
LHX6	4.231
LHX8	0.359
LMX1A	0.531
LMX1B	0.207
MEOX1	5.539
MEOX2	0.359
MIXL1	7.885
MNX1	5.172
MSX1	4.400
MSX2	1.242
NANOG	15.244
NKX2-1	13.395
NKX2-2	10.444
NKX2-3	10.053
NKX2-4	14.900
NKX2-5	13.116
NKX2-8	15.206
NKX3-1	6.051
NKX3-2	7.509
NKX6-1	1.541
NKX6-2	7.888
NKX6-3	4.619
NOBOX	2.831
NOTO	8.462
OTP	6.545
PAX3	0.961
PAX4	0.250
PAX6	2.116
PAX7	4.492
PBX4	3.040
PDX1	3.013
PHOX2A	4.360
PHOX2B	3.134
PITX1	5.328
PITX2	2.258
PKNOX2	3.950
POU1F1	0.203
POU2F1	4.278
POU2F2	5.471
POU2F3	3.769
POU3F1	5.724
POU3F2	2.201
POU3F3	2.413
POU3F4	0.317
POU5F1	4.236
POU6F1	3.100

PRRX1	0.872
PRRX2	1.889
RAX	6.757
SHOX2	2.754
SIX1	3.219
SIX2	2.302
SIX3	1.592
SIX4	3.128
SIX6	2.101
TLX1	3.191
TLX2	6.996
TLX3	5.982
VAX1	9.715
VAX2	9.752
VSX1	2.500
VSX2	3.021
ZFHX2	3.076
ZFHX3	7.615
ZFHX4	2.092

Table S4. HRC3 signal (P_{HRC3}) in the homeoboxes of Hox gene homologs in 9 species.

**Species**	**genes**	**P_{HRC3} (homeobox)**
Drosophila melanogaster	abdominal B (Abd-B)	10.53415
	abdominal A (abd-A)	8.68936
	antennapedia (Antp)	11.12226
	labial (lab)	8.45922
	Sex combs reduced (Scr)	12.24294
	proboscipedia (pb)	2.08449
	deformed (Dfd)	10.2099
	ultrabithorax (Ubx)	9.93209
Tribolium castaneum	ultrabithorax (Ubx)	6.07210
	abdominal-B (Abd-B)	25.3503
	abdominal-A (Abd-A)	16.3559
	labial (Lab)	2.93223
	cephalothorax (Cx)	5.12495
	tailup (Tup)	9.47753
	apterous	13.8430
	deformed (Dfd)	6.65465
Ciona intestinalis	Gsx (hoxa2)	5.34983
	homeobox 3	3.65200
	homeobox 5	4.68593
	homeobox 2	4.45378
	hox10	4.34395
	hox12	1.92251
	hox4 protein (hox4)	1.40696
	homeobox transcription factor Hox1 (hox1)	0.96320
	homeobox transcription factor Hox13	4.47517
	homeobox protein Hox-D9a	1.91843
	homeobox protein Hox-C12a	4.48495
Danio rerio	hoxa2b	6.86024
	hoxb4a	6.41559
	hoxc5a	8.14068
	hoxb8a	3.26335
	hoxa11a	4.62368
	hoxa11b	3.14277
	hoxa13b	0.71779
	hoxa3a	3.33942
	hoxb1a	3.25475
	hoxb6a	4.83156
	hoxc12b	5.99923

	hoxc1a	1.82119
	hoxc6a	3.54792
	hoxc6b	4.65939
	hoxa5a	3.04892
	hoxa9b	4.71964
	hoxb2a	3.74739
	hoxc13a	2.37678
	hoxc8a	3.40445
	hoxb9a	3.30898
	hoxa10b	4.61649
	hoxc9a	3.28506
	hoxb13a	1.39179
	hoxb1b	3.59115
	hoxa13a	1.74940
	hoxd4a	2.23032
	hoxc12a	4.28857
	hoxb10a	5.27857
	hoxd3a	5.07851
	hoxd11a	4.31152
	hoxb6b	2.92081
	hoxd9a	4.61037
	hoxd10a	4.40553
	hoxd13a	2.76603
	hoxc11a	2.45988
	hoxb5b	5.97228
	hoxa1a	5.75898
	hoxb7a	5.49090
	hoxc11b	4.09432
	hoxb5a	3.34043
	hoxa9a	6.22501
	hoxc4a	3.66810
	hoxb3a	6.54803
	hoxa4a,	6.99547
	hoxc13b	3.93846
	hoxd12a	7.42277
	hoxb8b	8.59841
	hoxc3a	6.89928
Equus caballus	HOXA1	7.39474
	HOXA10	10.2709
	HOXA11	9.11554
	HOXA13	1.91312
	HOXA2	7.12756

	HOXA3	13.7013
	HOXA5	6.47939
	HOXA6	13.776
	HOXA7	18.7119
	HOXA9	9.77966
	HOXB1	11.0165
	HOXB13	9.81453
	HOXB2	12.197
	HOXB3	15.2553
	HOXB5	14.2469
	HOXB6	16.7693
	HOXB7	14.0447
	HOXB8	4.96773
	HOXB9	7.36595
	HOXC10	5.15459
	HOXC11	8.13208
	HOXC12	11.3914
	HOXC13	7.59431
	HOXC4	7.15441
	HOXC6	10.0798
	HOXC8	6.89746
	HOXC9	10.7956
	HOXD1	3.99279
	HOXD10	7.98987
	HOXD11	5.82608
	HOXD12	12.3332
	HOXD13	5.37181
	HOXD3	13.4406
	HOXD8	3.91066
	HOXD9	10.1836
Gallus gallus	HOXD13	7.78881
	HOXC8	6.08995
	HOXD12	4.54616
	HOXA11	3.89827
	HOXD11	3.52714
	HOXA3	2.82485
	HOXA13	2.38836
	HOXB4	11.9577
	HOXD8	9.82665
	HOXA7	20.1458
	HOXB5	7.56777
	HOXA4	16.7574

	HOXA6	2.79517
	HOXC13	8.64768
	HOXC12	5.89744
	HOXB1	7.15487
	HOXD3	12.2120
	HOXD10	7.40990
	HOXD9	7.65610
	HOXB13	10.1321
	HOXB9	7.40080
	HOXC11	4.05507
	HOXB8	5.07964
	HOXB3	10.9702
Homo sapiens	HOXA1	5.995
	HOXA2	3.771
	HOXA3	10.635
	HOXA4	12.757
	HOXA5	4.617
	HOXA6	11.736
	HOXA7	15.833
	HOXA9	7.464
	HOXA10	9.232
	HOXA11	6.545
	HOXA13	*1.000*
	HOXB1	4.615
	HOXB2	7.125
	HOXB3	13.075
	HOXB4	11.062
	HOXB5	11.100
	HOXB6	11.195
	HOXB7	11.789
	HOXB8	6.490
	HOXB9	7.240
	HOXB13	6.938
	HOXC4	7.102
	HOXC5	11.590
	HOXC6	9.285
	HOXC8	4.004
	HOXC9	10.092
	HOXC10	4.892
	HOXC11	7.817
	HOXC12	7.857
	HOXC13	4.255

	HOXD1	3.541
	HOXD3	8.328
	HOXD4	6.046
	HOXD8	*0.653*
	HOXD9	5.397
	HOXD10	7.509
	HOXD11	6.389
	HOXD12	8.711
	HOXD13	*2.803*
Mus musculus	HOXA1	5.540
	HOXA2	3.540
	HOXA3	10.684
	HOXA4	10.599
	HOXA5	5.079
	HOXA6	10.689
	HOXA7	14.362
	HOXA9	7.268
	HOXA10	8.846
	HOXA11	5.735
	HOXA13	*1.517*
	HOXB1	6.128
	HOXB2	6.449
	HOXB3	11.396
	HOXB4	13.465
	HOXB5	8.118
	HOXB6	11.421
	HOXB7	11.473
	HOXB8	7.122
	HOXB9	7.762
	HOXB13	5.420
	HOXC4	7.682
	HOXC5	11.849
	HOXC6	8.671
	HOXC8	4.261
	HOXC9	9.494
	HOXC10	4.892
	HOXC11	5.699
	HOXC12	9.925
	HOXC13	3.359
	HOXD1	4.137
	HOXD3	12.070

	HOXD4	6.594
	HOXD8	*2.260*
	HOXD9	5.634
	HOXD10	8.345
	HOXD11	6.070
	HOXD12	9.766
	HOXD13	3.192
Octopus vulgaris	Lab	11.157
	Antp	3.6293
	Lox2	2.36755
	Lox4	3.77027
	Lox5	0.7562
	Post1	3.06863
	Post2	3.21508
	Scr	1.28422

Table S5. Gene Ontology enrichment annotation of mouse genes with HRC3 signal (P_{HRC3}) > 10.0 anywhere within the DNA sequence of the gene (introns and exons).

Ontology	GO term	# genes	P-value	FDR
Molecular function	0005488~binding	3767	1.1e-31	2.1e-28
	0005515~protein binding	2063	6.5e-25	6.1e-22
	0003824~catalytic activity	1770	2.1e-14	1.3e-11
	0030528~transcription regulator activity	442	1.1e-6	5.2e-04
	0016787~hydrolase activity	758	3.2e-5	1.2e-02
	0003700~transcription factor activity	287	4.9e-5	1.5e-02
	0003677~DNA binding	614	6.2e-5	1.7e-02
	0016491~oxidoreductase activity	267	1.04e-4	2.4e-02
	0016740~transferase activity	590	2.1e-4	4.3e-02
Cellular Component	0044424~intracellular part	3228	6.4e-29	8.9e-32
	0005622~intracellular	3348	4.9e-28	1.4e-30
	0005737~cytoplasm	2211	1.5e-17	6.4e-20
	0043226~organelle	2709	1.0e-13	5.7e-16
	0032991~macromolecular complex	943	9.6e-14	6.5e-16
	0043229~intracellular organelle	2707	8.0e-14	7.1e-16
	0043234~protein complex	759	2.7e-12	2.6e-14
	0043227~membrane-bounded organelle	2389	1.1e-09	1.2e-11
	0043231~intracellular membrane-bounded organelle	2387	9.7e-10	1.2e-11
	0005634~nucleus	1430	5.9e-05	8.2e-07
	0044422~organelle part	1083	9.9e-05	1.5e-06
	0044446~intracellular organelle part	1074	1.4e-04	2.4e-06
	0044444~cytoplasmic part	1382	1.8e-04	3.2e-06
	0043232~intracellular non-membrane-bounded organelle	660	9.4e-04	1.8e-05
	0043228~non-membrane-bounded organelle	660	9.4e-04	1.8e-05
	0044428~nuclear part	437	3.0e-03	6.2e-05
	0005856~cytoskeleton	397	3.9e-03	8.8e-05
	0005829~cytosol	207	5.0e-03	1.2e-04
	0044459~plasma membrane part	560	5.1e-03	1.3e-04
	0005576~extracellular region	566	2.9e-02	7.7e-04
	0005886~plasma membrane	951	2.9e-02	8.2e-04
Biological Process	GO:0009987~cellular process	3160	1.7e-38	8.5e-35
	GO:0008152~metabolic	2389	1.1e-19	2.7e-16
	GO:0044238~primary metabolic process	2125	1.4e-17	2.4e-14
	GO:0044237~cellular metabolic process	1984	4.8e-13	6.1e-10
	GO:0048856~anatomical structure development	807	5.1e-13	5.2e-10
	GO:0048731~system development	758	8.9e-13	7.6e-10
	GO:0048522~positive regulation of cellular process	514	8.5e-12	6.2e-09
	GO:0048518~positive regulation of biological process	569	3.8e-11	2.4e-08
	GO:0007275~multicellular organismal development	909	4.7e-11	2.6e-08
	GO:0048513~organ	625	5.9e-11	3.0e-08

	GO:0009653~anatomical structure morphogenesis	436	1.9e-10	9.1e-08
	GO:0032502~developmental process	975	3.0e-10	1.3e-07
	GO:0043170~macromolecule metabolic process	1708	1.9e-08	7.6e-06
	GO:0009888~tissue development	260	2.3e-08	8.4e-06
	GO:0008104~protein localization 297	297	9.1e-08	3.1e-05
	GO:0031325~positive regulation of cellular metabolic	257	2.4e-07	7.5e-05
	GO:0009893~positive regulation of metabolic process	270	3.0e-07	8.9e-05
	GO:0044260~cellular macromolecule metabolic process	1519	3.0e-07	8.4e-05
	GO:0031328~positive regulation of cellular biosynthetic	224	3.4e-07	9.2e-05
	GO:0010557~positive regulation of macromolecule biosynthetic	216	3.9e-07	9.9e-05
	GO:0007399~nervous system development	328	4.0e-07	9.3e-05
	GO:0010628~positive regulation of gene expression	201	4.2e-07	9.4e-05
	GO:0048519~negative regulation of biological process	486	4.6e-07	9.9e-05
	GO:0009891~positive regulation of biosynthetic process	225	4.9e-07	9.9e-05
	GO:0033036~macromolecule localization	343	6.4e-07	1.3e-04
	GO:0030154~cell differentiation	565	6.7e-07	1.3e-04
	GO:0048869~cellular developmental process	587	6.8e-07	1.2e-04
	GO:0042127~regulation of cell proliferation	217	9.0e-07	1.5e-04
	GO:0009058~biosynthetic process	1047	1.6e-06	2.6e-04
	GO:0009887~organ morphogenesis 235	235	1.7e-06	2.7e-04
	GO:0006357~regulation of transcription from RNA polymerase II promoter	242	2.2e-6	3.4e-04
	GO:0044249~cellular biosynthetic process	1013	3.3e-06	4.8e-04
	GO:0016043~cellular component organization	663	4.1e-06	5.8e-04
	GO:0019222~regulation of metabolic process	1000	4.1e-06	5.7e-04
	GO:0009892~negative regulation of metabolic process	212	4.4e-06	5.9e-04
	GO:0045184~establishment of protein localization	254	4.5e-06	5.9e-04
	GO:0010605~negative regulation of macromolecule metabolic process	202	4.9e-6	6.3e-4
	GO:0015031~protein transport	252	5.0e-6	6.3e-04
	GO:0050793~regulation of developmental process	223	6.1e-06	7.4e-04
	GO:0031323~regulation of cellular metabolic process	945	1.3e-05	1.5e-03
	GO:0034641~cellular nitrogen compound metabolic process	1043	2.2e-05	2.6e-03
	GO:0048523~negative regulation of cellular process	428	2.6e-05	2.9e-03
	GO:0010467~gene expression	872	3.2e-05	3.6e-03
	GO:0055114~oxidation reduction	254	3.4e-05	3.7e-03
	GO:0006807~nitrogen compound metabolic process	1068	4.5e-05	4.8e-03
	GO:0060255~regulation of macromolecule metabolic process	907	4.8e-05	5.0e-03
	GO:0009790~embryonic development	252	5.3e-05	5.4e-03

	GO:0006629~lipid metabolic process	260	6.8e-05	6.8e-03
	GO:0022008~neurogenesis	206	7.3e-05	7.2e-03
	GO:0080090~regulation of primary metabolic process	897	1.2e-04	1.1e-02
	GO:0019538~protein metabolic process	878	1.4e-04	1.3e-02
	GO:0051239~regulation of multicellular organismal process	285	1.4e-04	1.3e-02
	GO:0006139~nucleobase, nucleoside, nucleotide and nucleic acid metabolic process	962	1.4e-4	1.3e-02
	GO:0048468~cell development	229	1.5e-04	1.4e-02
	GO:0065009~regulation of molecular function	206	1.7e-04	1.5e-02
	GO:0043067~regulation of programmed cell death	211	2.0e-04	1.7e-02
	GO:0051179~localization	889	2.4e-04	2.0e-02
	GO:0010941~regulation of cell death	211	2.8e-04	2.3e-02
	GO:0022414~reproductive process	230	2.9e-04	2.4e-02
	GO:0031326~regulation of cellular biosynthetic process	827	2.9e-04	2.4e-02
	GO:0010646~regulation of cell communication	280	3.3e-04	2.6e-02
	GO:0000003~reproduction	231	3.3e-04	2.6e-02
	GO:0042981~regulation of apoptosis	207	3.5e-04	2.6e-02
	GO:0009889~regulation of biosynthetic process	828	4.0e-04	3.0e-02
	GO:0010468~regulation of gene expression	810	6.7e-04	4.9e-02
	GO:0009966~regulation of signal transduction	241	6.9e-04	5.0e-02
	GO:0034645~cellular macromolecule biosynthetic process	800	7.0e-04	5.0e-02
	GO:0006350~transcription	601	7.1e-04	5.0e-02

DNA Binding Domain	Domain Sites	HRC3 Overlap	HRC3 Ratio	SIM Fold	SIM Min	SIM Median	SIM Mean	SIM Max
Forkhead domain	44	30	0.68	78.9	0	0	0.38	3
Homeobox domain	195	66	0.34	23.1	0	2.5	2.86	11
Basic helix-loop-helix	101	30	0.30	23.1	0	1	1.3	7
c2h2 Zinc Finger domain	619	134	0.22	15.7	1	8	8.51	21
Ets domain	15	4	0.27	18.2	0	0	0.22	4
Pou domain	16	0	0	0	0	0	0.05	1

Table S6: DNA Binding Domain (DBD) families with peaks overlapping the HRC3 signature in human genome (version hg19). For each DBD family, the number of domain sites (chromosomal sequences coding for the domain) is presented as well as the positions overlapping with the HRC3 motif. Only families with at least 10 unambiguous domain sites have been considered. To assess the significance of the testing, we employed a Monte-Carlo approach, moving all sequences coding for DNA-binding domains to random positions within the coding part of the genome. Each DBD family was thus shuffled 100 times and the number of domains overlapping with the HRC3 data was computed using *bedtools*.

The columns of the table represent as follows: DBD class; number of domain-coding sequences in the exome (according to *InterPro* database); number of sites overlapping with a HRC3 signature; the ratio of HRC3 overlap to all Domain sites; the fold enrichment of observed overlaps to mean simulated overlaps (HRC3 Overlap / SIM Mean); the minimal, median, mean and maximum number of overlaps present in the simulations.

With 100 simulated distributions, HRC_Overlap greater than SIM_Max implicates a significant overlap, corresponding to $p < 0.01$, or to $p < 0.05$ after Bonferroni correction taking into account that five DBD families have been considered other than homeobox.

TFBS	Binding Sites	HRC3 Overlap	HRC3 Ratio	SIM Fold	SIM Min	SIM 1stQu	SIM Median	SIM Mean	SIM 3rdQu	SIM Max
EZH2	14818	2028	0.1368	6.0864	283	319	333.5	333.2	346.5	388
RBBP5	19205	2121	0.1104	5.9445	299	341.8	358.5	356.8	372.2	406
SUZ12	5772	598	0.1036	8.0236	49	66.75	74	74.53	78.5	110
SAP30	8399	794	0.0945	6.7345	84	108	118.5	117.9	126.2	157
HDAC1	10390	945	0.0909	6.2582	121	142	149	151	160	198
PHF8	17247	1494	0.0866	5.8087	212	244.8	256.5	257.2	268.8	308
UBTF	13613	1131	0.0830	6.8173	132	155	166.5	165.9	177	206
HMGN3	13061	1034	0.0791	5.7797	137	170	177.5	178.9	188.2	217
E2F1	17997	1392	0.0773	5	219	264	278	278.4	293.2	327
KDM5B	12943	963	0.0744	5.3233	139	173	180	180.9	192.2	217
CHD1	16981	1254	0.0738	5.0240	206	235.8	247	249.6	261.5	305
SMARCB1	8485	625	0.0736	4.4770	97	130	141	139.6	147.2	184
HDAC6	1110	81	0.0729	8.8621	2	7	9	9.14	11.25	18
SP4	5352	382	0.0713	4.4444	59	78.75	85	85.95	94	117
CTBP2	6537	459	0.0702	4.6181	63	91.5	99	99.39	108.2	143
RDBP	440	30	0.0681	5.5147	0	3	5	5.44	8	13
SIN3A	23062	1536	0.0661	4.5768	295	321	336	335.6	348	393
E2F6	25551	1549	0.0606	5.6948	214	260.2	273	272	283.2	320
ZNF263	27758	1662	0.0598	4.2746	336	373.8	387	388.8	401.2	439
CCNT2	18076	1079	0.0596	5.0046	177	204	215.5	215.6	226.2	257
KDM5A	1580	94	0.0594	6.5826	5	11	14	14.28	17.25	26
TAF7	11580	653	0.0563	5.8565	78	101.8	110	111.5	120.2	140
ZEB1	4807	266	0.0553	6.2869	23	37.75	42	42.31	47.25	60
ZNF274	1858	100	0.0538	2.4172	24	36	41	41.37	47	64
TAF1	40355	2136	0.0529	4.3806	414	466	487	487.6	514	552
POLR2A	134990	6865	0.0508	3.1621	1992	2136	2166	2171	2208	2338
THAP1	3469	175	0.0504	6.0096	15	25.75	29	29.12	32	50
ETS1	13737	679	0.0494	4.3863	119	143	155	154.8	166.2	189
HDAC8	1607	77	0.0479	3.3757	9	19.75	23	22.81	26	44
SIN3AK20	37414	1740	0.0465	3.8024	388	443	456.5	457.6	472	538
GABPA	26985	1243	0.0460	4.2862	251	274	287	290	307	341
NRF1	7864	362	0.0460	5.4732	45	59	65.5	66.14	72	95
ELK4	5860	261	0.0445	3.4555	52	67	76	75.53	82	102
MXI1	36263	1445	0.0398	3.3730	368	410	426	428.4	448.2	490
MAZ	43914	1731	0.0394	3.3455	448	499	516	517.4	538.2	591
PML	23278	906	0.0389	2.7808	259	309	323.5	325.8	344.2	383
ELK1	8455	323	0.0382	3.5611	60	82	90	90.7	99.25	119
SMARCC1	7650	280	0.036601307	2.2526	86	115	126	124.3	133.2	155
TCF3	15917	582	0.0365	4.6190	93	117	125	126	136	155
GTF2F1	14768	522	0.0353	3.0034	130	163	170	173.8	183	230

ZBTB7A	25063	879	0.0350	3.6686	197	228	238.5	239.6	254.2	290
FOXP2	27652	947	0.0342	3.9856	172	226	237	237.6	249	283
MAX	85949	2923	0.0340	3.3227	795	856	879.5	879.7	902.2	955
IRF1	20215	680	0.0336	3.2380	154	197.8	209	210	220	254
BCLAF1	8943	298	0.0333	3.0996	67	85	95.5	96.14	105	128
E2F4	18683	620	0.033185 249	3.5590	146	164	173	174.2	183	222
TBP	45699	1514	0.0331	3.0961	399	471.5	490	489	513	555
YY1	64138	2124	0.0331	3.3146	562	620.8	643	640.8	660.2	692
REST	58014	1909	0.032905 85	3.5290	470	520	542	540.9	558.2	603
GTF2B	2844	93	0.0327	3.4016	12	23	27	27.34	32	46
CREB1	15809	516	0.0326	3.8165	98	125.8	137	135.2	144	172
GRp20	590	19	0.0322	3.3391	0	4	6	5.69	8	11
SP2	5441	175	0.03216	3.5612	28	43	49	49.14	56	70
TFAP2A	16808	534	0.0317	2.4428	174	207	219	218.6	230	276
CTCFL	11533	366	0.03173	5.3438	46	60	68	68.49	76.25	98
ELF1	44120	1373	0.031119 674	3.3784	344	389.2	409	406.4	424	452
NR2C2	4601	143	0.0310	3.0023	30	43	48	47.63	53	71
MYC	96959	2985	0.03078	3.0142	904	966.8	987	990.3	1013	1105
TFAP2C	22481	690	0.0306	2.4083	239	270.8	285	286.5	303.2	332
SREBP1	2546	77	0.0302	2.8174	13	22	27	27.33	31	45
ZBTB33	12769	383	0.0299	2.8862	102	125	132	132.7	140	168
CHD2	33645	1009	0.0299	2.8721	306	336	347.5	351.3	365.2	405
SMARCC2	2543	76	0.0298	2.2099	20	29.75	34	34.39	40	52
CTCF	162209	4733	0.0291	3.1590	1385	1462	1496	1498	1535	1594
PPARGC1A	1291	36	0.0278	2.6335	5	10	13	13.67	16.25	28
CBX3	20197	549	0.0271	2.4796	181	206	222	221.4	234	278
EGR1	44985	1212	0.0269	3.6440	276	317.8	333	332.6	346.2	393
BACH1	13283	357	0.0268	3.0722	86	105.8	116.5	116.2	127	147
ZKSCAN1	4066	108	0.0265	2.7033	19	35	40	39.95	44	61
PAX5	33070	876	0.0264	3.1831	231	261	275	275.2	288.2	321
MTA3	11136	293	0.0263	1.9150	117	143	151.5	153	162.2	193
MYBL2	16138	417	0.0258	1.8533	185	210.8	224.5	225	236.2	279
ESRRA	1174	30	0.0255	2.58843	3	10	12	11.59	14	22
SMARCA4	3431	86	0.0250	1.5501	38	49	55	55.48	61	96
SIX5	7911	194	0.0245	2.398	57	73	80.5	80.9	88	111
NFYA	7335	179	0.0244	2.2767	56	71.75	79	78.62	85.25	98
TCF12	43585	1058	0.0242	2.5317	360	403	418	417.9	430.5	481
STAT1	19147	457	0.0238	2.0791	166	207.8	221	219.8	232	271
HDAC2	31954	761	0.023815 485	2.765261 628	229	262	274	275.2	284	321
KAP1	26393	627	0.023756 299	1.386554 622	373	438	450	452.2	470.2	517

FOXM1	21667	509	0.0234	2.0499	199	235	249	248.3	262	288
GATA1	20310	473	0.0232	1.6660	233	270	284	283.9	295.2	332
TCF7L2	45100	1050	0.0232	1.8656	493	544.8	561	562.8	584.2	637
IRF3	1722	40	0.0232	2.3668	5	13	17	16.9	19	32
POU2F2	26413	613	0.0232	2.636	192	221	232	232.5	242	278
RELA	36928	853	0.0230	2.1293	346	383.5	400.5	400.6	420.2	459
ZNF143	44997	1032	0.0229	2.7279	313	364.8	379	378.3	397.2	442
BHLHE40	38801	878	0.0226	2.5907	294	323.8	337.5	338.9	351.2	416
BRCA1	9245	207	0.0224	2.6272	50	72	79	78.79	85.25	111
TBL1XR1	22156	494	0.0222	2.1066	177	219	235.5	234.5	250	278
ATF2	26026	576	0.0221	1.9129	250	291	302	301.1	312.2	364
WRNIP1	13542	297	0.0219	2.0944	100	129.8	143	141.8	151	175
CEBPD	11399	250	0.0219	2.7839	67	82	88.5	89.8	97.25	122
STAT5A	16120	343	0.0212	2.0152	122	160	168	170.2	182	204
NFATC1	10183	211	0.0207	1.5769	94	123	132.5	133.8	143	183
RCOR1	49688	1015	0.0204	2.1940	402	444	457.5	462.6	481.8	530
MBD4	5549	113	0.0203	1.5771	53	64	71	71.65	78	104
SETDB1	22192	431	0.0194	1.2463	278	329.8	344.5	345.8	359.8	422
NR3C1	35176	677	0.0192	2.3082	240	276.8	289	293.3	314.2	346
BCL3	22617	424	0.018	1.7950	195	222.8	236	236.2	246.5	286
ATF3	23095	429	0.0185	2.1721	162	186.8	196.5	197.5	206	247
NFIC	38851	712	0.0183	1.5194	387	451.8	470	468.6	488.8	539
SRF	16014	287	0.0179	2.7281	78	96	105	105.2	114	138
SMC3	56772	991	0.0174	2.303	363	418	432.5	430.2	444.2	482
RAD21	119039	2048	0.0172	2.5073	712	796.5	817	816.8	838	928
TRIM28	11915	204	0.0171	1.6721	92	113	123	122	130	152
RFX5	24892	415	0.0166	1.8146	172	216	229	228.7	242	287
SP1	49005	813	0.0165	1.8849	380	417.8	434	431.3	443	503
PBX3	9927	164	0.016	2.457	45	60	68	66.74	73	91
NR2F2	16296	265	0.0162	1.6793	107	149	157.5	157.8	165	200
ZNF217	9204	149	0.01618	1.2064	97	114	122	123.5	132.2	162
RUNX3	66222	1051	0.0158	1.9681	473	518.8	536	534	549.2	602
USF2	23709	371	0.0156	1.945	152	180	190	190.7	201	238
IKZF1	7726	120	0.015	1.084	73	101	111	110.7	119.2	134
EBF1	48897	754	0.0154	1.9210	335	378.8	393.5	392.5	406.5	443
MEF2C	9210	140	0.0152	2.072	46	62	67	67.56	72.25	95
STAT2	3936	58	0.0147	1.5855	22	32	36	36.58	39.25	56
NFYB	17509	257	0.0146	1.5099	127	159	170	170.2	181	207
USF1	58534	843	0.0144	1.9348	374	421.5	437	435.7	449.5	492
FOSL1	11937	166	0.0139	2.0804	60	73	78	79.79	86	102
JUNB	12142	166	0.0136	1.4409	85	105.8	113.5	115.2	124	169

JUND	97411	1311	0.0134	1.5614	764	815.8	838	839.6	863	928
TEAD4	58029	779	0.0134	1.5930	430	471.8	489.5	489	508.2	559
NANOG	5473	73	0.0133	2.0154	18	30	37	36.22	42	53
POLR3G	230	3	0.0130	0.8823	0	2	3	3.4	5	11
IRF4	17722	225	0.0126	1.6210	110	130	138	138.8	147.2	173
GATA2	73974	891	0.0120	1.2177	664	712.8	728.5	731.7	753	796
EP300	138836	1648	0.0118	1.3508	1087	1194	1221	1220	1242	1318
HSF1	1434	17	0.0118	1.4592	3	10	12	11.65	14	19
ZZZ3	852	10	0.0117	1.2345	2	6	7.5	8.1	10	19
HNF4A	22374	259	0.0115	1.5031	136	161.8	172	172.3	183	211
RXRA	19229	214	0.0111	1.4748	110	136	144	145.1	154	191
ESR1	25776	279	0.0108	1.3616	169	192.8	202	204.9	216.2	248
SPI1	67572	731	0.0108	1.6087	395	436.8	456	454.4	474.2	501
ATF1	14850	155	0.0100	1.4608	76	97.75	104.5	106.1	114	143
BRF1	291	3	0.0103	1.2605	0	1	2	2.38	3	9
GATA3	55528	559	0.0100	1.0774	467	503.8	519	518.8	532.5	590
SIRT6	2291	23	0.0100	1.0531	11	18	22	21.84	25	38
MEF2A	21875	219	0.0100	1.4239	125	145	154.5	153.8	164	183
HNF4G	20339	200	0.0098	1.2987	112	139	156	154	165	208
ARID3A	24307	237	0.0097	1.0206	180	220.8	233	232.2	245	299
TAL1	26211	255	0.0097	1.3571	153	178.8	187.5	187.9	197	230
FOSL2	43464	418	0.0096	1.142	320	346	362	366	384	436
BRF2	1367	13	0.0095	1.248	1	8	10	10.41	13	18
POU5F1	3997	38	0.0095	1.5859	11	20	23	23.96	28	37
GTF3C2	3812	36	0.0094	1.0471	17	30	34	34.38	39	49
BCL11A	20286	184	0.0090	1.3489	95	126.8	138	136.4	145	168
JUN	58594	508	0.0080	1.080	411	451	469.5	470.1	487	555
STAT3	67977	572	0.0084	1.0284	493	535.8	557	556.2	573.2	641
NFE2	3169	26	0.0082	1.267	7	18	20	20.52	23	39
CEBPB	146873	1190	0.0081	1.1152	943	1038	1070	1067	1091	1166
FOS	131528	1047	0.0079	1.0335	918	992.8	1012	1013	1032	1114
BATF	32419	239	0.0073	1.2513	154	180	191.5	191	201.2	234
MAFF	47076	346	0.0073	1.0282	285	320	336	336.5	351.2	401
MAFK	84087	618	0.0073	1.0363	540	578	596.5	596.3	611	650
RPC155	2806	19	0.0067	0.5521	21	31	34	34.41	38.25	54
FOXA1	89906	604	0.0067	0.9596	532	607	630	629.4	648.2	707
BDP1	791	5	0.0063	0.8605	0	4	5.5	5.81	8	13
PRDM1	4574	26	0.0056	0.8373	15	27.75	31	31.05	34	47
FOXA2	40866	227	0.0055	0.8156	225	268.8	277	278.3	288.2	323
FAM48A	4087	16	0.0039	0.644	13	20	24	24.82	29	43

Table S7. Transcription Factor Binding Sites (TFBS) from the ENCODE project with peaks overlapping HRC3 in human genome version hg19. For each TFBS the total number of peaks is represented as well as peaks overlapping with HRC3. The HRC3 Ratio is the proportion of HRC3 peaks present in the TFBS Chip-seq data. To compute the significance of the testing, each TFBS was shuffled 100 times and the number of peaks overlapping with the HRC3 data was computed using bedtools. The median number of overlapping peaks (SIM Median column) then represents the number of overlapping due to random effect. Typically, statistically significant overlapping peaks will have their SIM Median values inferior their HRC3 Ratio.

Supplementary Figure File SF1. The periodograms of the HRC pattern in the homeoboxes of human and mouse HOX genes. Each panel corresponds to one gene. The periodograms of homeobox are shown for mouse (red) and human (blue), as well as for an adjacent region 5’ of the homeobox in mouse (green). Dotted line: p=0.05 significance threshold for a single frequency.

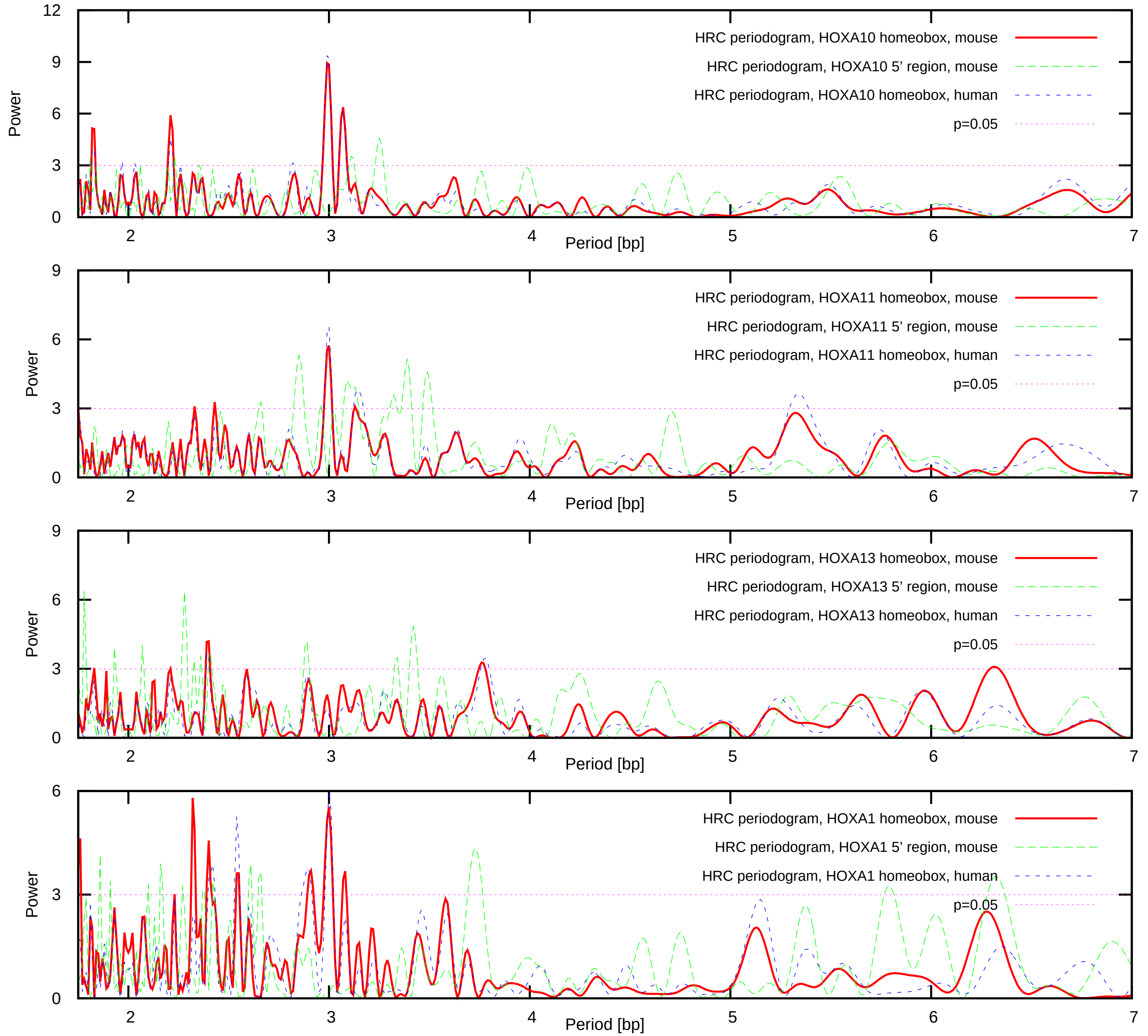
Power
Period [bp]
HRC periodogram, HOXA10 homeobox, mouse
HRC periodogram, HOXA10 5' region, mouse
HRC periodogram, HOXA10 homeobox, human
p=0.05
HRC periodogram, HOXA11 homeobox, mouse
HRC periodogram, HOXA11 5' region, mouse
HRC periodogram, HOXA11 homeobox, human
HRC periodogram, HOXA13 homeobox, mouse
HRC periodogram, HOXA13 5' region, mouse
HRC periodogram, HOXA13 homeobox, human
HRC periodogram, HOXA1 homeobox, mouse
HRC periodogram, HOXA1 5' region, mouse
HRC periodogram, HOXA1 homeobox, human

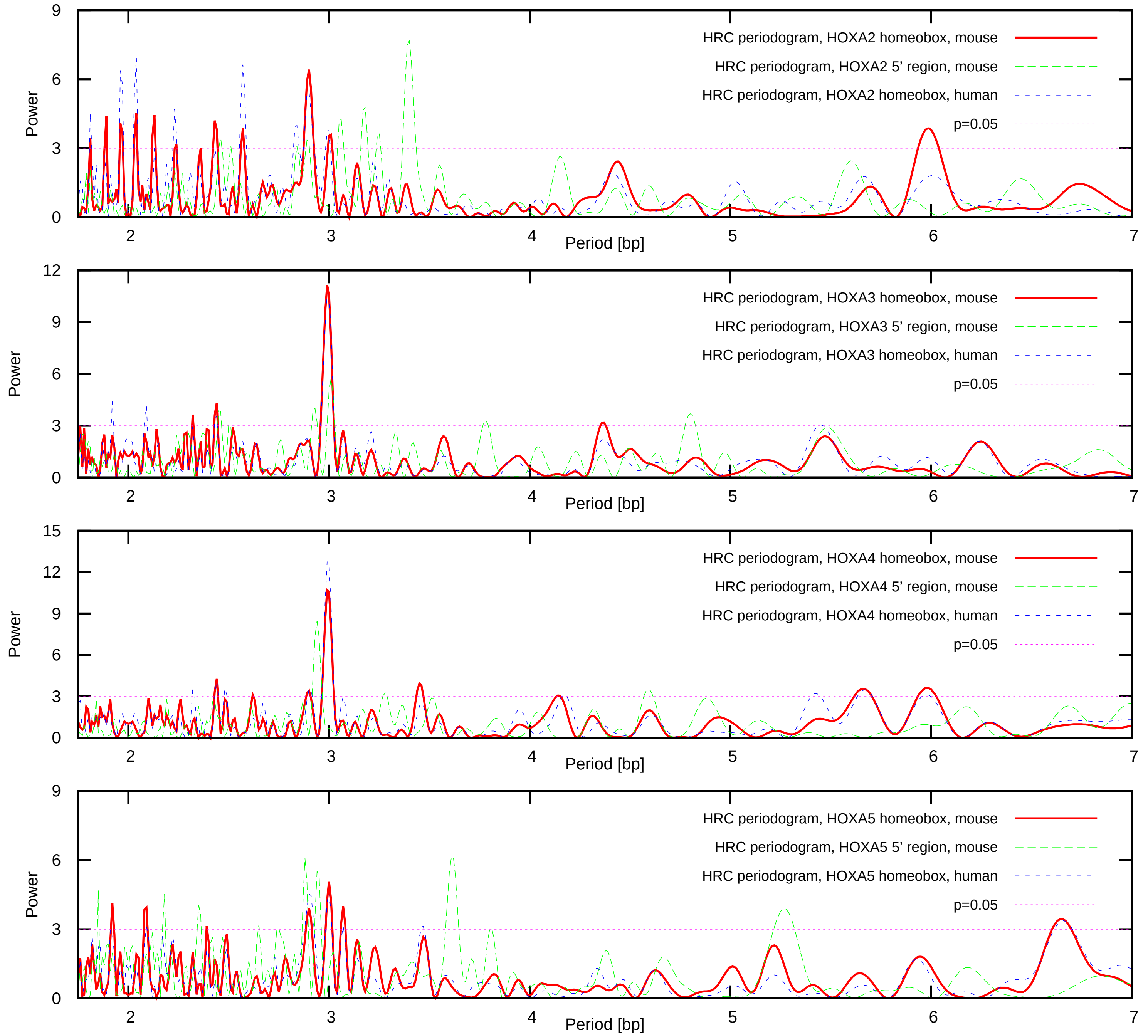
HRC periodogram, HOXA2 homeobox, mouse
HRC periodogram, HOXA2 5' region, mouse
HRC periodogram, HOXA2 homeobox, human
p=0.05
Power
Period [bp]
HRC periodogram, HOXA3 homeobox, mouse
HRC periodogram, HOXA3 5' region, mouse
HRC periodogram, HOXA3 homeobox, human
p=0.05
Power
Period [bp]
HRC periodogram, HOXA4 homeobox, mouse
HRC periodogram, HOXA4 5' region, mouse
HRC periodogram, HOXA4 homeobox, human
p=0.05
Power
Period [bp]
HRC periodogram, HOXA5 homeobox, mouse
HRC periodogram, HOXA5 5' region, mouse
HRC periodogram, HOXA5 homeobox, human
p=0.05
Power
Period [bp]

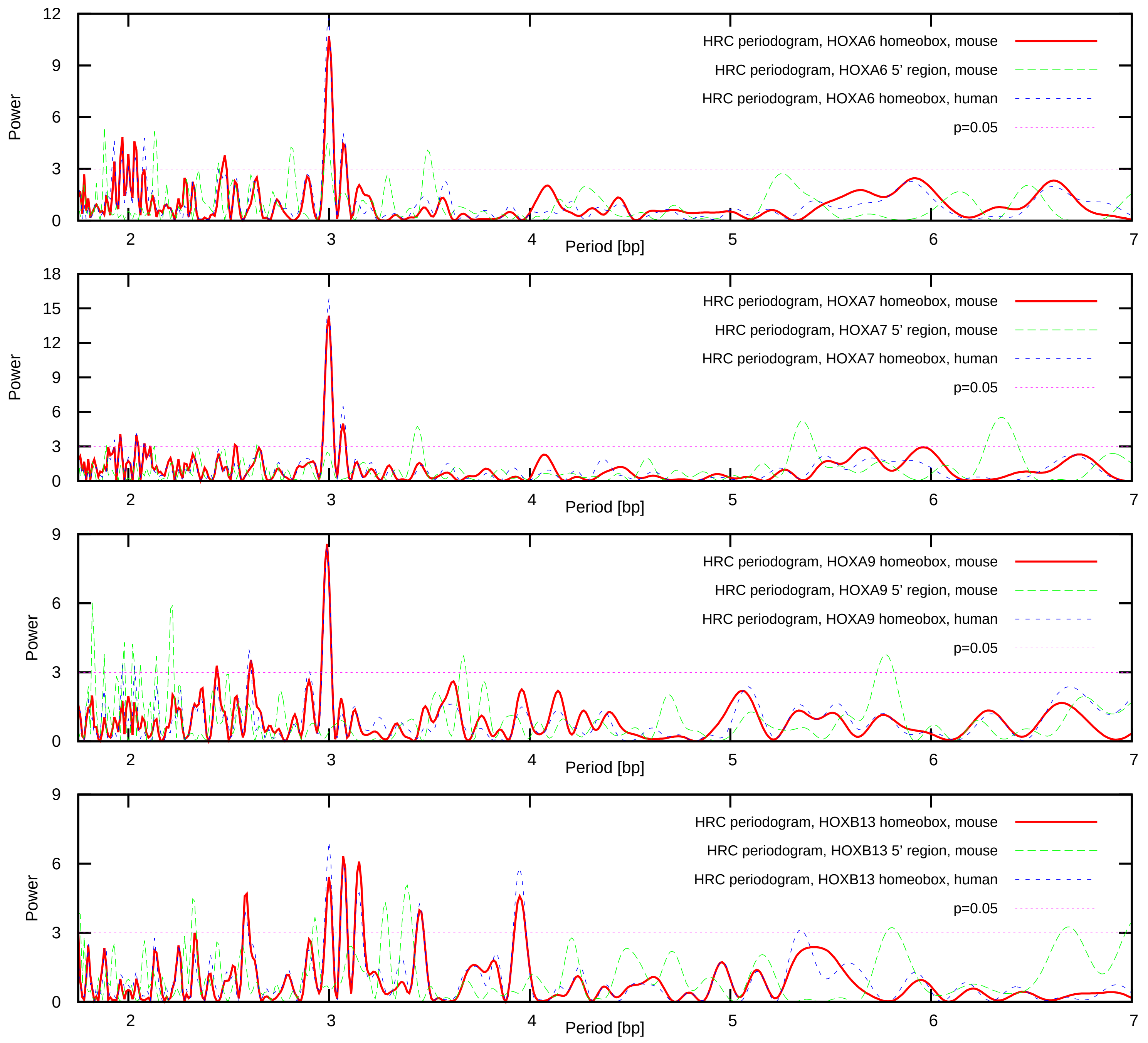

HRC periodogram, HOXA6 homeobox, mouse
HRC periodogram, HOXA6 5' region, mouse
HRC periodogram, HOXA6 homeobox, human
p=0.05
Power
Period [bp]
HRC periodogram, HOXA7 homeobox, mouse
HRC periodogram, HOXA7 5' region, mouse
HRC periodogram, HOXA7 homeobox, human
p=0.05
Power
Period [bp]
HRC periodogram, HOXA9 homeobox, mouse
HRC periodogram, HOXA9 5' region, mouse
HRC periodogram, HOXA9 homeobox, human
p=0.05
Power
Period [bp]
HRC periodogram, HOXB13 homeobox, mouse
HRC periodogram, HOXB13 5' region, mouse
HRC periodogram, HOXB13 homeobox, human
p=0.05
Power
Period [bp]

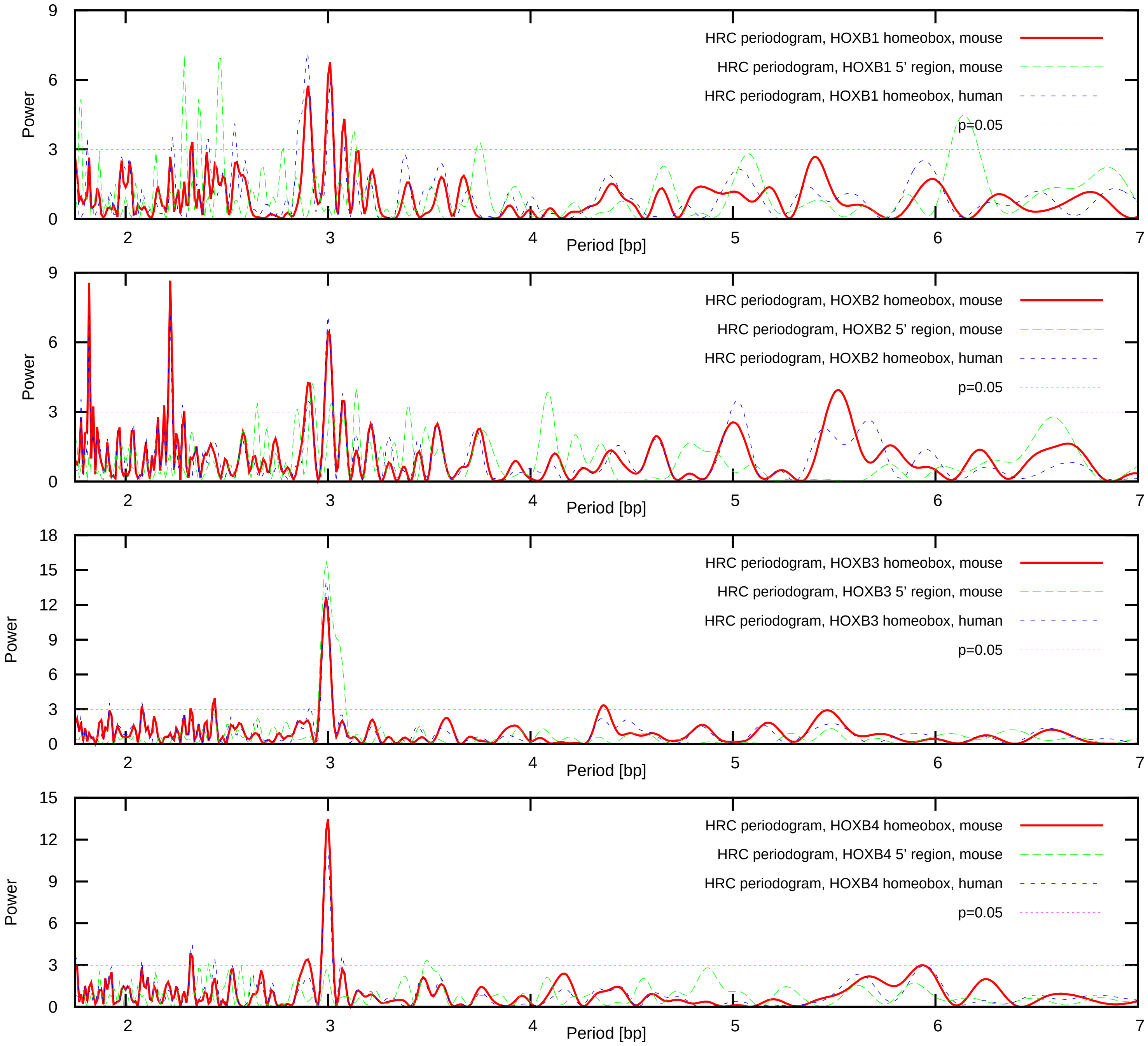

HRC periodogram, HOXB1 homeobox, mouse
HRC periodogram, HOXB1 5' region, mouse
HRC periodogram, HOXB1 homeobox, human
p=0.05
Power
Period [bp]
HRC periodogram, HOXB2 homeobox, mouse
HRC periodogram, HOXB2 5' region, mouse
HRC periodogram, HOXB2 homeobox, human
p=0.05
Power
Period [bp]
HRC periodogram, HOXB3 homeobox, mouse
HRC periodogram, HOXB3 5' region, mouse
HRC periodogram, HOXB3 homeobox, human
p=0.05
Power
Period [bp]
HRC periodogram, HOXB4 homeobox, mouse
HRC periodogram, HOXB4 5' region, mouse
HRC periodogram, HOXB4 homeobox, human
p=0.05
Power
Period [bp]

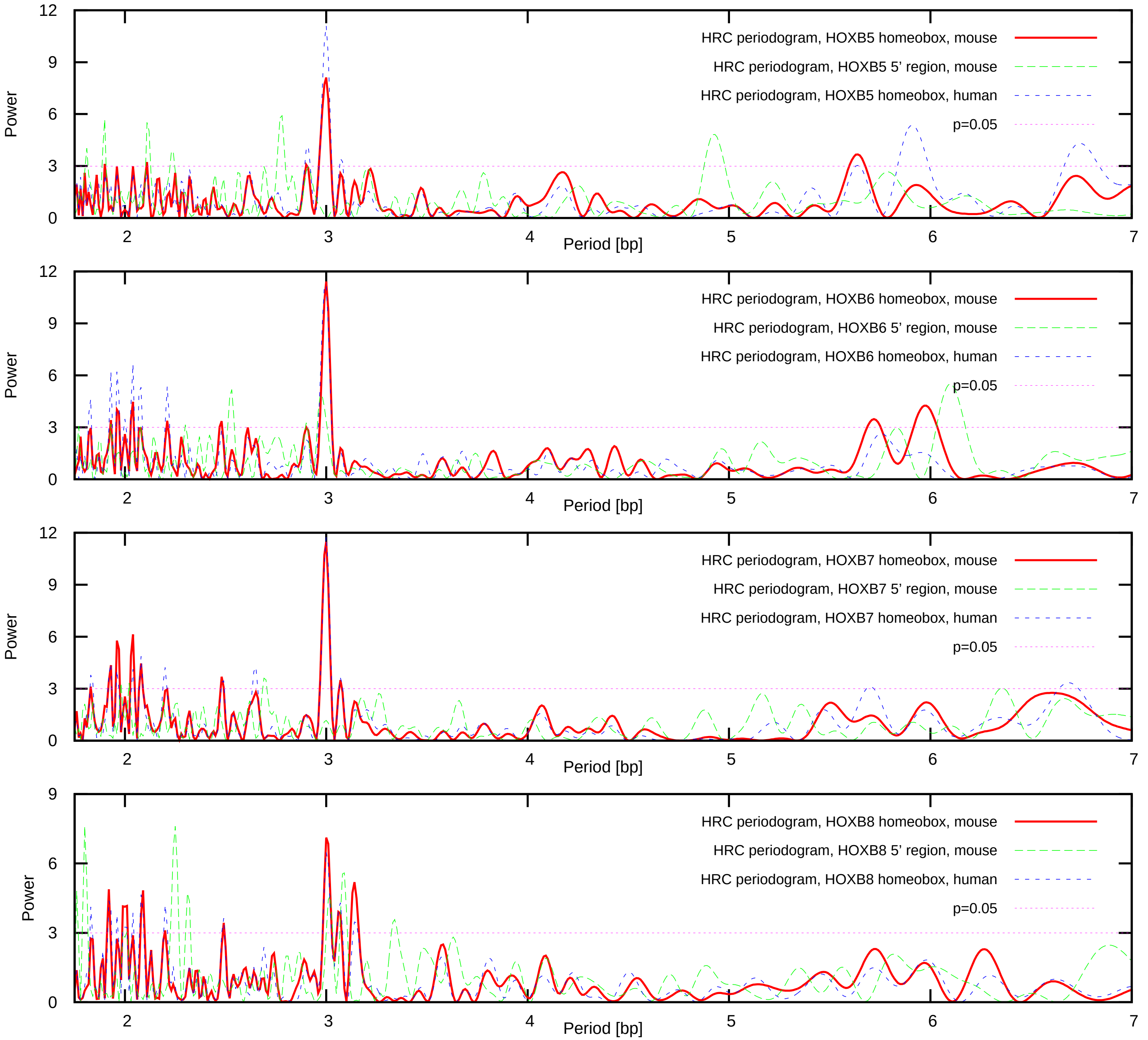

HRC periodogram, HOXB5 homeobox, mouse
HRC periodogram, HOXB5 5' region, mouse
HRC periodogram, HOXB5 homeobox, human
p=0.05
HRC periodogram, HOXB6 homeobox, mouse
HRC periodogram, HOXB6 5' region, mouse
HRC periodogram, HOXB6 homeobox, human
p=0.05
HRC periodogram, HOXB7 homeobox, mouse
HRC periodogram, HOXB7 5' region, mouse
HRC periodogram, HOXB7 homeobox, human
p=0.05
HRC periodogram, HOXB8 homeobox, mouse
HRC periodogram, HOXB8 5' region, mouse
HRC periodogram, HOXB8 homeobox, human
p=0.05
Power
Period [bp]
0
3
6
9
12
2
3
4
5
6
7

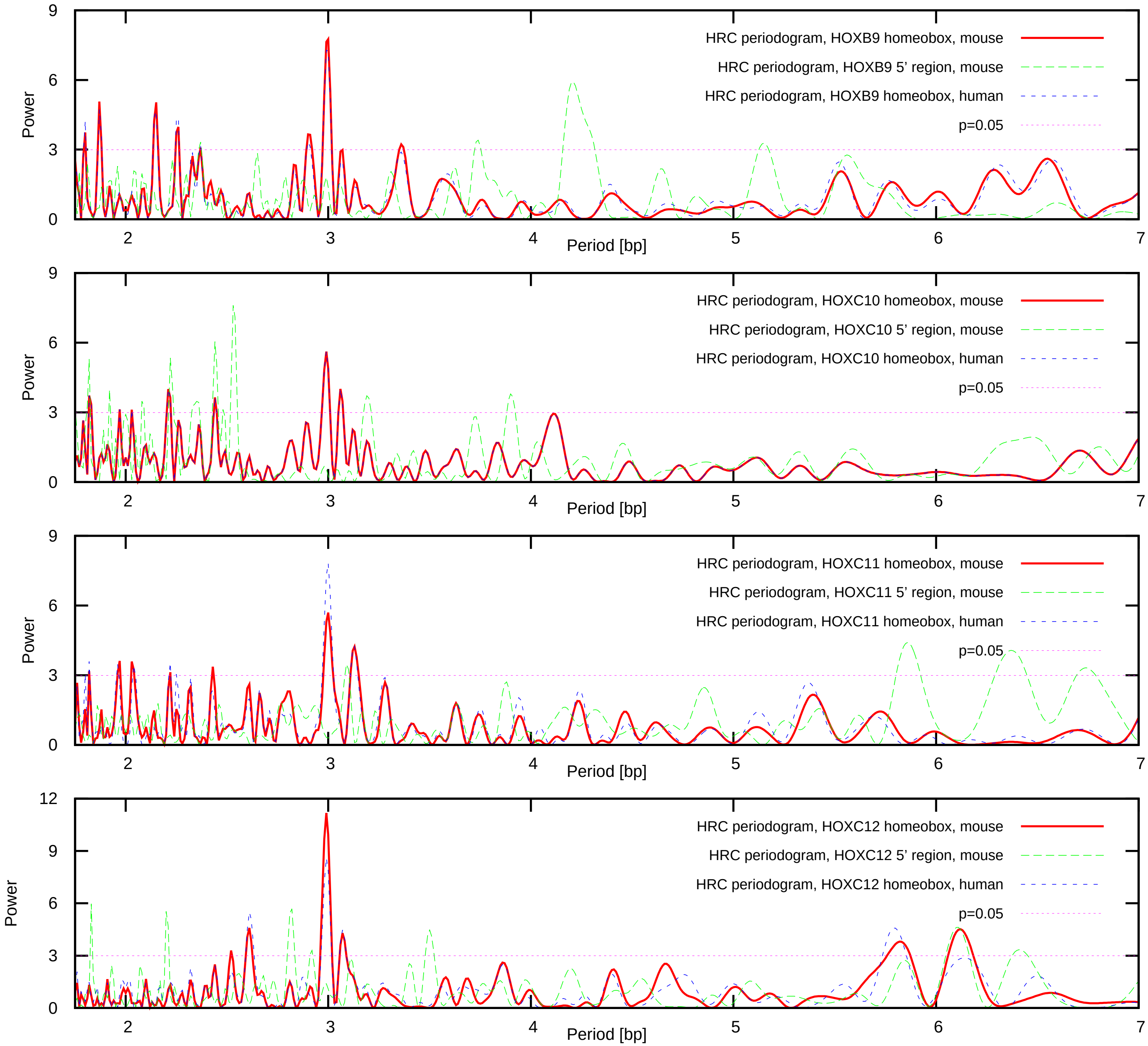

HRC periodogram, HOXB9 homeobox, mouse
HRC periodogram, HOXB9 5' region, mouse
HRC periodogram, HOXB9 homeobox, human
p=0.05
Power
Period [bp]
HRC periodogram, HOXC10 homeobox, mouse
HRC periodogram, HOXC10 5' region, mouse
HRC periodogram, HOXC10 homeobox, human
p=0.05
Power
Period [bp]
HRC periodogram, HOXC11 homeobox, mouse
HRC periodogram, HOXC11 5' region, mouse
HRC periodogram, HOXC11 homeobox, human
p=0.05
Power
Period [bp]
HRC periodogram, HOXC12 homeobox, mouse
HRC periodogram, HOXC12 5' region, mouse
HRC periodogram, HOXC12 homeobox, human
p=0.05
Power
Period [bp]

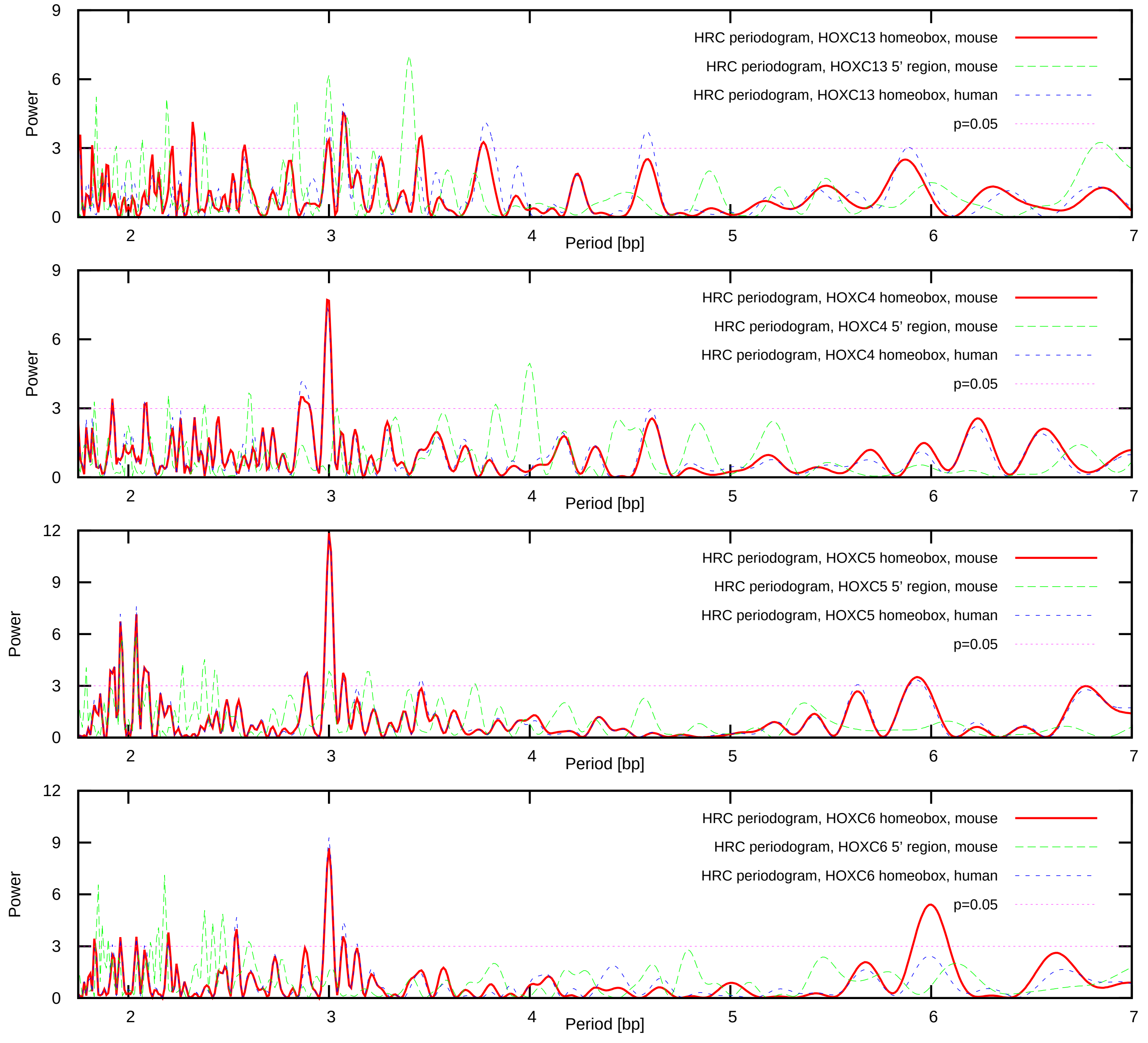

HRC periodogram, HOXC13 homeobox, mouse
HRC periodogram, HOXC13 5' region, mouse
HRC periodogram, HOXC13 homeobox, human
p=0.05
Power
Period [bp]
HRC periodogram, HOXC4 homeobox, mouse
HRC periodogram, HOXC4 5' region, mouse
HRC periodogram, HOXC4 homeobox, human
p=0.05
Power
Period [bp]
HRC periodogram, HOXC5 homeobox, mouse
HRC periodogram, HOXC5 5' region, mouse
HRC periodogram, HOXC5 homeobox, human
p=0.05
Power
Period [bp]
HRC periodogram, HOXC6 homeobox, mouse
HRC periodogram, HOXC6 5' region, mouse
HRC periodogram, HOXC6 homeobox, human
p=0.05
Power
Period [bp]

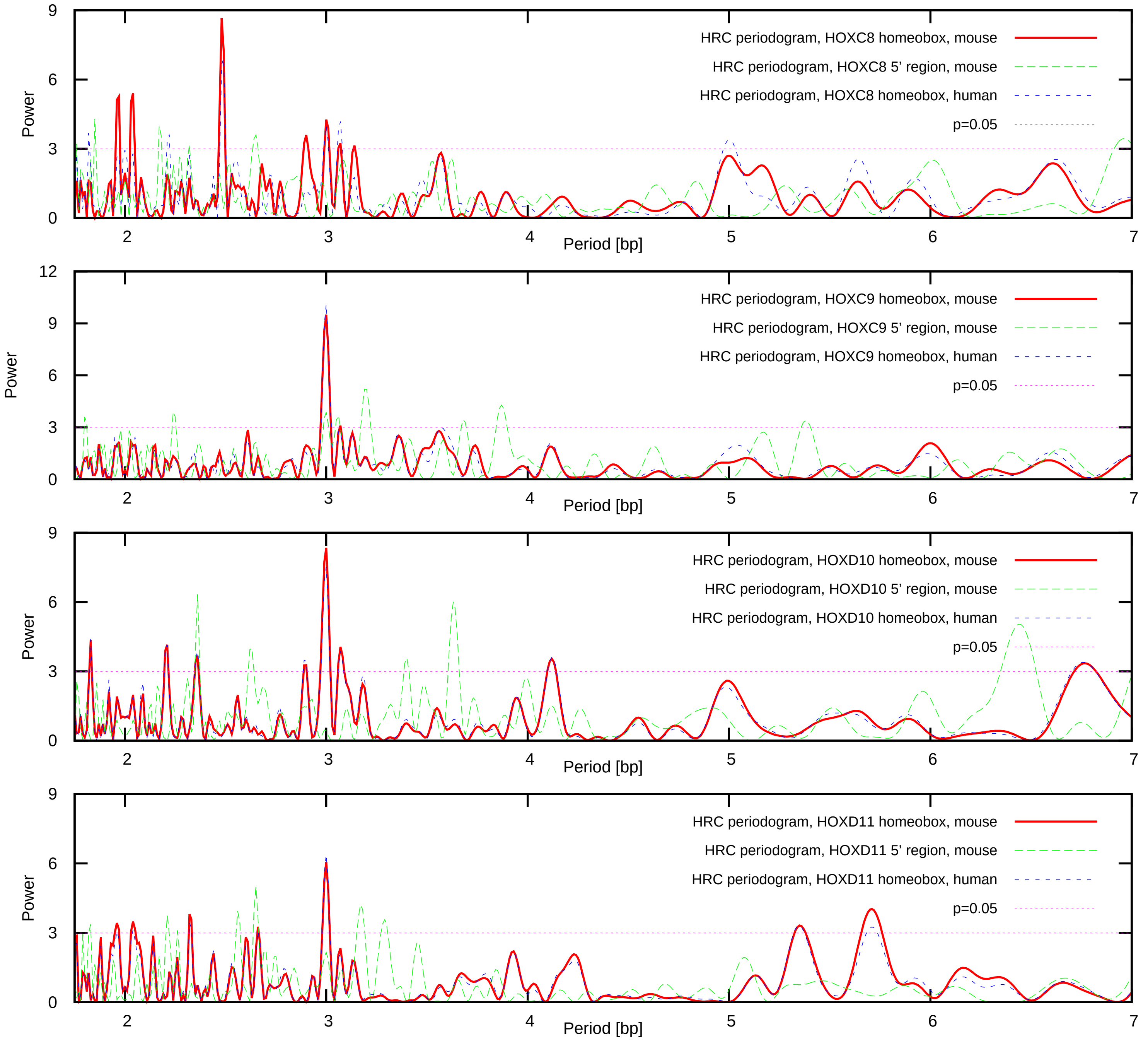

Power
Period [bp]
HRC periodogram, HOXC8 homeobox, mouse
HRC periodogram, HOXC8 5' region, mouse
HRC periodogram, HOXC8 homeobox, human
p=0.05
HRC periodogram, HOXC9 homeobox, mouse
HRC periodogram, HOXC9 5' region, mouse
HRC periodogram, HOXC9 homeobox, human
HRC periodogram, HOXD10 homeobox, mouse
HRC periodogram, HOXD10 5' region, mouse
HRC periodogram, HOXD10 homeobox, human
HRC periodogram, HOXD11 homeobox, mouse
HRC periodogram, HOXD11 5' region, mouse
HRC periodogram, HOXD11 homeobox, human
0
3
6
9
12
2
3
4
5
6
7

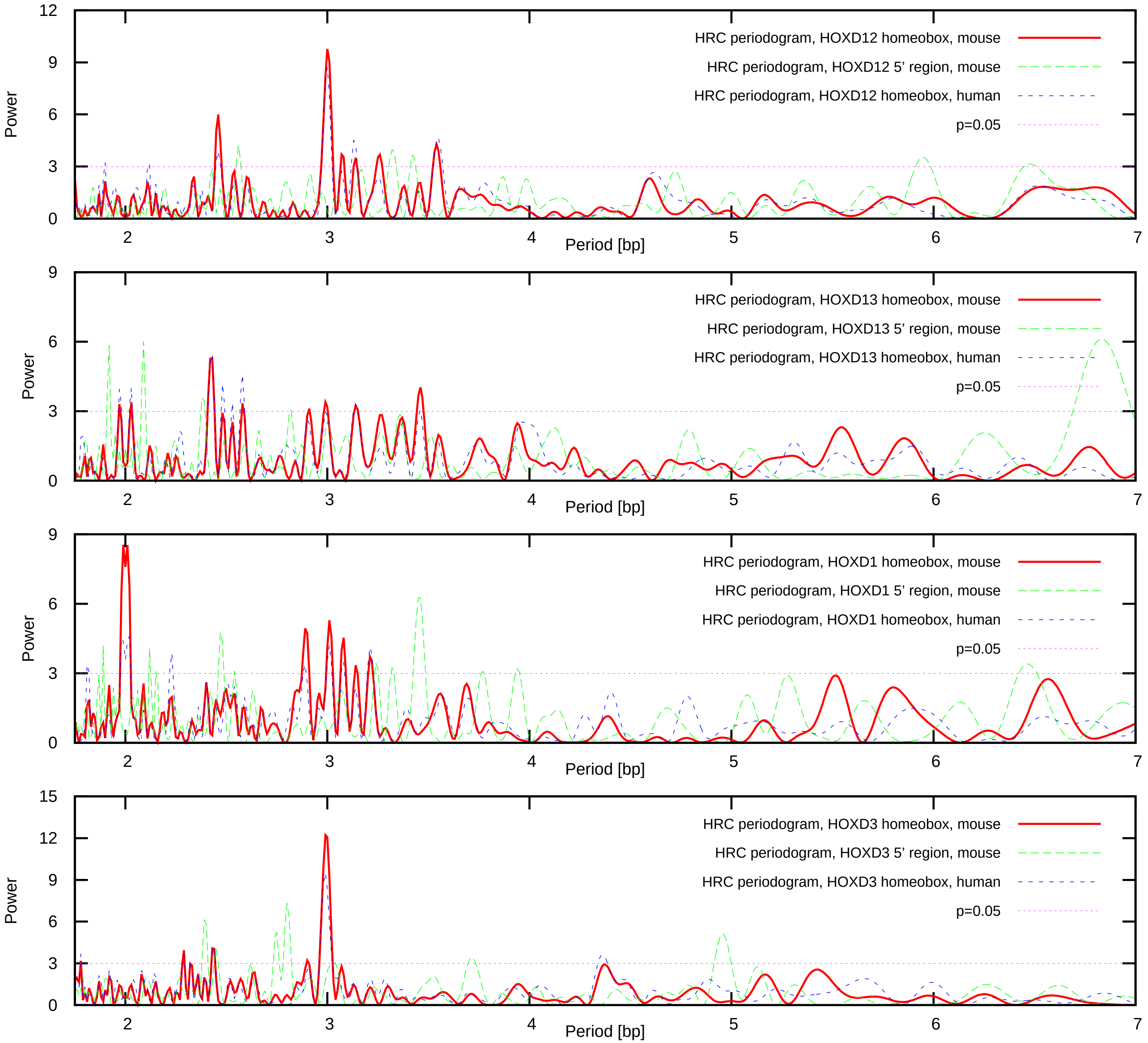
HRC periodogram, HOXD12 homeobox, mouse
HRC periodogram, HOXD12 5' region, mouse
HRC periodogram, HOXD12 homeobox, human
p=0.05
Power
Period [bp]
HRC periodogram, HOXD13 homeobox, mouse
HRC periodogram, HOXD13 5' region, mouse
HRC periodogram, HOXD13 homeobox, human
p=0.05
Power
Period [bp]
HRC periodogram, HOXD1 homeobox, mouse
HRC periodogram, HOXD1 5' region, mouse
HRC periodogram, HOXD1 homeobox, human
p=0.05
Power
Period [bp]
HRC periodogram, HOXD3 homeobox, mouse
HRC periodogram, HOXD3 5' region, mouse
HRC periodogram, HOXD3 homeobox, human
p=0.05
Power
Period [bp]

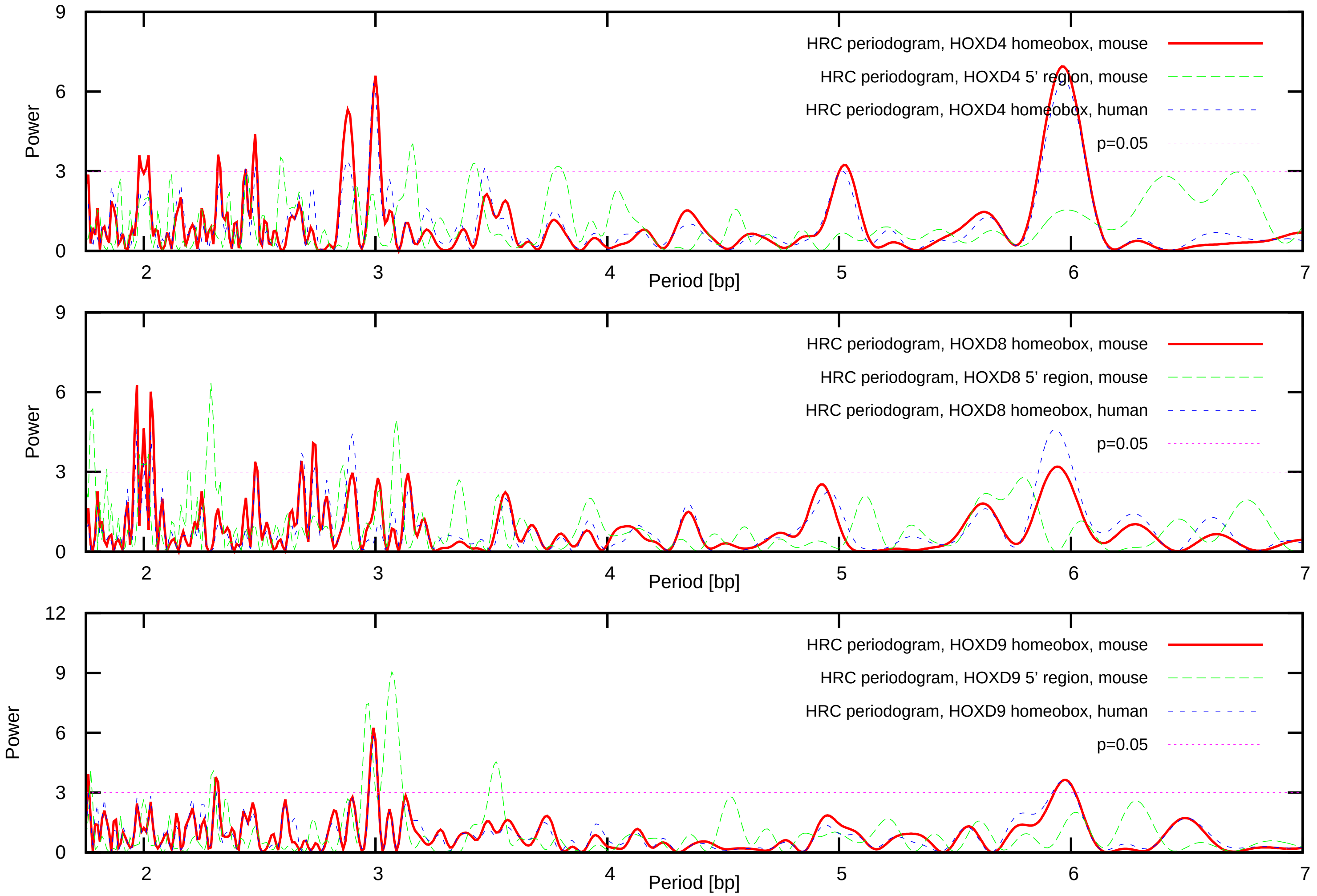
HRC periodogram, HOXD4 homeobox, mouse
HRC periodogram, HOXD4 5' region, mouse
HRC periodogram, HOXD4 homeobox, human
p=0.05
Power
Period [bp]
HRC periodogram, HOXD8 homeobox, mouse
HRC periodogram, HOXD8 5' region, mouse
HRC periodogram, HOXD8 homeobox, human
p=0.05
Power
Period [bp]
HRC periodogram, HOXD9 homeobox, mouse
HRC periodogram, HOXD9 5' region, mouse
HRC periodogram, HOXD9 homeobox, human
p=0.05
Power
Period [bp]

Supplementary Figure File SF2. The periodograms of the HRC pattern in human and mouse homeobox-containing genes outside the HOX clusters. Each panel corresponds to one gene. The periodograms of homeobox are shown for mouse (red) and human (blue), as well as for an adjacent region 5' of the homeobox in mouse (green). Dotted line: p=0.05 significance threshold for a single frequency.

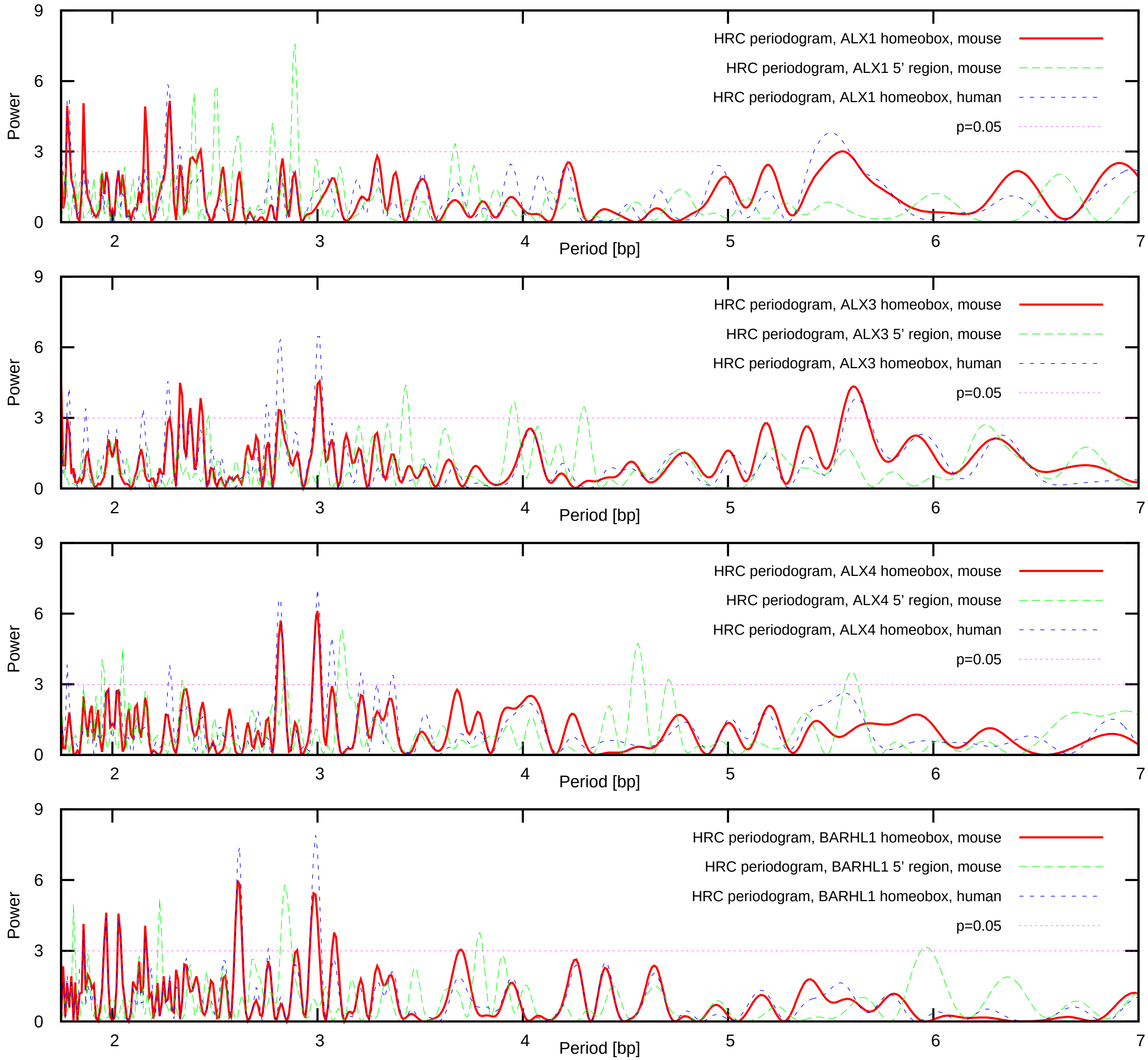

HRC periodogram, ALX1 homeobox, mouse
HRC periodogram, ALX1 5' region, mouse
HRC periodogram, ALX1 homeobox, human
p=0.05
HRC periodogram, ALX3 homeobox, mouse
HRC periodogram, ALX3 5' region, mouse
HRC periodogram, ALX3 homeobox, human
p=0.05
HRC periodogram, ALX4 homeobox, mouse
HRC periodogram, ALX4 5' region, mouse
HRC periodogram, ALX4 homeobox, human
p=0.05
HRC periodogram, BARHL1 homeobox, mouse
HRC periodogram, BARHL1 5' region, mouse
HRC periodogram, BARHL1 homeobox, human
p=0.05
Power
Period [bp]
0
3
6
9
2
3
4
5
6
7

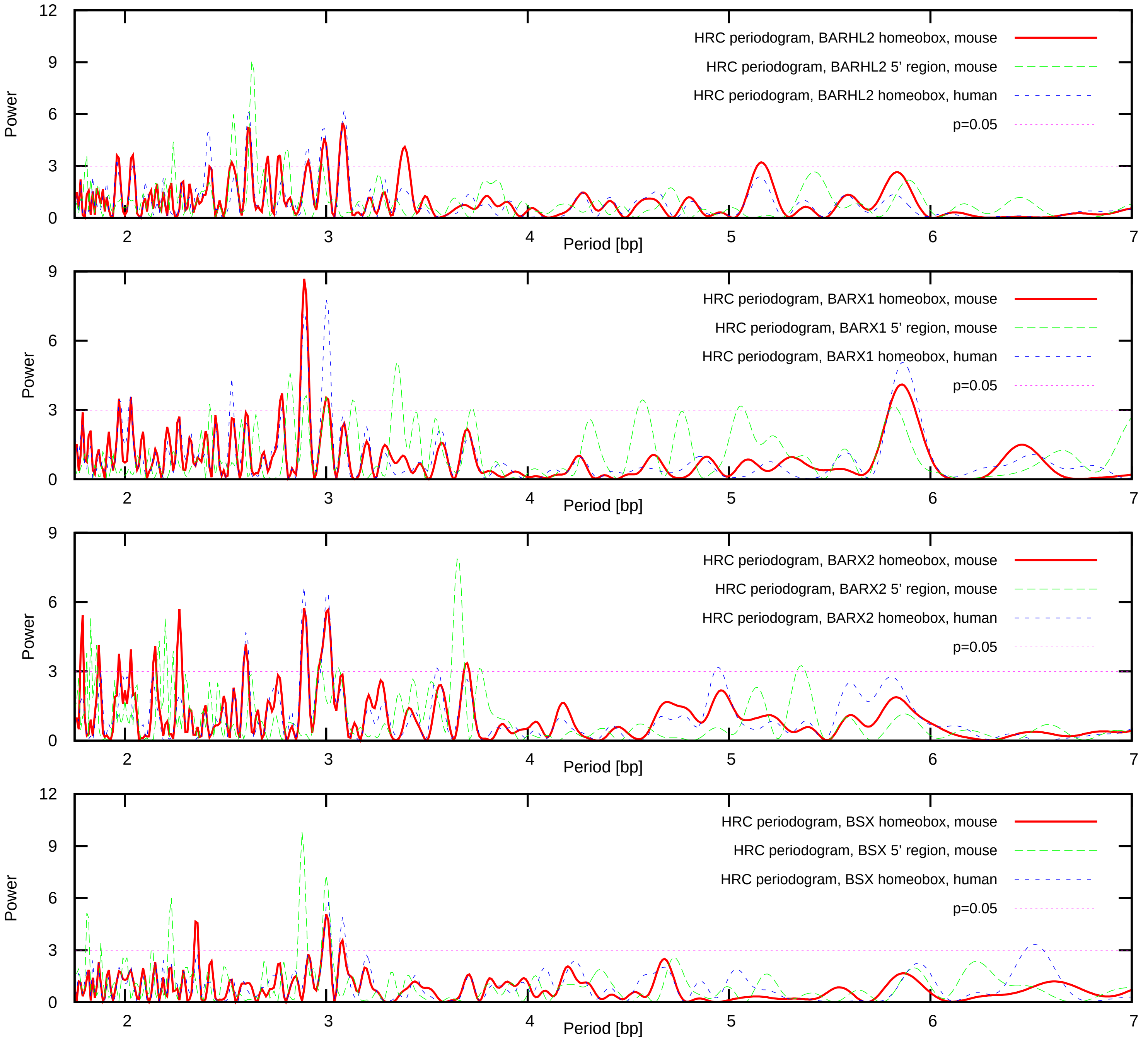

HRC periodogram, BARHL2 homeobox, mouse
HRC periodogram, BARHL2 5' region, mouse
HRC periodogram, BARHL2 homeobox, human
p=0.05
HRC periodogram, BARX1 homeobox, mouse
HRC periodogram, BARX1 5' region, mouse
HRC periodogram, BARX1 homeobox, human
p=0.05
HRC periodogram, BARX2 homeobox, mouse
HRC periodogram, BARX2 5' region, mouse
HRC periodogram, BARX2 homeobox, human
p=0.05
HRC periodogram, BSX homeobox, mouse
HRC periodogram, BSX 5' region, mouse
HRC periodogram, BSX homeobox, human
p=0.05
Power
Period [bp]
0
3
6
9
12
2
3
4
5
6
7

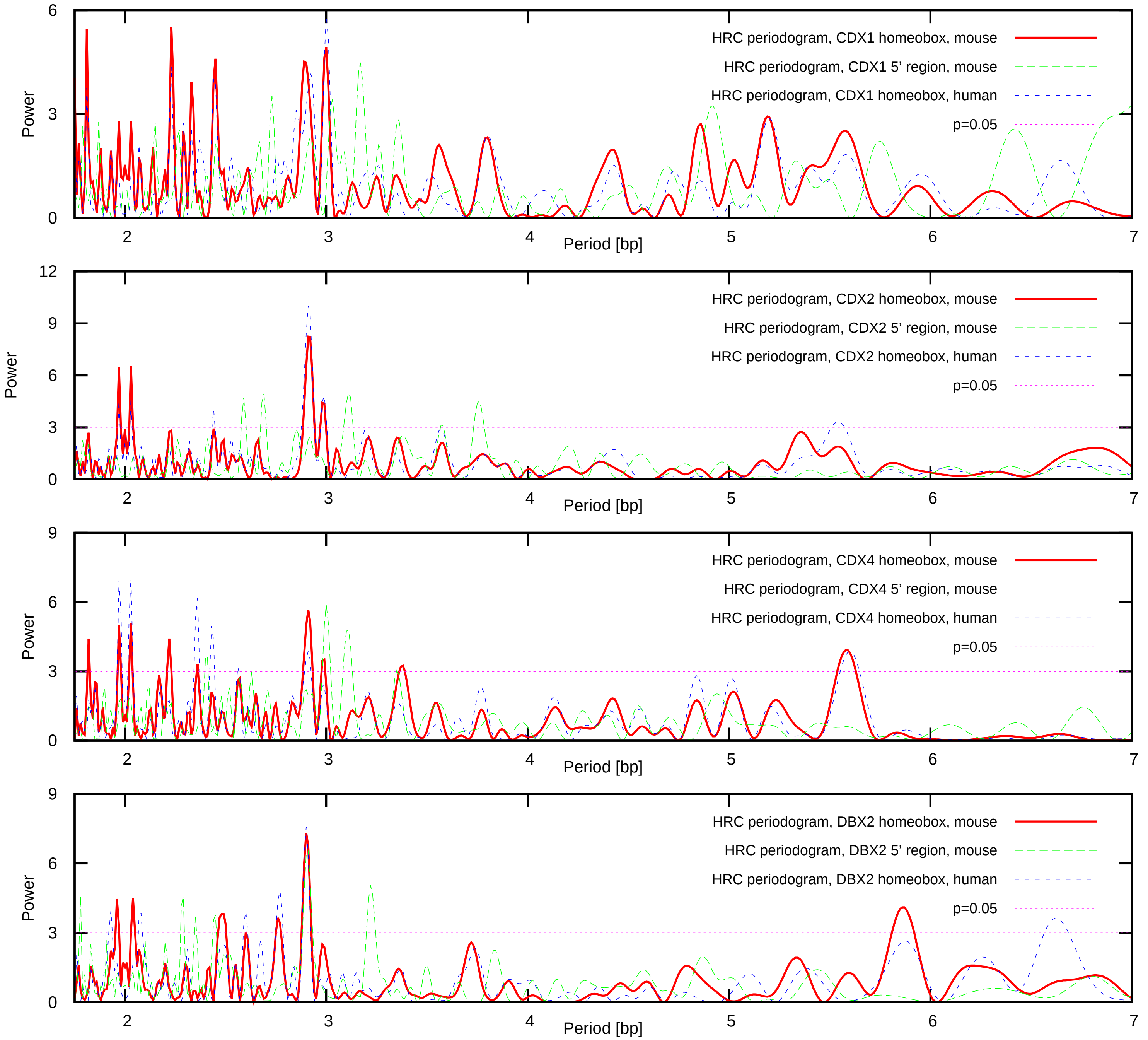

HRC periodogram, CDX1 homeobox, mouse
HRC periodogram, CDX1 5' region, mouse
HRC periodogram, CDX1 homeobox, human
p=0.05
Power
Period [bp]
HRC periodogram, CDX2 homeobox, mouse
HRC periodogram, CDX2 5' region, mouse
HRC periodogram, CDX2 homeobox, human
p=0.05
Power
Period [bp]
HRC periodogram, CDX4 homeobox, mouse
HRC periodogram, CDX4 5' region, mouse
HRC periodogram, CDX4 homeobox, human
p=0.05
Power
Period [bp]
HRC periodogram, DBX2 homeobox, mouse
HRC periodogram, DBX2 5' region, mouse
HRC periodogram, DBX2 homeobox, human
p=0.05
Power
Period [bp]

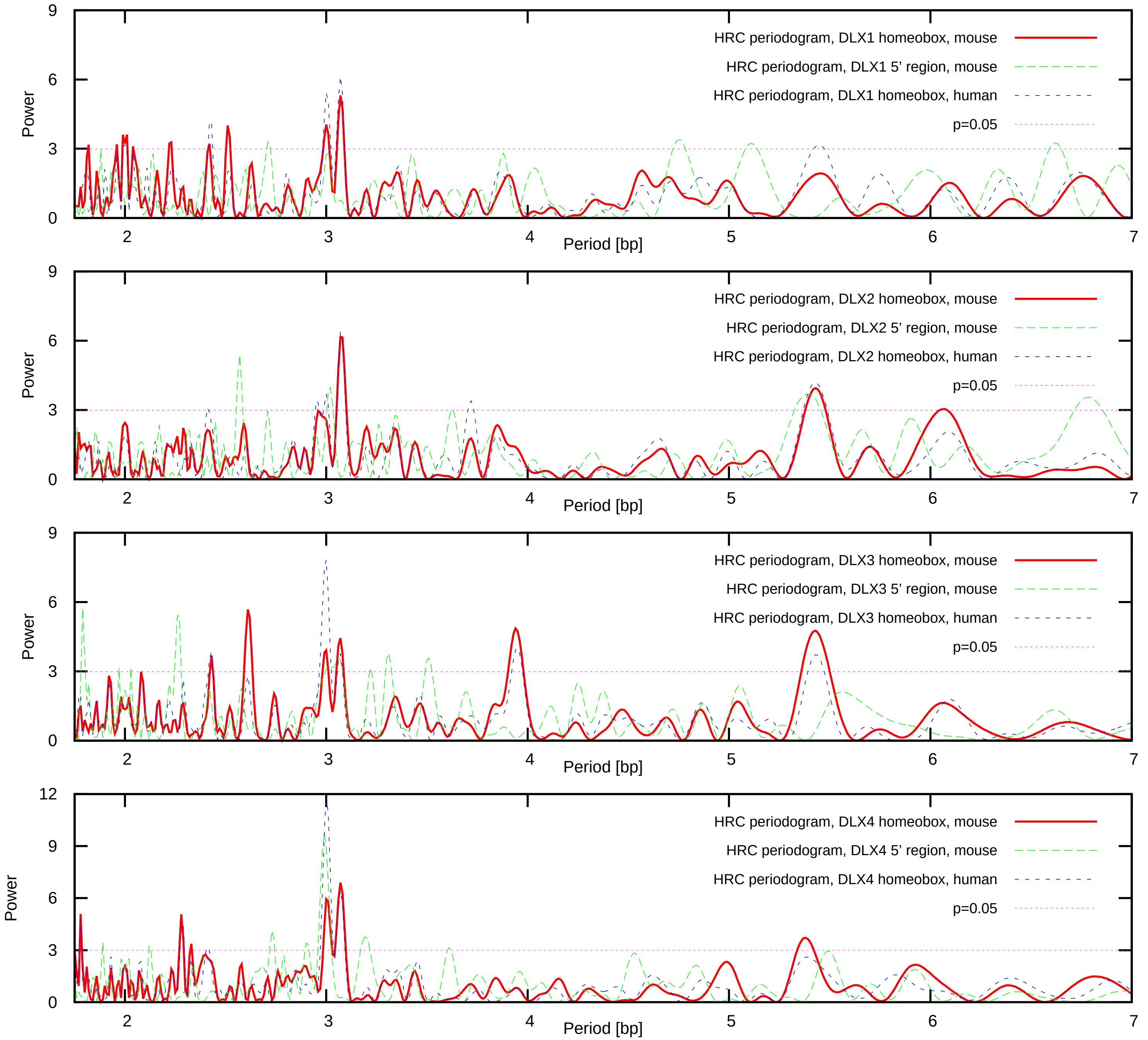

Power
Period [bp]
HRC periodogram, DLX1 homeobox, mouse
HRC periodogram, DLX1 5' region, mouse
HRC periodogram, DLX1 homeobox, human
p=0.05
HRC periodogram, DLX2 homeobox, mouse
HRC periodogram, DLX2 5' region, mouse
HRC periodogram, DLX2 homeobox, human
p=0.05
HRC periodogram, DLX3 homeobox, mouse
HRC periodogram, DLX3 5' region, mouse
HRC periodogram, DLX3 homeobox, human
p=0.05
HRC periodogram, DLX4 homeobox, mouse
HRC periodogram, DLX4 5' region, mouse
HRC periodogram, DLX4 homeobox, human
p=0.05

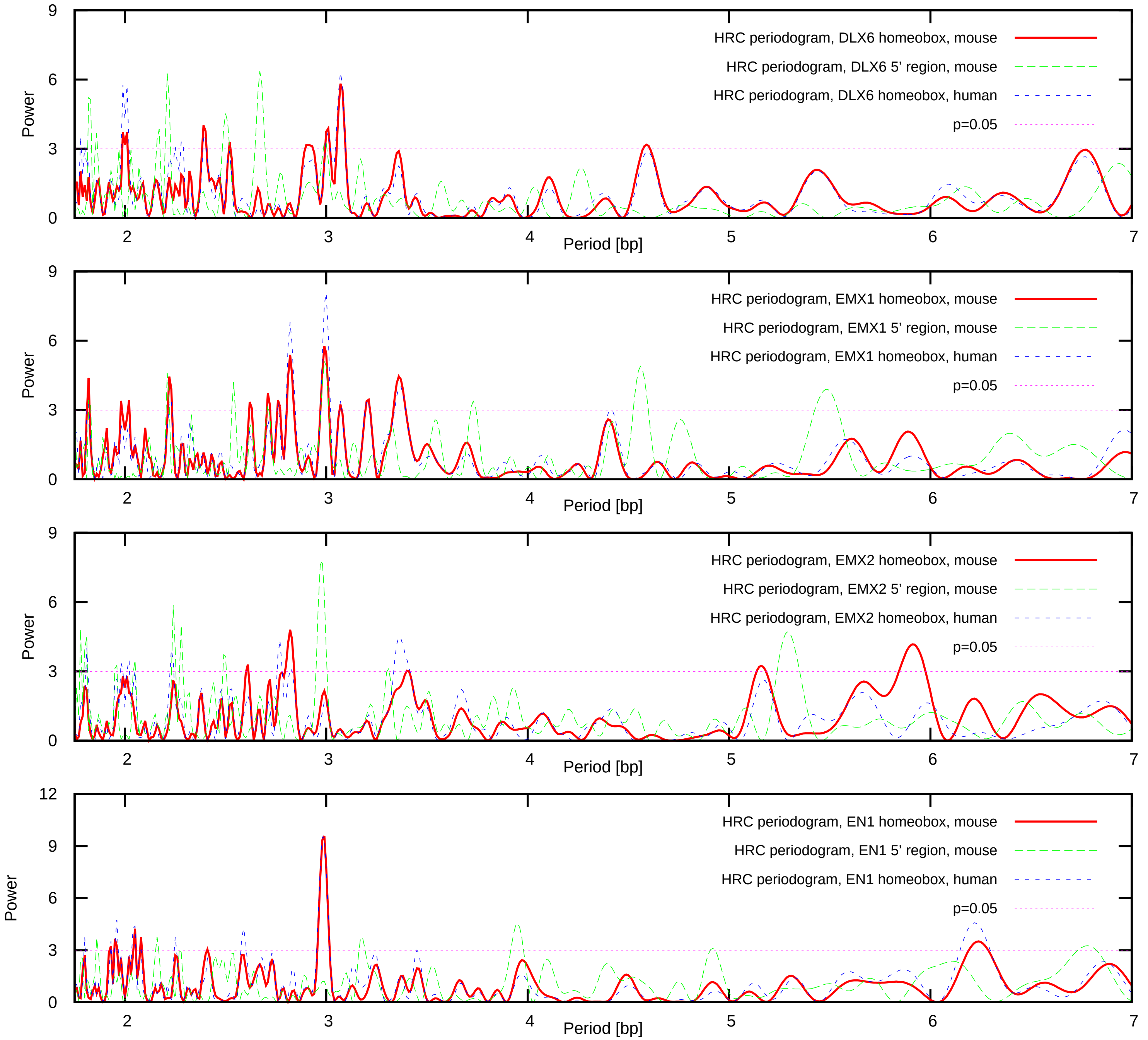
HRC periodogram, DLX6 homeobox, mouse
HRC periodogram, DLX6 5' region, mouse
HRC periodogram, DLX6 homeobox, human
p=0.05
HRC periodogram, EMX1 homeobox, mouse
HRC periodogram, EMX1 5' region, mouse
HRC periodogram, EMX1 homeobox, human
p=0.05
HRC periodogram, EMX2 homeobox, mouse
HRC periodogram, EMX2 5' region, mouse
HRC periodogram, EMX2 homeobox, human
p=0.05
HRC periodogram, EN1 homeobox, mouse
HRC periodogram, EN1 5' region, mouse
HRC periodogram, EN1 homeobox, human
p=0.05
Power
Period [bp]

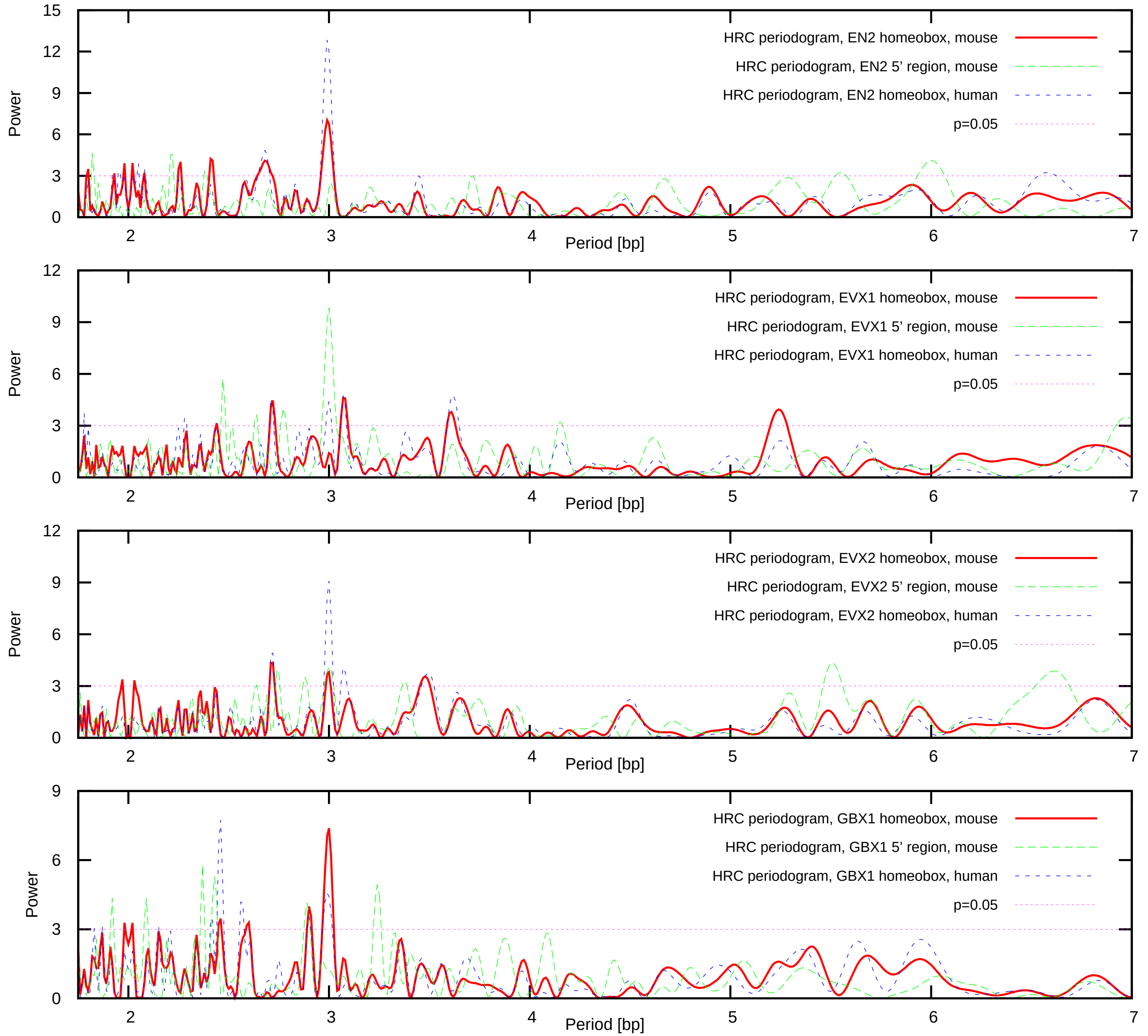

HRC periodogram, EN2 homeobox, mouse
HRC periodogram, EN2 5' region, mouse
HRC periodogram, EN2 homeobox, human
p=0.05
HRC periodogram, EVX1 homeobox, mouse
HRC periodogram, EVX1 5' region, mouse
HRC periodogram, EVX1 homeobox, human
p=0.05
HRC periodogram, EVX2 homeobox, mouse
HRC periodogram, EVX2 5' region, mouse
HRC periodogram, EVX2 homeobox, human
p=0.05
HRC periodogram, GBX1 homeobox, mouse
HRC periodogram, GBX1 5' region, mouse
HRC periodogram, GBX1 homeobox, human
p=0.05
Power
Period [bp]

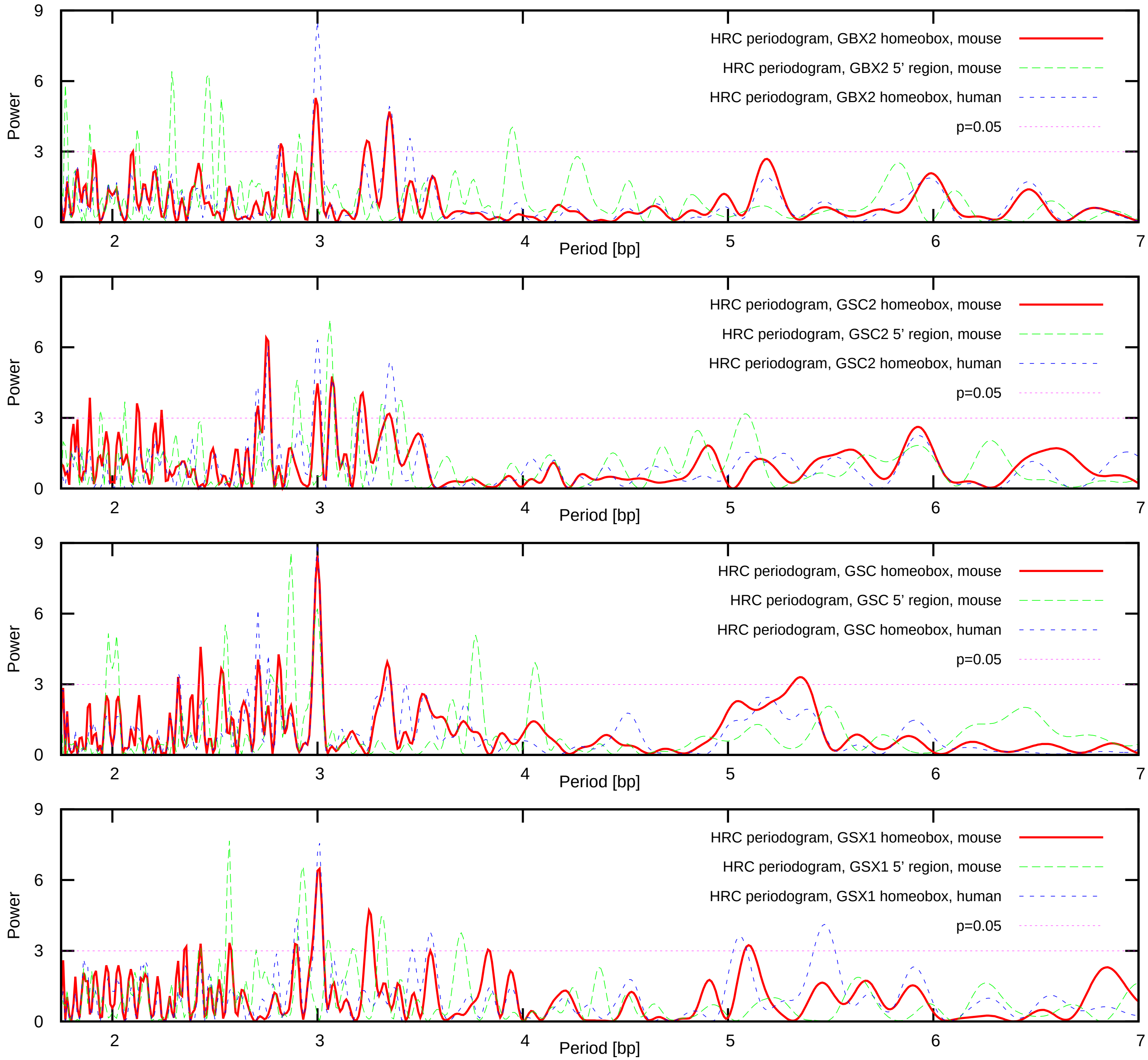

HRC periodogram, GBX2 homeobox, mouse
HRC periodogram, GBX2 5' region, mouse
HRC periodogram, GBX2 homeobox, human
p=0.05
HRC periodogram, GSC2 homeobox, mouse
HRC periodogram, GSC2 5' region, mouse
HRC periodogram, GSC2 homeobox, human
p=0.05
HRC periodogram, GSC homeobox, mouse
HRC periodogram, GSC 5' region, mouse
HRC periodogram, GSC homeobox, human
p=0.05
HRC periodogram, GSX1 homeobox, mouse
HRC periodogram, GSX1 5' region, mouse
HRC periodogram, GSX1 homeobox, human
p=0.05
Power
Period [bp]
0
3
6
9
2
3
4
5
6
7

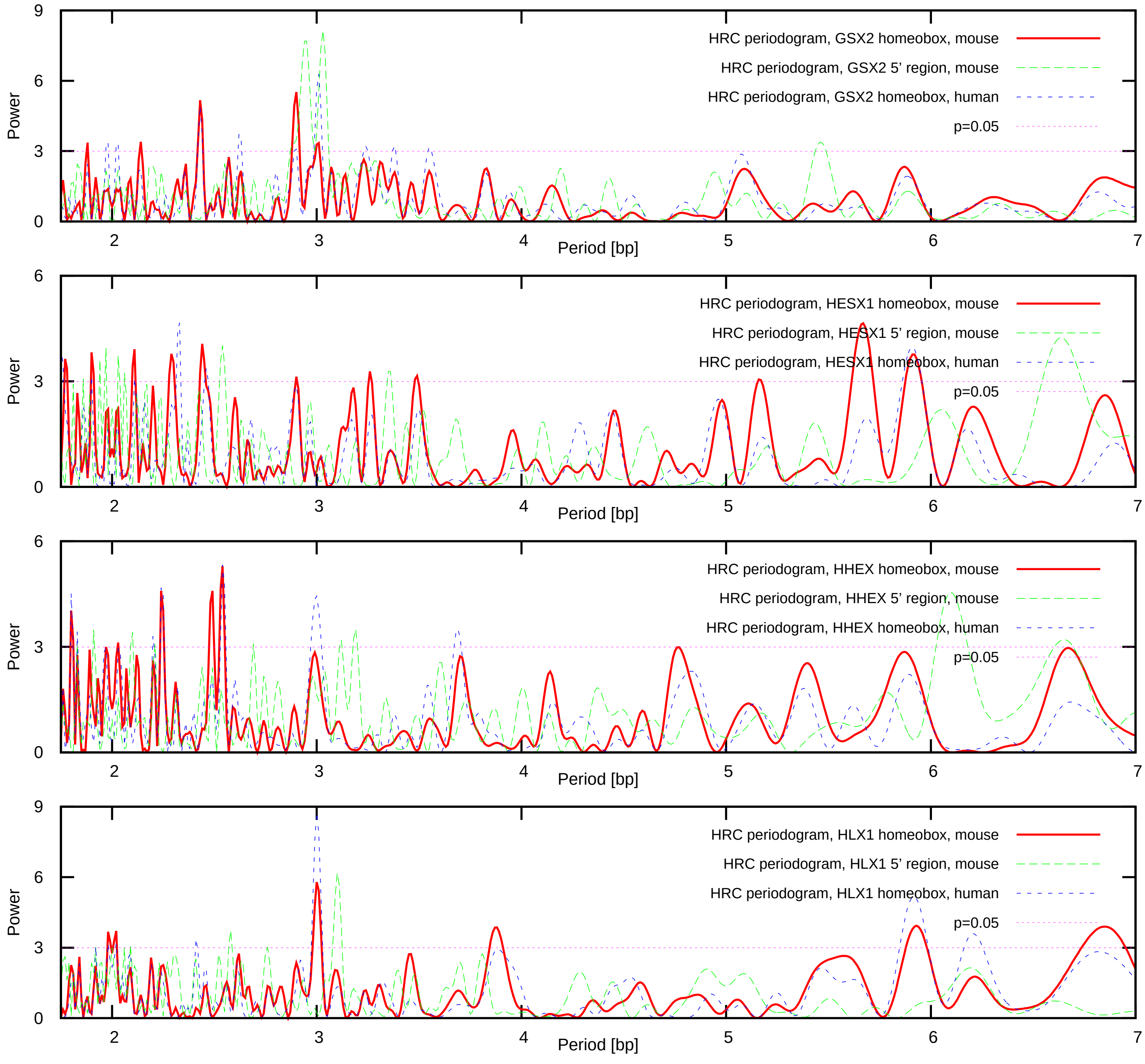

HRC periodogram, GSX2 homeobox, mouse
HRC periodogram, GSX2 5' region, mouse
HRC periodogram, GSX2 homeobox, human
p=0.05
Power
Period [bp]
HRC periodogram, HESX1 homeobox, mouse
HRC periodogram, HESX1 5' region, mouse
HRC periodogram, HESX1 homeobox, human
p=0.05
Power
Period [bp]
HRC periodogram, HHEX homeobox, mouse
HRC periodogram, HHEX 5' region, mouse
HRC periodogram, HHEX homeobox, human
p=0.05
Power
Period [bp]
HRC periodogram, HLX1 homeobox, mouse
HRC periodogram, HLX1 5' region, mouse
HRC periodogram, HLX1 homeobox, human
p=0.05
Power
Period [bp]

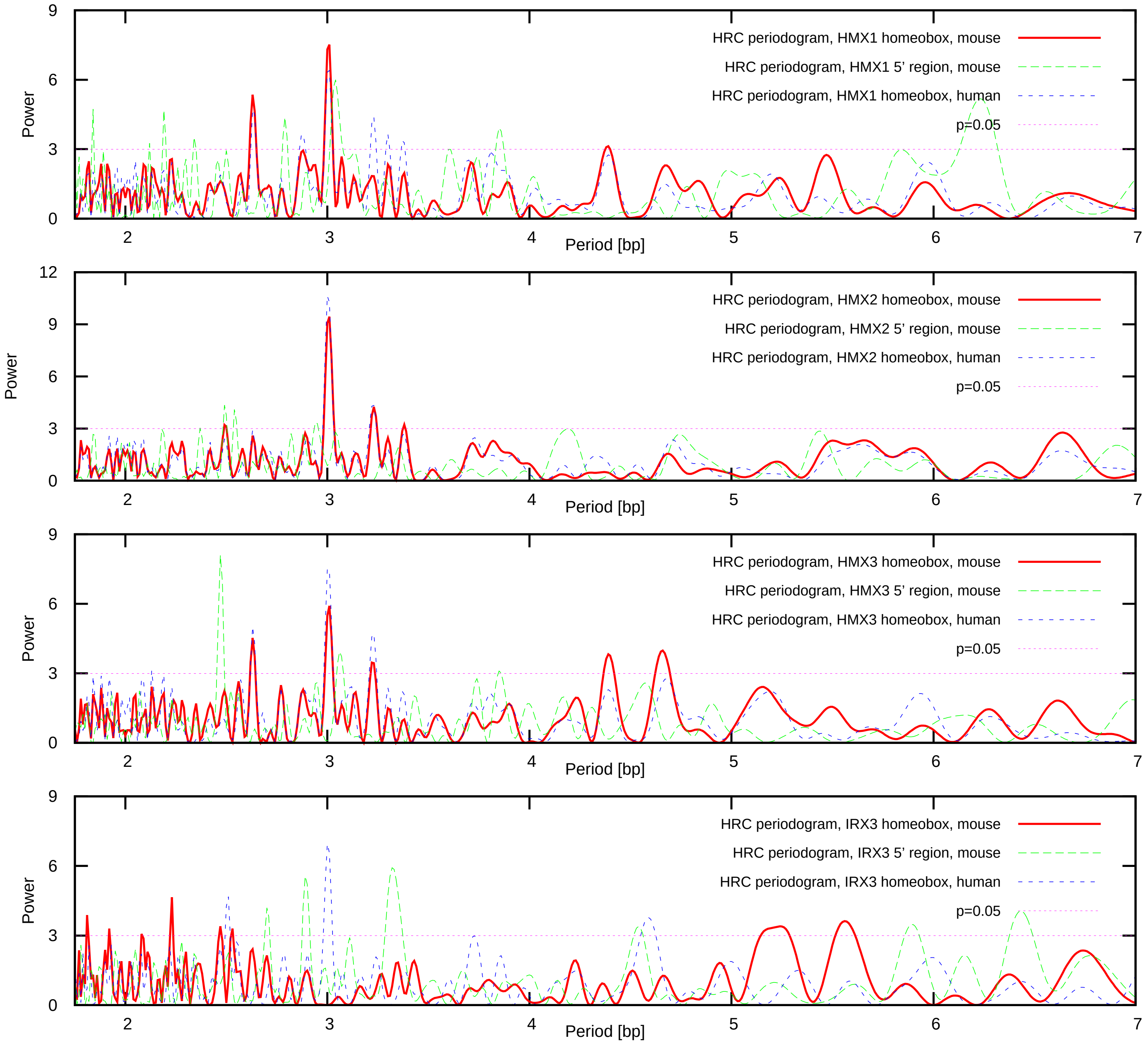

Power
Period [bp]
HRC periodogram, HMX1 homeobox, mouse
HRC periodogram, HMX1 5' region, mouse
HRC periodogram, HMX1 homeobox, human
p=0.05
HRC periodogram, HMX2 homeobox, mouse
HRC periodogram, HMX2 5' region, mouse
HRC periodogram, HMX2 homeobox, human
p=0.05
HRC periodogram, HMX3 homeobox, mouse
HRC periodogram, HMX3 5' region, mouse
HRC periodogram, HMX3 homeobox, human
p=0.05
HRC periodogram, IRX3 homeobox, mouse
HRC periodogram, IRX3 5' region, mouse
HRC periodogram, IRX3 homeobox, human
p=0.05

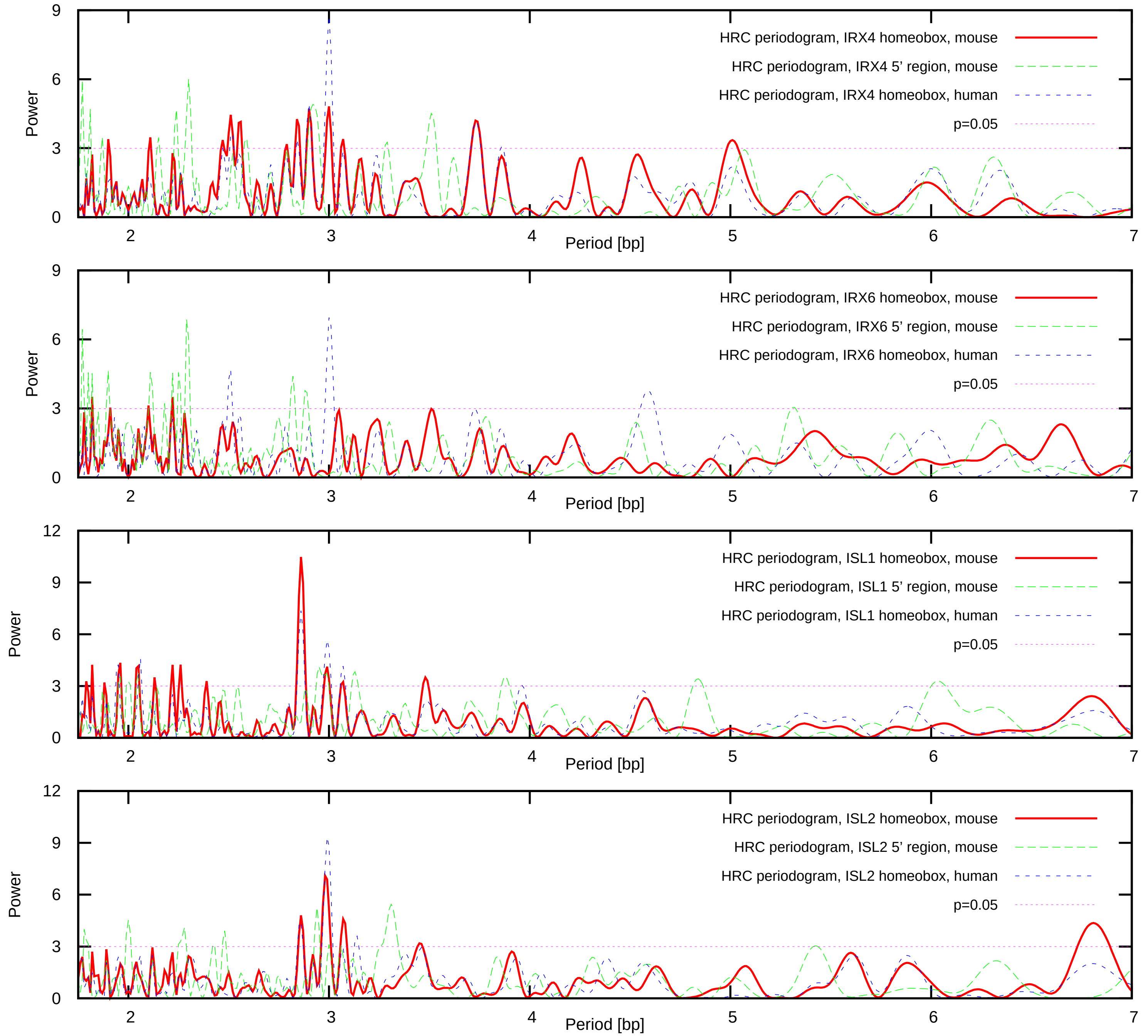
HRC periodogram, IRX4 homeobox, mouse
HRC periodogram, IRX4 5' region, mouse
HRC periodogram, IRX4 homeobox, human
p=0.05
Power
Period [bp]
HRC periodogram, IRX6 homeobox, mouse
HRC periodogram, IRX6 5' region, mouse
HRC periodogram, IRX6 homeobox, human
p=0.05
Power
Period [bp]
HRC periodogram, ISL1 homeobox, mouse
HRC periodogram, ISL1 5' region, mouse
HRC periodogram, ISL1 homeobox, human
p=0.05
Power
Period [bp]
HRC periodogram, ISL2 homeobox, mouse
HRC periodogram, ISL2 5' region, mouse
HRC periodogram, ISL2 homeobox, human
p=0.05
Power
Period [bp]

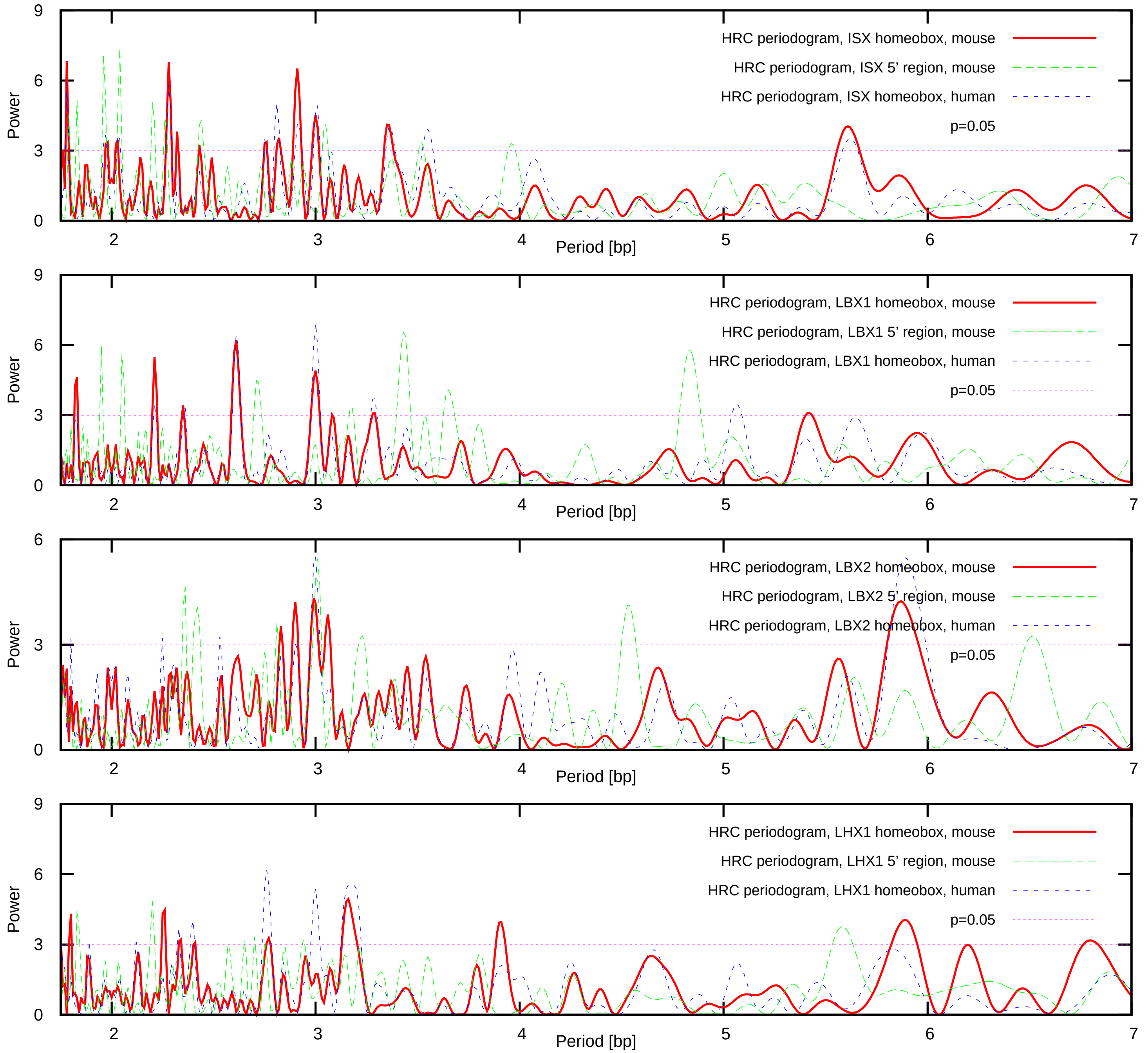

Power
Period [bp]
HRC periodogram, ISX homeobox, mouse
HRC periodogram, ISX 5' region, mouse
HRC periodogram, ISX homeobox, human
p=0.05
HRC periodogram, LBX1 homeobox, mouse
HRC periodogram, LBX1 5' region, mouse
HRC periodogram, LBX1 homeobox, human
HRC periodogram, LBX2 homeobox, mouse
HRC periodogram, LBX2 5' region, mouse
HRC periodogram, LBX2 homeobox, human
HRC periodogram, LHX1 homeobox, mouse
HRC periodogram, LHX1 5' region, mouse
HRC periodogram, LHX1 homeobox, human

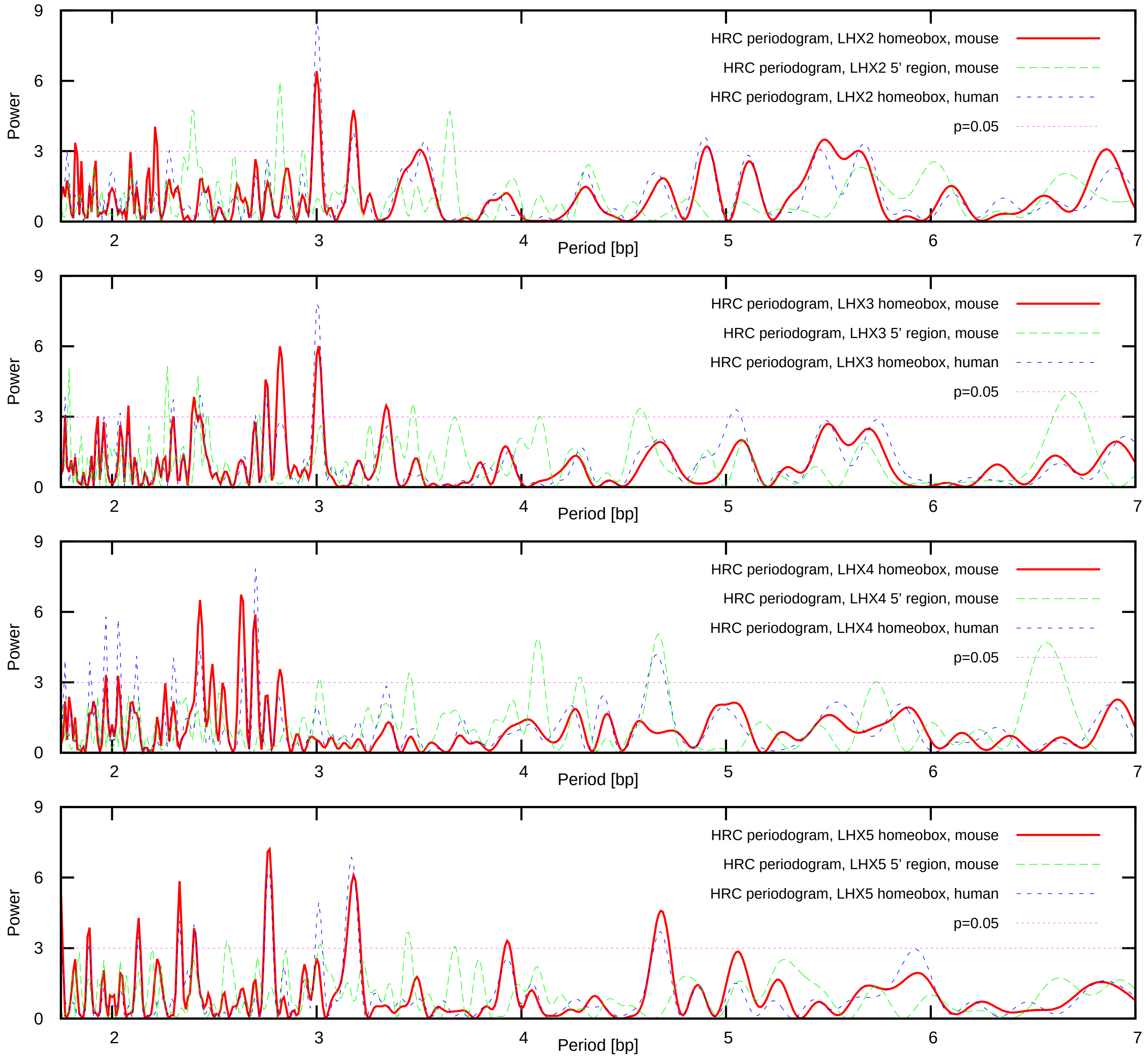

HRC periodogram, LHX2 homeobox, mouse
HRC periodogram, LHX2 5' region, mouse
HRC periodogram, LHX2 homeobox, human
p=0.05
HRC periodogram, LHX3 homeobox, mouse
HRC periodogram, LHX3 5' region, mouse
HRC periodogram, LHX3 homeobox, human
p=0.05
HRC periodogram, LHX4 homeobox, mouse
HRC periodogram, LHX4 5' region, mouse
HRC periodogram, LHX4 homeobox, human
p=0.05
HRC periodogram, LHX5 homeobox, mouse
HRC periodogram, LHX5 5' region, mouse
HRC periodogram, LHX5 homeobox, human
p=0.05
Power
Period [bp]
0
3
6
9
2
3
4
5
6
7

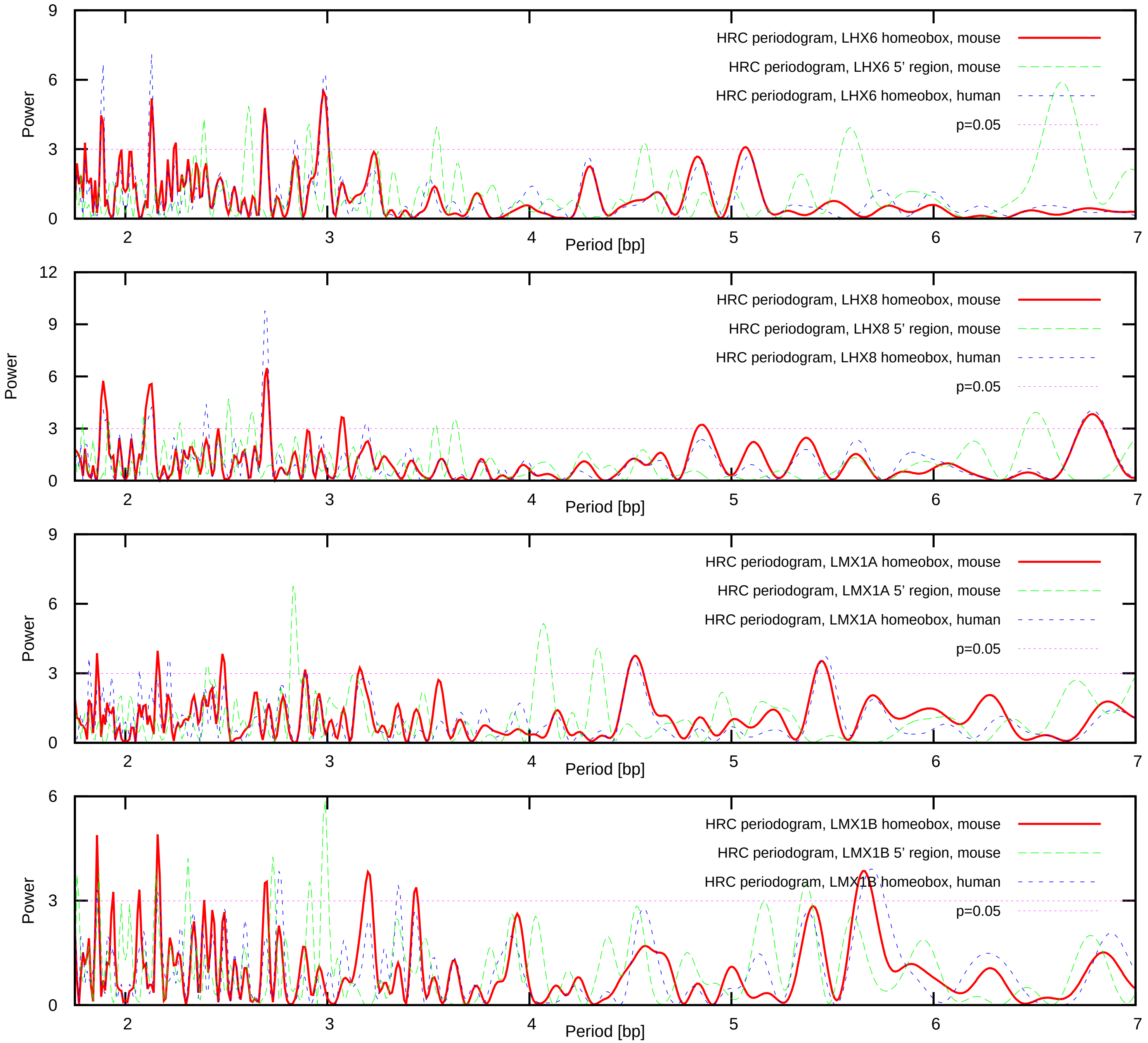

HRC periodogram, LHX6 homeobox, mouse
HRC periodogram, LHX6 5' region, mouse
HRC periodogram, LHX6 homeobox, human
p=0.05
Power
Period [bp]
HRC periodogram, LHX8 homeobox, mouse
HRC periodogram, LHX8 5' region, mouse
HRC periodogram, LHX8 homeobox, human
p=0.05
Power
Period [bp]
HRC periodogram, LMX1A homeobox, mouse
HRC periodogram, LMX1A 5' region, mouse
HRC periodogram, LMX1A homeobox, human
p=0.05
Power
Period [bp]
HRC periodogram, LMX1B homeobox, mouse
HRC periodogram, LMX1B 5' region, mouse
HRC periodogram, LMX1B homeobox, human
p=0.05
Power
Period [bp]

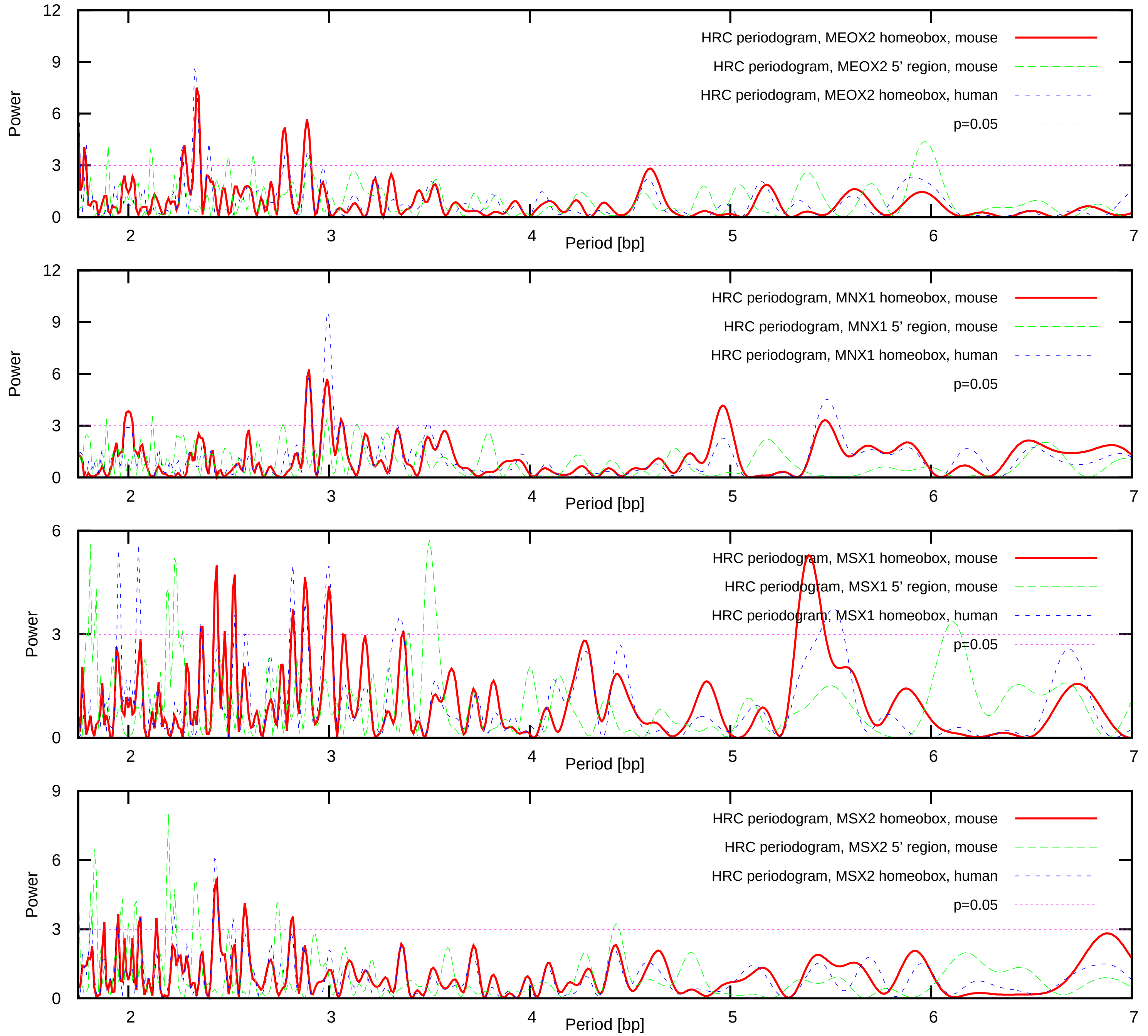

HRC periodogram, MEOX2 homeobox, mouse
HRC periodogram, MEOX2 5' region, mouse
HRC periodogram, MEOX2 homeobox, human
p=0.05
Power
Period [bp]
HRC periodogram, MNX1 homeobox, mouse
HRC periodogram, MNX1 5' region, mouse
HRC periodogram, MNX1 homeobox, human
p=0.05
Power
Period [bp]
HRC periodogram, MSX1 homeobox, mouse
HRC periodogram, MSX1 5' region, mouse
HRC periodogram, MSX1 homeobox, human
p=0.05
Power
Period [bp]
HRC periodogram, MSX2 homeobox, mouse
HRC periodogram, MSX2 5' region, mouse
HRC periodogram, MSX2 homeobox, human
p=0.05
Power
Period [bp]

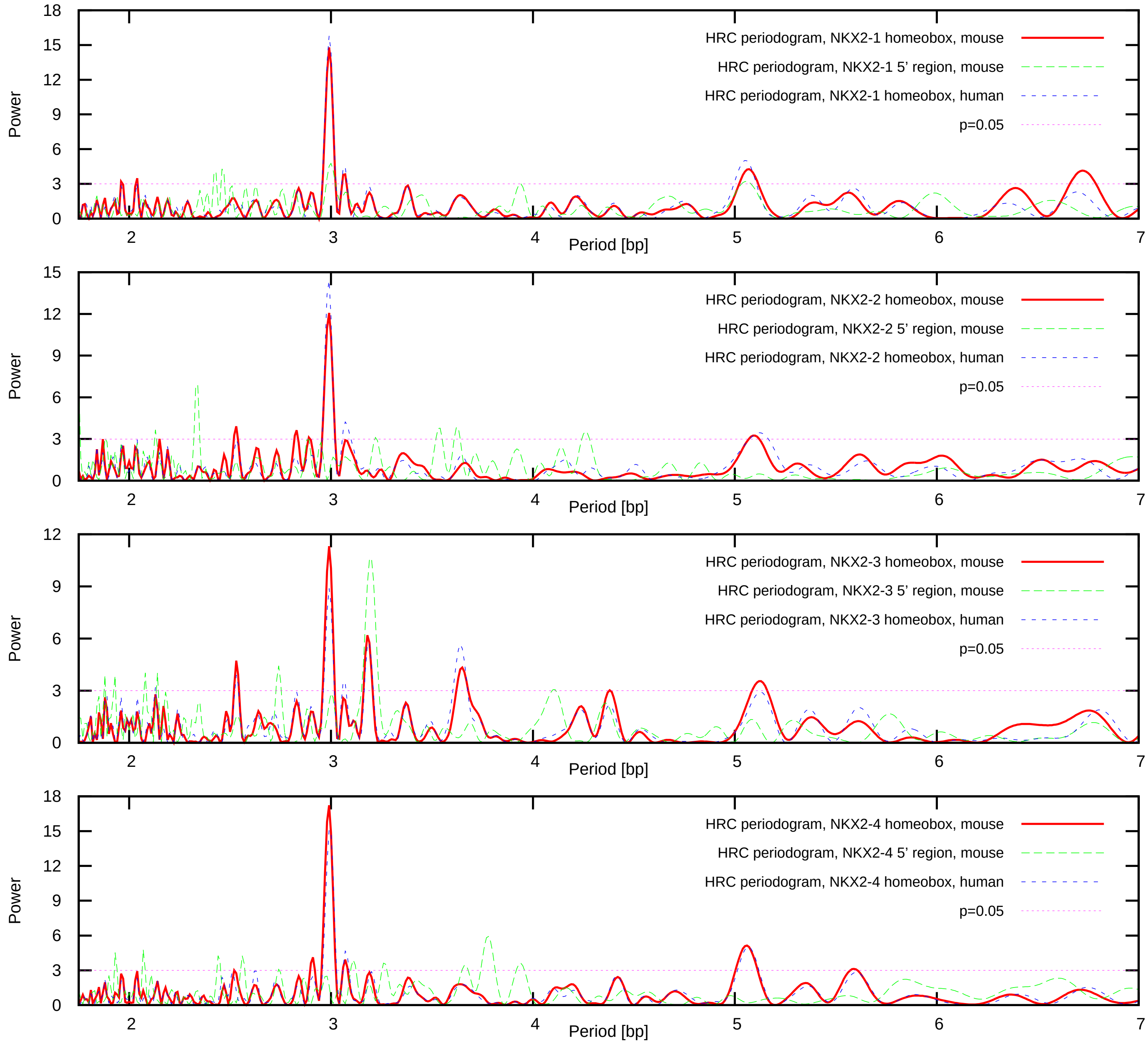

Power
Period [bp]
HRC periodogram, NKX2-1 homeobox, mouse
HRC periodogram, NKX2-1 5' region, mouse
HRC periodogram, NKX2-1 homeobox, human
p=0.05
HRC periodogram, NKX2-2 homeobox, mouse
HRC periodogram, NKX2-2 5' region, mouse
HRC periodogram, NKX2-2 homeobox, human
p=0.05
HRC periodogram, NKX2-3 homeobox, mouse
HRC periodogram, NKX2-3 5' region, mouse
HRC periodogram, NKX2-3 homeobox, human
p=0.05
HRC periodogram, NKX2-4 homeobox, mouse
HRC periodogram, NKX2-4 5' region, mouse
HRC periodogram, NKX2-4 homeobox, human
p=0.05

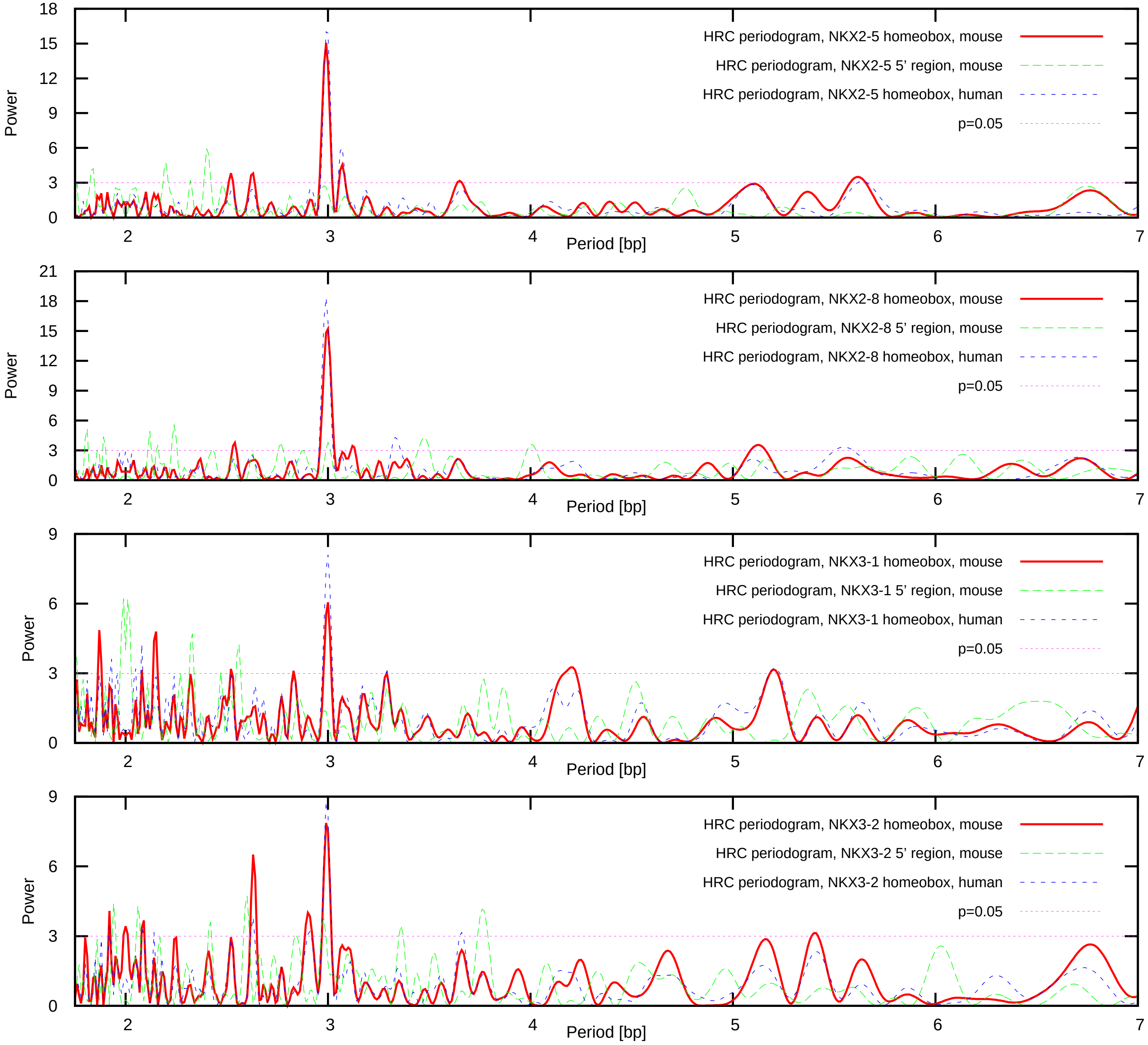

Power
Period [bp]
HRC periodogram, NKX2-5 homeobox, mouse
HRC periodogram, NKX2-5 5' region, mouse
HRC periodogram, NKX2-5 homeobox, human
p=0.05
HRC periodogram, NKX2-8 homeobox, mouse
HRC periodogram, NKX2-8 5' region, mouse
HRC periodogram, NKX2-8 homeobox, human
p=0.05
HRC periodogram, NKX3-1 homeobox, mouse
HRC periodogram, NKX3-1 5' region, mouse
HRC periodogram, NKX3-1 homeobox, human
p=0.05
HRC periodogram, NKX3-2 homeobox, mouse
HRC periodogram, NKX3-2 5' region, mouse
HRC periodogram, NKX3-2 homeobox, human
p=0.05

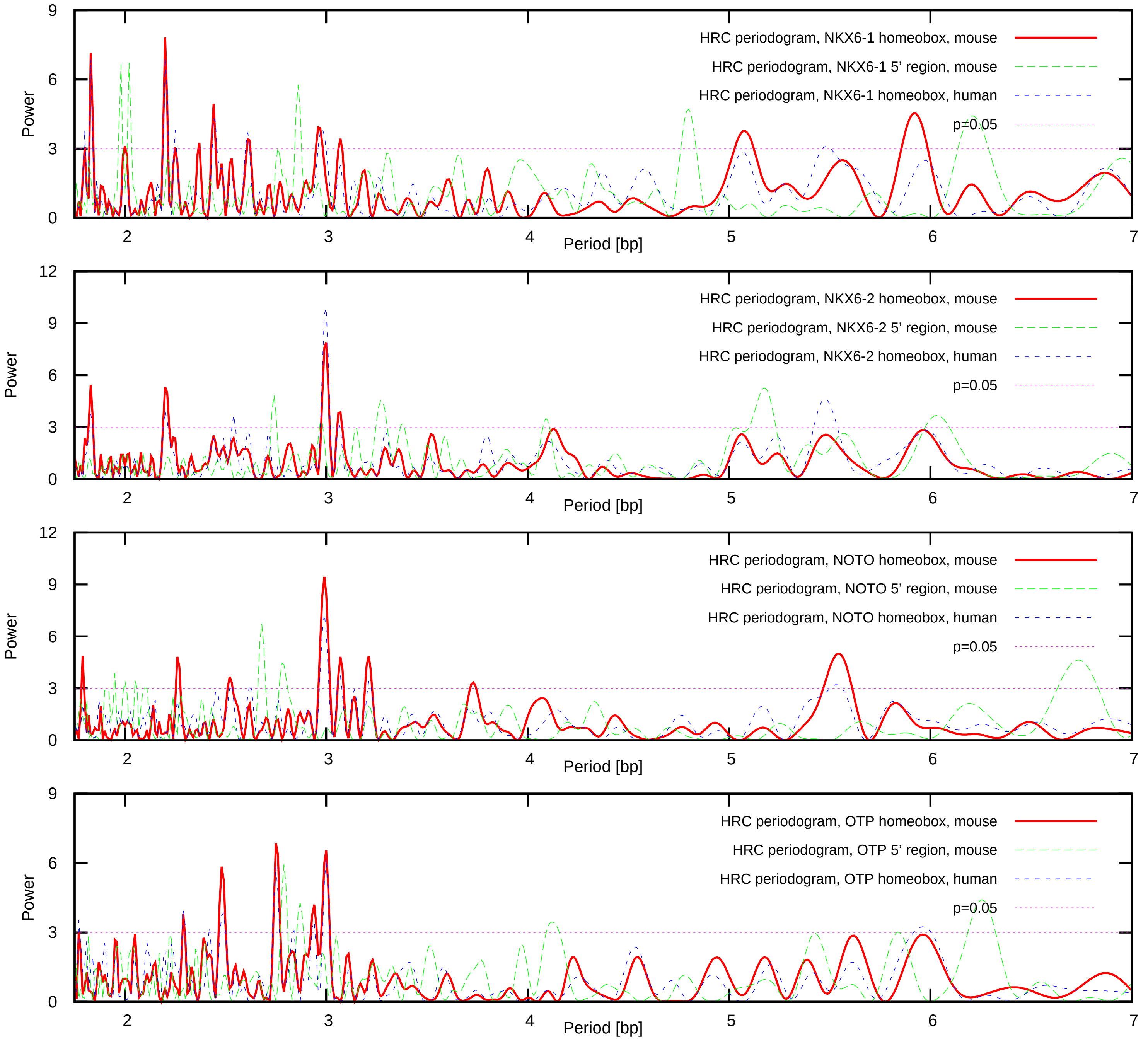
HRC periodogram, NKX6-1 homeobox, mouse
HRC periodogram, NKX6-1 5' region, mouse
HRC periodogram, NKX6-1 homeobox, human
p=0.05
Power
Period [bp]
HRC periodogram, NKX6-2 homeobox, mouse
HRC periodogram, NKX6-2 5' region, mouse
HRC periodogram, NKX6-2 homeobox, human
p=0.05
Power
Period [bp]
HRC periodogram, NOTO homeobox, mouse
HRC periodogram, NOTO 5' region, mouse
HRC periodogram, NOTO homeobox, human
p=0.05
Power
Period [bp]
HRC periodogram, OTP homeobox, mouse
HRC periodogram, OTP 5' region, mouse
HRC periodogram, OTP homeobox, human
p=0.05
Power
Period [bp]

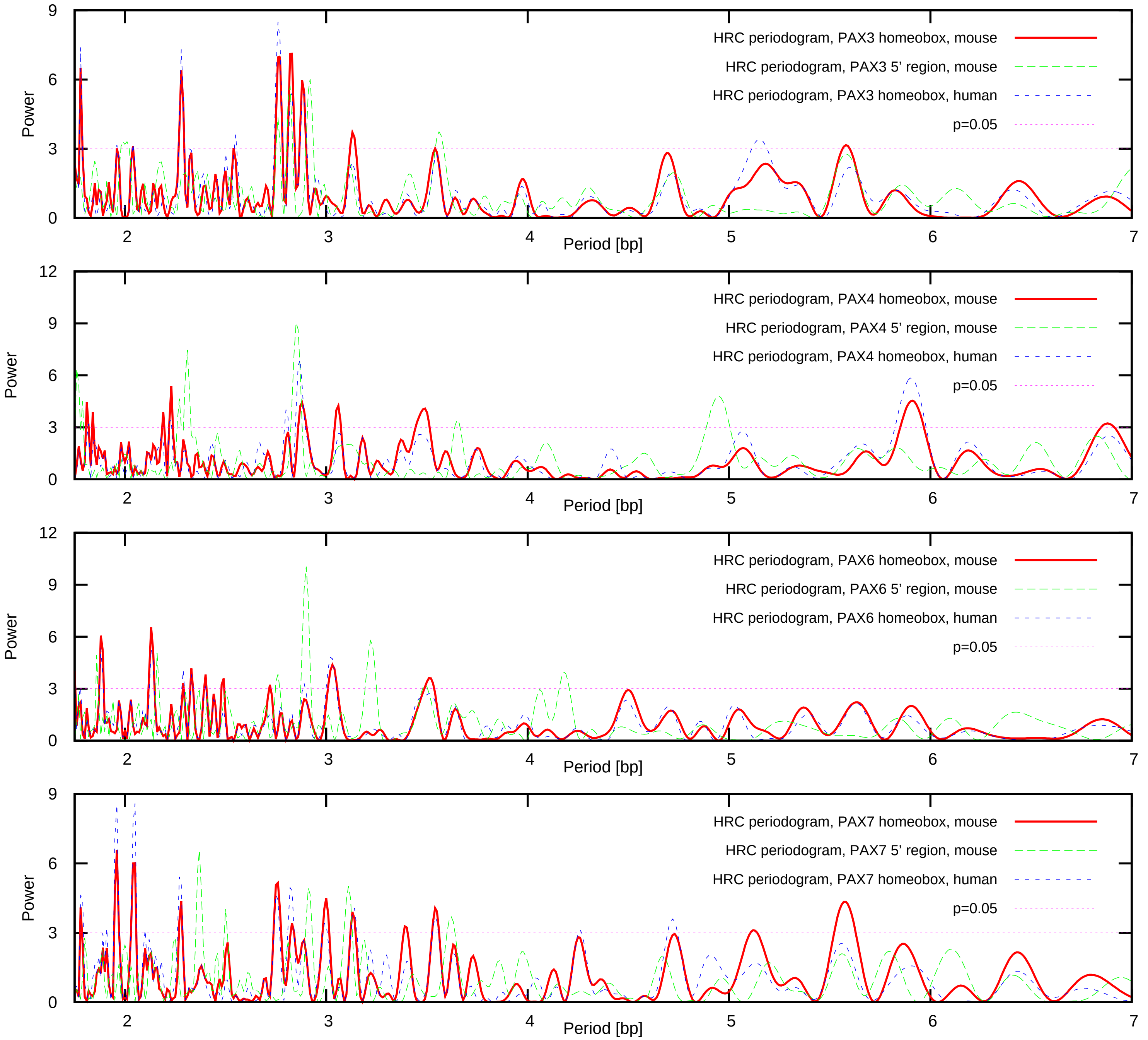
HRC periodogram, PAX3 homeobox, mouse
HRC periodogram, PAX3 5' region, mouse
HRC periodogram, PAX3 homeobox, human
p=0.05
HRC periodogram, PAX4 homeobox, mouse
HRC periodogram, PAX4 5' region, mouse
HRC periodogram, PAX4 homeobox, human
p=0.05
HRC periodogram, PAX6 homeobox, mouse
HRC periodogram, PAX6 5' region, mouse
HRC periodogram, PAX6 homeobox, human
p=0.05
HRC periodogram, PAX7 homeobox, mouse
HRC periodogram, PAX7 5' region, mouse
HRC periodogram, PAX7 homeobox, human
p=0.05
Power
Period [bp]
0
3
6
9
12
2
3
4
5
6
7

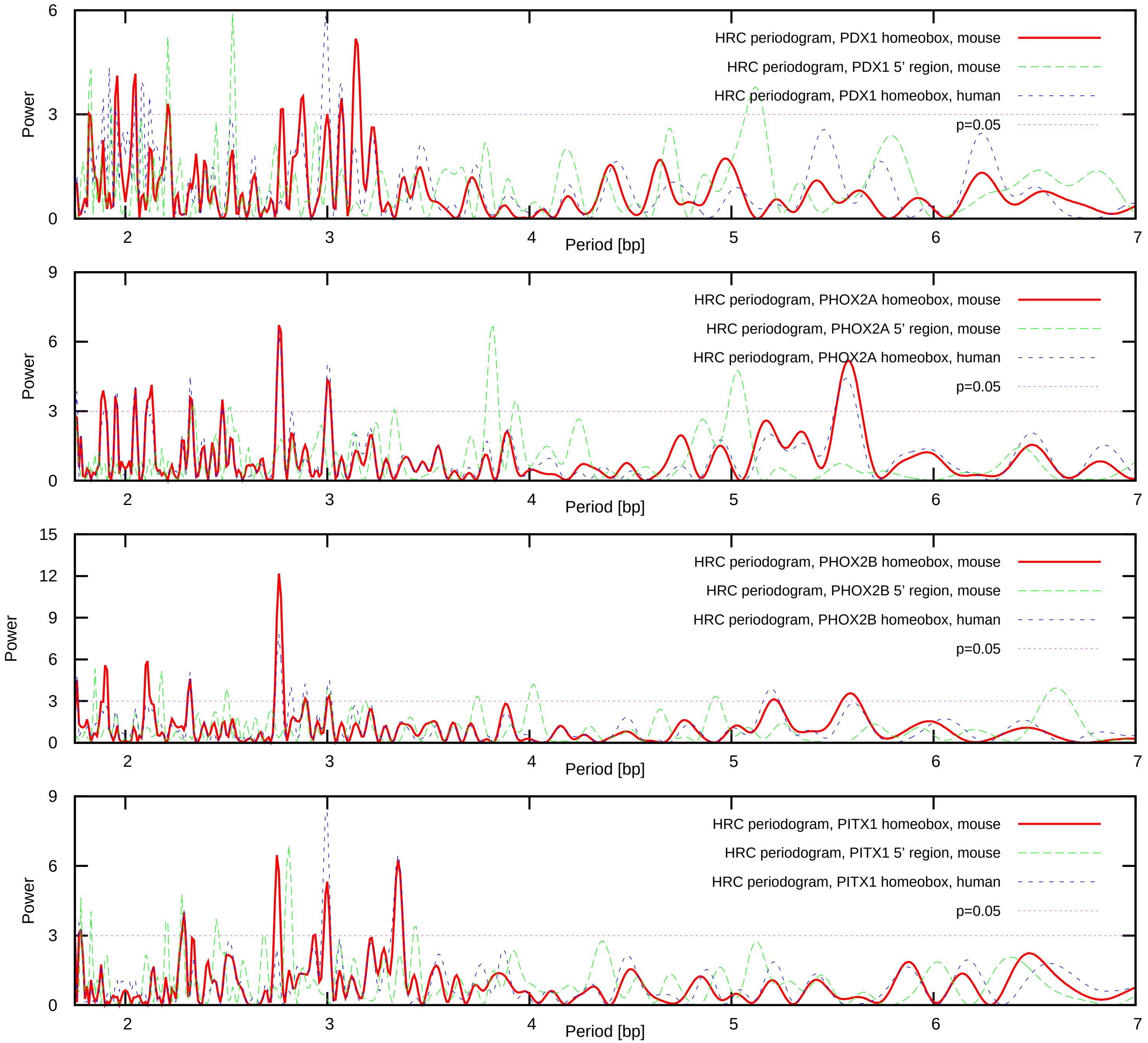

HRC periodogram, PDX1 homeobox, mouse
HRC periodogram, PDX1 5' region, mouse
HRC periodogram, PDX1 homeobox, human
p=0.05
Power
Period [bp]
HRC periodogram, PHOX2A homeobox, mouse
HRC periodogram, PHOX2A 5' region, mouse
HRC periodogram, PHOX2A homeobox, human
p=0.05
Power
Period [bp]
HRC periodogram, PHOX2B homeobox, mouse
HRC periodogram, PHOX2B 5' region, mouse
HRC periodogram, PHOX2B homeobox, human
p=0.05
Power
Period [bp]
HRC periodogram, PITX1 homeobox, mouse
HRC periodogram, PITX1 5' region, mouse
HRC periodogram, PITX1 homeobox, human
p=0.05
Power
Period [bp]

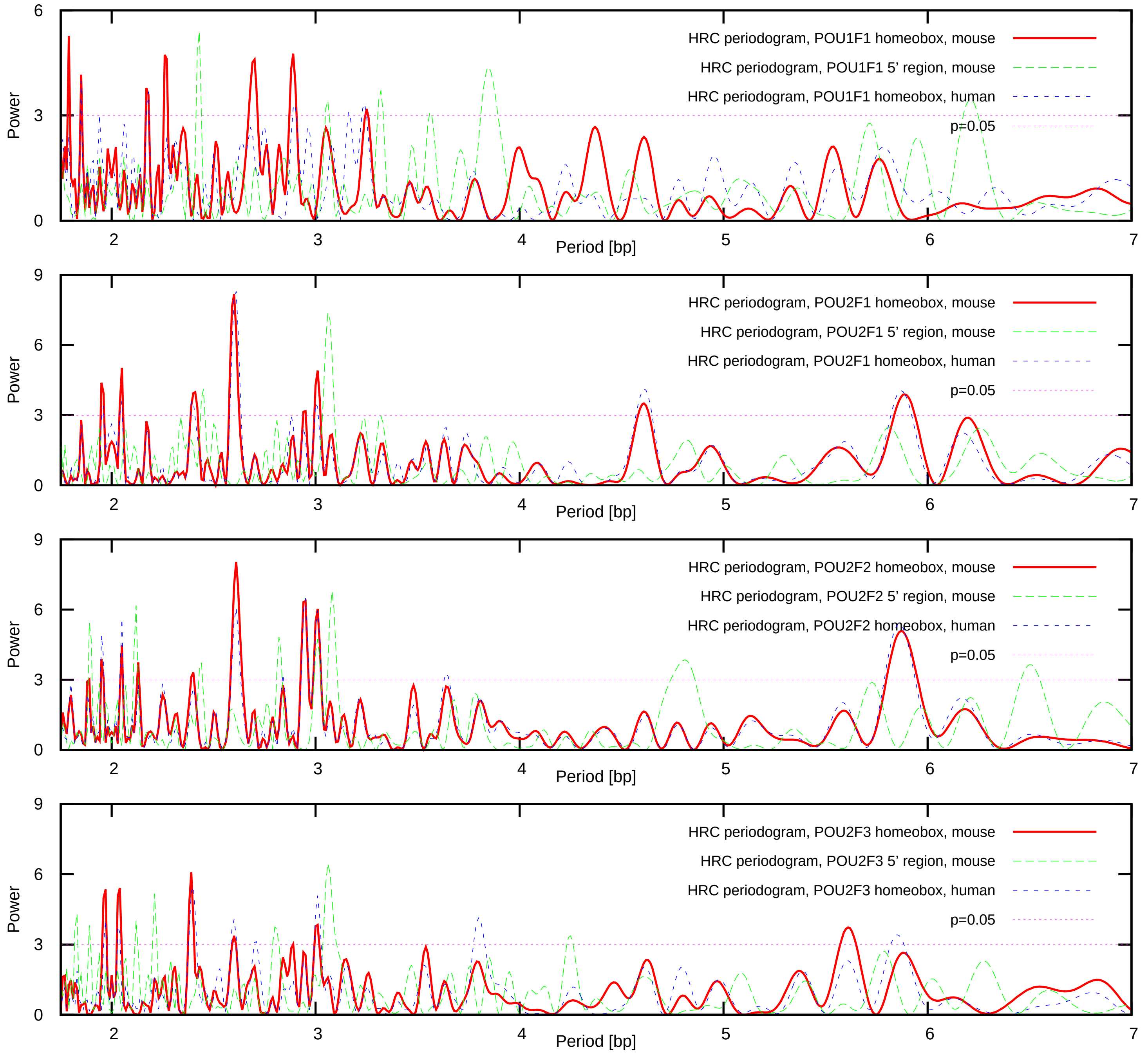
HRC periodogram, POU1F1 homeobox, mouse
HRC periodogram, POU1F1 5' region, mouse
HRC periodogram, POU1F1 homeobox, human
p=0.05
Power
Period [bp]
HRC periodogram, POU2F1 homeobox, mouse
HRC periodogram, POU2F1 5' region, mouse
HRC periodogram, POU2F1 homeobox, human
p=0.05
Power
Period [bp]
HRC periodogram, POU2F2 homeobox, mouse
HRC periodogram, POU2F2 5' region, mouse
HRC periodogram, POU2F2 homeobox, human
p=0.05
Power
Period [bp]
HRC periodogram, POU2F3 homeobox, mouse
HRC periodogram, POU2F3 5' region, mouse
HRC periodogram, POU2F3 homeobox, human
p=0.05
Power
Period [bp]

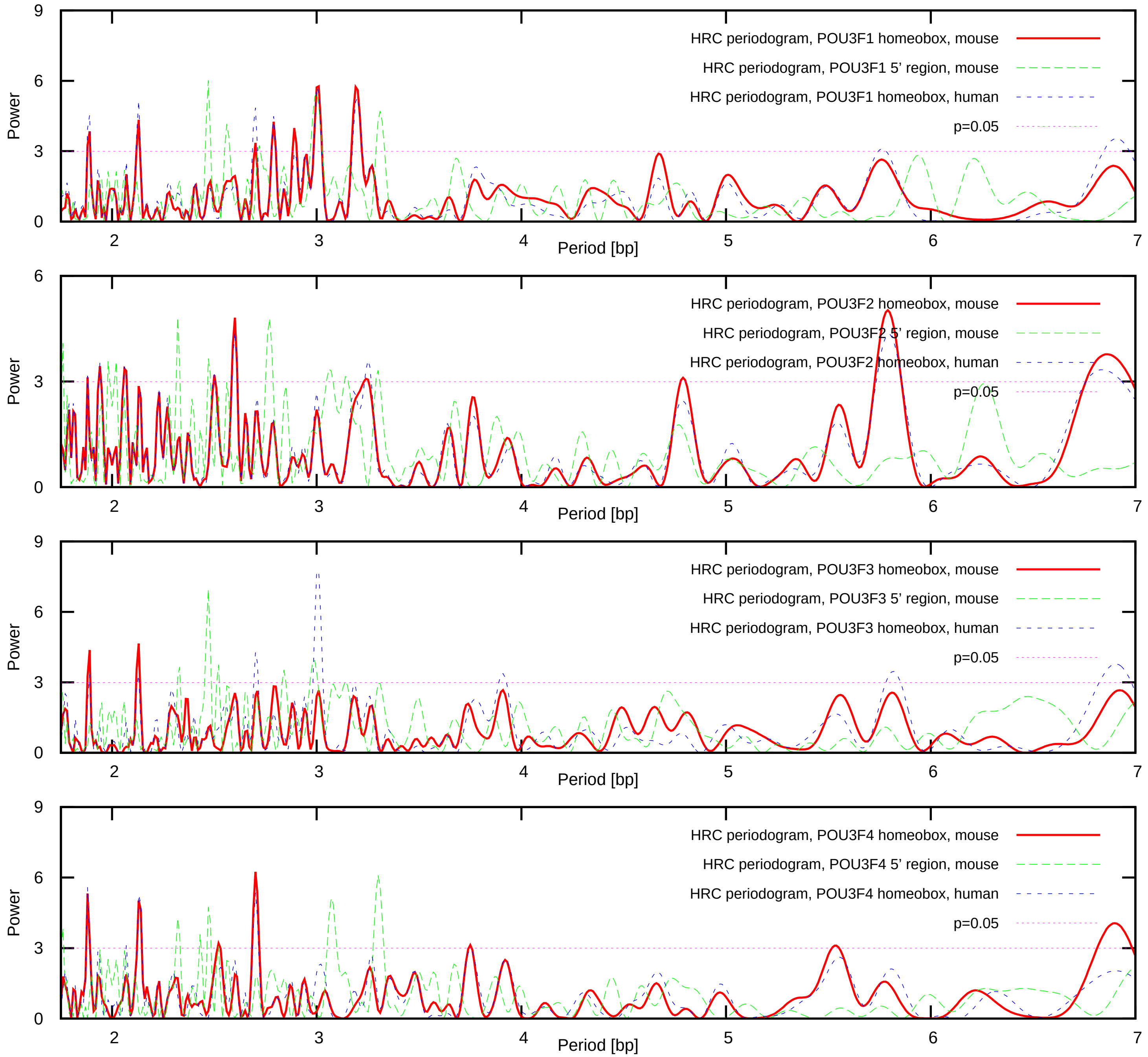

Power
Period [bp]
HRC periodogram, POU3F1 homeobox, mouse
HRC periodogram, POU3F1 5' region, mouse
HRC periodogram, POU3F1 homeobox, human
p=0.05
HRC periodogram, POU3F2 homeobox, mouse
HRC periodogram, POU3F2 5' region, mouse
HRC periodogram, POU3F2 homeobox, human
p=0.05
HRC periodogram, POU3F3 homeobox, mouse
HRC periodogram, POU3F3 5' region, mouse
HRC periodogram, POU3F3 homeobox, human
p=0.05
HRC periodogram, POU3F4 homeobox, mouse
HRC periodogram, POU3F4 5' region, mouse
HRC periodogram, POU3F4 homeobox, human
p=0.05

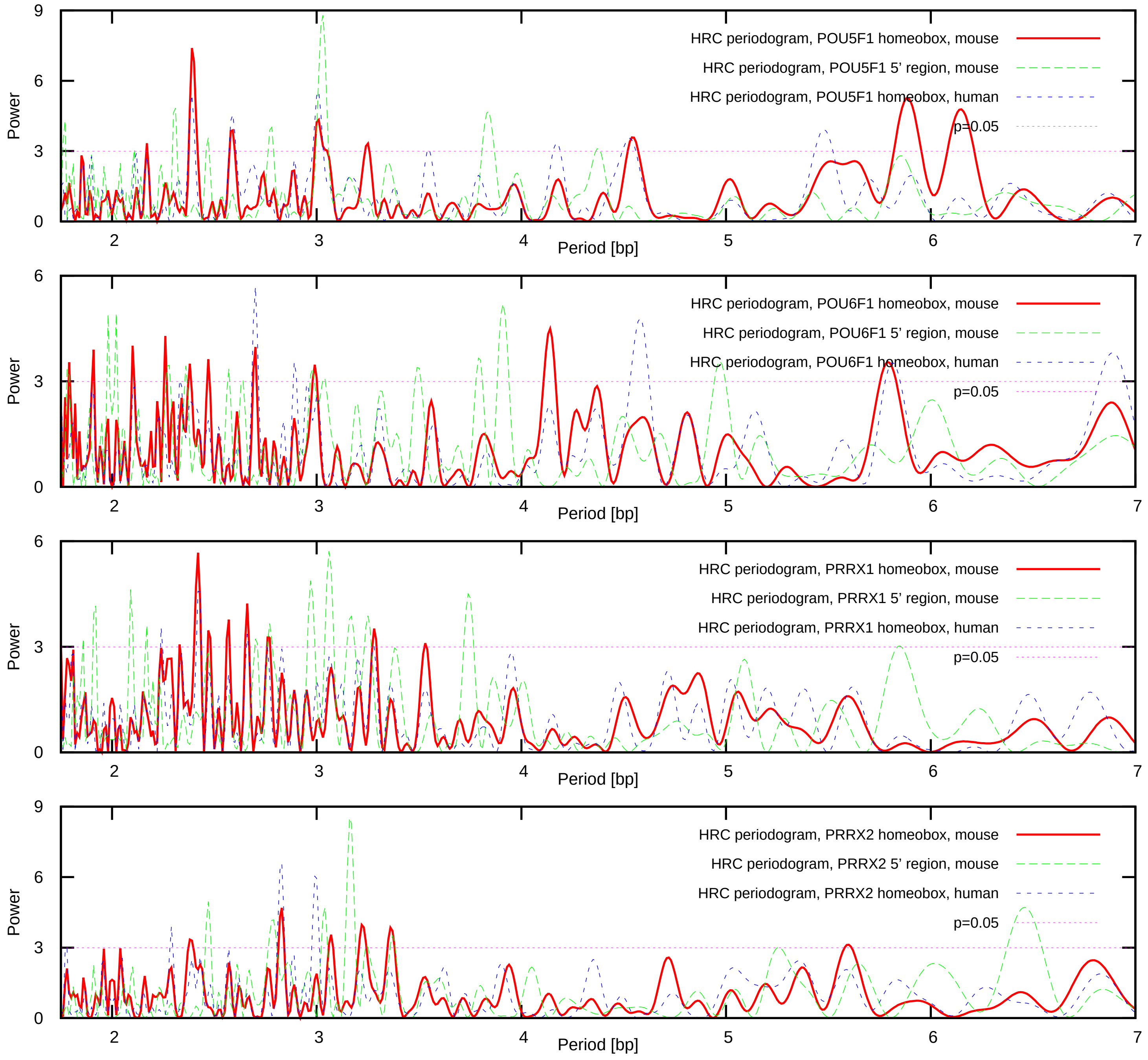

Power
Period [bp]
HRC periodogram, POU5F1 homeobox, mouse
HRC periodogram, POU5F1 5' region, mouse
HRC periodogram, POU5F1 homeobox, human
p=0.05
HRC periodogram, POU6F1 homeobox, mouse
HRC periodogram, POU6F1 5' region, mouse
HRC periodogram, POU6F1 homeobox, human
p=0.05
HRC periodogram, PRRX1 homeobox, mouse
HRC periodogram, PRRX1 5' region, mouse
HRC periodogram, PRRX1 homeobox, human
p=0.05
HRC periodogram, PRRX2 homeobox, mouse
HRC periodogram, PRRX2 5' region, mouse
HRC periodogram, PRRX2 homeobox, human
p=0.05

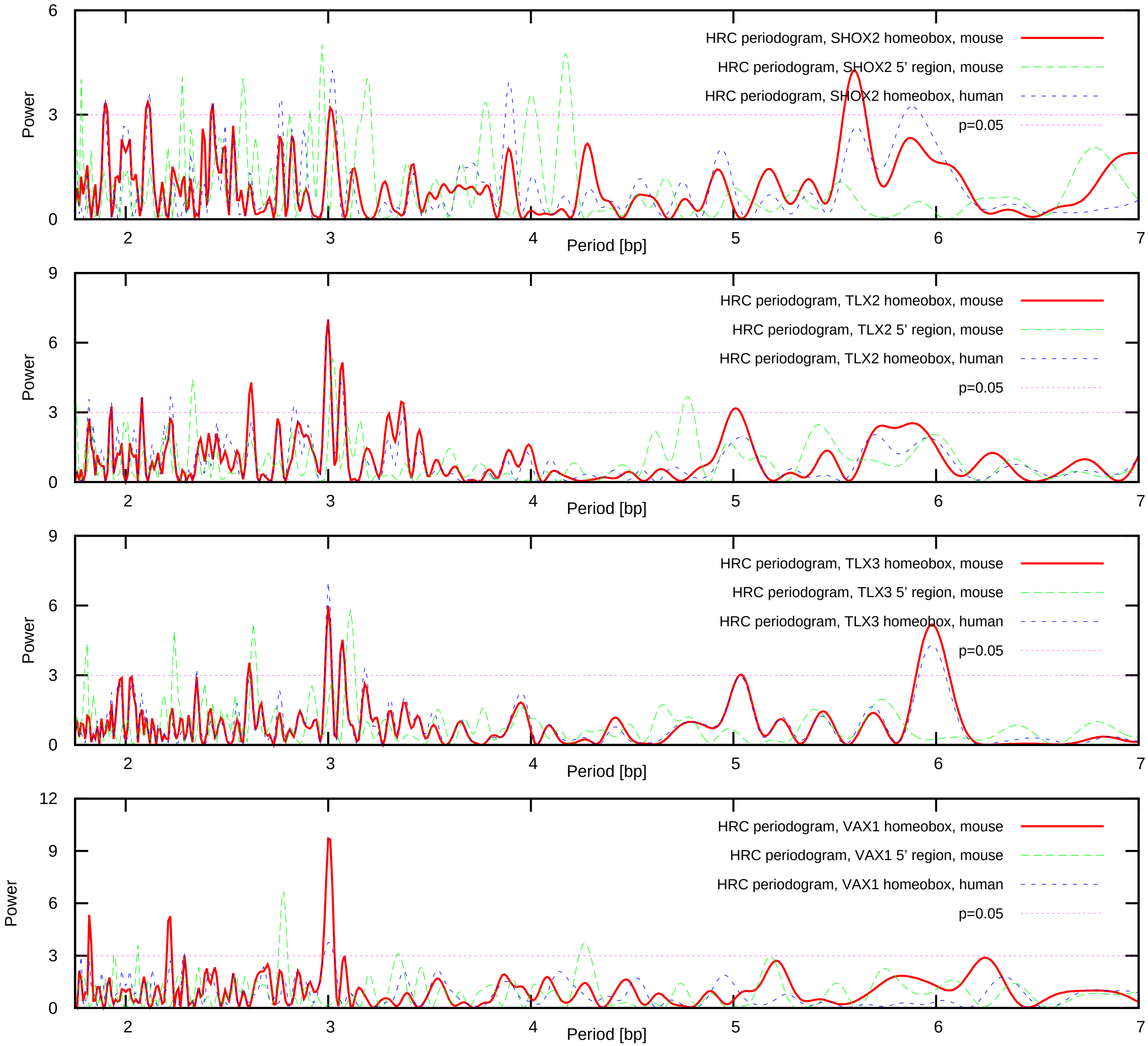

HRC periodogram, SHOX2 homeobox, mouse
HRC periodogram, SHOX2 5' region, mouse
HRC periodogram, SHOX2 homeobox, human
p=0.05
Power
Period [bp]
HRC periodogram, TLX2 homeobox, mouse
HRC periodogram, TLX2 5' region, mouse
HRC periodogram, TLX2 homeobox, human
p=0.05
Power
Period [bp]
HRC periodogram, TLX3 homeobox, mouse
HRC periodogram, TLX3 5' region, mouse
HRC periodogram, TLX3 homeobox, human
p=0.05
Power
Period [bp]
HRC periodogram, VAX1 homeobox, mouse
HRC periodogram, VAX1 5' region, mouse
HRC periodogram, VAX1 homeobox, human
p=0.05
Power
Period [bp]

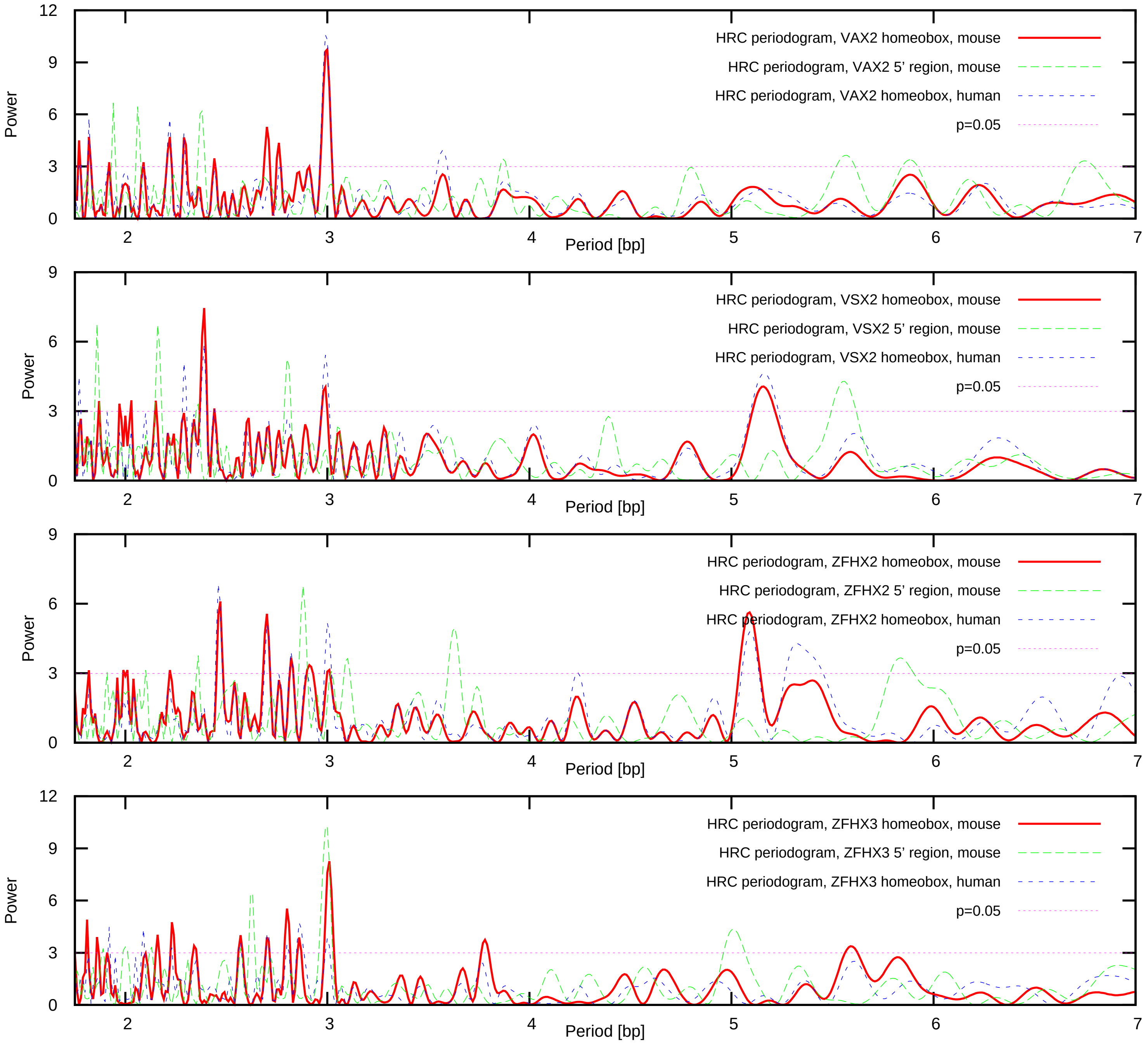
HRC periodogram, VAX2 homeobox, mouse
HRC periodogram, VAX2 5' region, mouse
HRC periodogram, VAX2 homeobox, human
p=0.05
HRC periodogram, VSX2 homeobox, mouse
HRC periodogram, VSX2 5' region, mouse
HRC periodogram, VSX2 homeobox, human
p=0.05
HRC periodogram, ZFHX2 homeobox, mouse
HRC periodogram, ZFHX2 5' region, mouse
HRC periodogram, ZFHX2 homeobox, human
p=0.05
HRC periodogram, ZFHX3 homeobox, mouse
HRC periodogram, ZFHX3 5' region, mouse
HRC periodogram, ZFHX3 homeobox, human
p=0.05
Power
Period [bp]

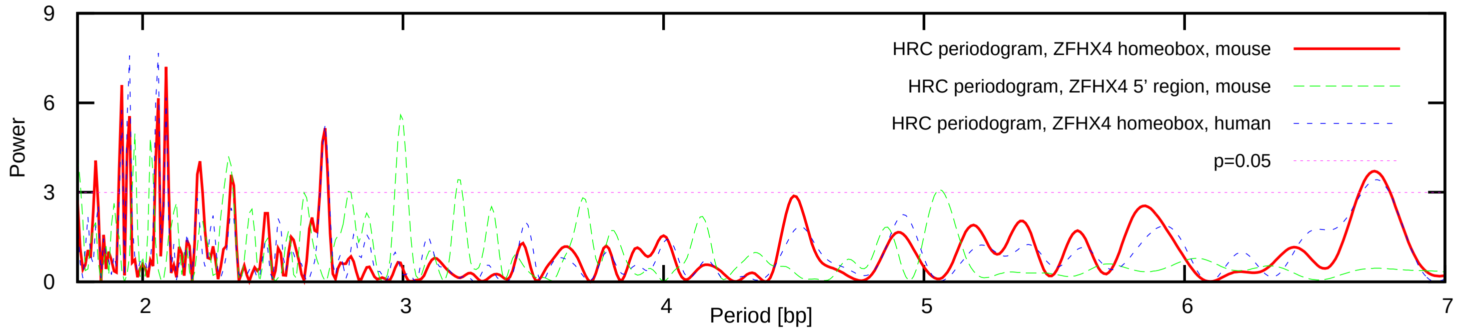

Power
Period [bp]
0
3
6
9
2
3
4
5
6
7
HRC periodogram, ZFHX4 homeobox, mouse
HRC periodogram, ZFHX4 5' region, mouse
HRC periodogram, ZFHX4 homeobox, human
p=0.05

Supplementary Program File SP1. The computer program (perl code) for calculating the HRC3 signature for actual and simulated homeobox coding sequences. The code contains two input files (tetramer_table.txt and codon_usage_mouse.txt).

```
#!/usr/bin/perl

# this program identifies the homeobox within DNA sequence and computes the HRC3
 signature.
# the result is compared against simulated sequences coding for the same protein
 sequence.

# make sure the two variables below point to CCDS annotation and CCDS fasta file
s in your system:
my $ccds_annot_file = "/bioinfo/andrzej/Projects/Homeobox-GC/CCDS-Mouse-042016/CCDS.current.txt"
;
my $ccds_fasta_file = "/bioinfo/andrzej/Projects/Homeobox-GC/CCDS-Mouse/CCDS_nucleotide.current.f
na";

use LWP::Simple;
use Bio::SeqIO;
use Bio::Seq;
use Bio::Tools::Run::StandAloneBlastPlus ;
use Bio::AlignIO ;
use Bio::AlignIO::bl2seq ;

my $simulate = 1;
#my $nsims = 100;
my $nsims = 1000;

my $tlen = 180;
my $omega = 2. * 3.1415926536 / 3.;
my @somt, @comt;
for($ii=0; $ii < $tlen; ++$ii)
{ $somt[$ii] = sin($omega * $ii); $comt[$ii] = cos($omega * $ii); }

my $homeobox = Bio::Seq->new( -seq => 'RRRKRTAYTRYQLLELEKEFLFNRYLTRRRRIELAHSL
NLTERHIKIWFQNRRMKWKKEN', -id  => 'HOMEOBOX', -accession_number => 'HBX',);

@amino_acids = qw/* A C D E F G H I K L M N P Q R S T V W Y/;
for (@amino_acids) { $cd{$_} = 0.; };
open CU, "codon_usage_mouse";
while (<CU>)
{
        /^#/ and next; chomp;
        s/\(/ /g; s/\)/ /g;
        @a = split;
        $aa{$a[0]} = $a[1];
        $cd_min{$a[1]}{$a[0]} = $cd{$a[1]};
        $cd{$a[1]} += $a[2];
        $cd{$a[1]} = 1 if ( $cd{$a[1]} > 0.98 );
        $cd_max{$a[1]}{$a[0]} = $cd{$a[1]};
};
close CU;

open BE, "tetramer_table.txt";
while (<BE>) { /^#/ and next; chomp; my ($ssq, $t1, $t2, $t3, $t4) = split; @{$b
end_tetramer{$ssq}}=($t1, $t2, $t3, $t4);};
close BE;
my %bend_7;
my @acgt = qw/A C G T/;
for $b1 (@acgt) { for $b2 (@acgt) { for $b3 (@acgt) { for $b4 (@acgt) { for $b5
(@acgt) { for $b6 (@acgt) { for $b7 (@acgt) {
        my $hept = $b1.$b2.$b3.$b4.$b5.$b6.$b7;
        my $tetr1 = $b1.$b2.$b3.$b4; my $tetr2 = $b2.$b3.$b4.$b5;
        my $tetr3 = $b3.$b4.$b5.$b6; my $tetr4 = $b4.$b5.$b6.$b7;
        $bend_7{$hept} = 0.33333333 * (
                                0.5 * $bend_tetramer{$tetr1}[3] +
```

```
                                1.0 * $bend_tetramer{$tetr2}[2] +
                                1.0 * $bend_tetramer{$tetr3}[1] +
                                0.5 * $bend_tetramer{$tetr4}[0] );
        $bend_7{$hept} = sprintf "%4f", $bend_7{$hept};
};};};};};};};

print STDERR "reading CCDS annotation file\n";
open CA, $ccds_annot_file;
while (<CA>)
{
        next if /^#/; chomp; @a = split;
        $ccds_id = $a[4]; $gene = $a[2];
        $gene{$ccds_id} = $gene;
}
close CA;

print STDERR "reading CCDS fasta file and simulating\n";
my $seqio = Bio::SeqIO->new(-file => $ccds_fasta_file, '-format' => 'Fasta');
while(my $seq = $seqio->next_seq) {
          my $string = $seq->seq;
          $ccds_id = [split /\|/,$seq->display_id]->[0];
          $gene = $gene{$ccds_id};
          $id =  $seq->display_id;
          $gene{$id} = $gene;
          next unless ($gene =~ /Hox/) ;
          print STDERR $ccds_id, "\t", $gene ,"\n";
          $dna{$id} = $string;
          # simulate
          for $n (0..(length $string)/3-1)
          {
                $ss = substr $string, 3*$n, 3;
                $aa = $aa{$ss};
                for $ns (1..$nsims)
                {
                        # this is a first order analysis, with short data series
, therefore
                        # use of the built-in rand() function is justified.
                        $rr = rand;
                        for $k (keys %{$cd_min{$aa}})
                        {
                                $sim_ss = $k if ( ($rr > $cd_min{$aa}{$k}) and (
 $rr < $cd_max{$aa}{$k} ) );
                        };
                        $sim_dna{$id}{$ns} .= $sim_ss;
                };
          };
        for $ns (1..$nsims)
        {
          # print STDERR "S $ns\t",$sim_dna{$ccds_id}{$ns},"\n";
        }
        # BLAST
        my $ggene =  Bio::Seq->new( -seq => $seq->seq, -id  => $id, -accession_n
umber => $id,);
        $factory_n = Bio::Tools::Run::StandAloneBlastPlus->new(-db_name => 'testdb
', -create => 1);
        $factory_n->bl2seq(-method => 'tblastn', -query => $homeobox, -subject =>
$ggene);
        $res_file = $factory_n->blast_out;
        # print STDERR "Blast file $res_file\n";
        open BF, $res_file;$gotit = '';
        @{$ss1s{$id}} = ();  # homeobox subject starts
        @{$ss2s{$id}} = ();  # homeobox subject ends
        @{$qq1s{$id}} = ();  # homeobox query starts
```

```
        @{$qq2s{$id}} = ();  # homeobox query ends
        $sqi = -1;
        while (<BF>)
        {
                if ( /Score/ )
                {
                        ++$sqi;
                        #print STDERR "score $sqi \n";
                };
                if ( /Sbjct/ )
                {
                        ($start, $stop) = ( [split]->[1], [split]->[3]) ;
                        $ss1s{$id}[$sqi] = $start;
                        $ss2s{$id}[$sqi] = $stop;
                        $got_good_hbx{$id} = 1 if ( abs ( $start - $stop ) > 140
 );
                };
                if ( /Query *[0-9]/ )
                {
                        ($qstart, $qstop) = ( [split]->[1], [split]->[3]) ;
                        $qq1s{$id}[$sqi] = $qstart;
                        $qq2s{$id}[$sqi] = $qstop;
                };
        }
        close BF;
        $n_homeoboxes{$id} = $sqi + 1;
        $factory_n->cleanup;
};
print STDERR "done reading\n";

# LOOP OVER SEQs
foreach $id ( sort { $gene{$a} cmp $gene{$b} } keys %dna )
{
        # skip if no homeobox found
        next unless ( $n_homeoboxes{$id} > 0 );
        next unless ( $got_good_hbx{$id} );
        # make HRC sequence
        @{$b{$id}} = ();
        for (0..2) {push @{$b{$id}}, 0};
        $l = length $dna{$id};
        for ($ii=3; $ii<$l-3; ++$ii)
        {
                $ss = substr $dna{$id}, $ii-3, 7 ;
                push @{$b{$id}}, $bend_7{$ss};
        };
        for (0..2) {push @{$b{$id}}, 0};
        for $ns (1..$nsims)
        {
                @{$simb{$id}{$ns}} = ();
                for (0..2) {push @{$simb{$id}{$ns}}, 0};
                for ($ii=3; $ii<$l-3; ++$ii)
                {
                        $ss = substr $sim_dna{$id}{$ns}, $ii-3, 7 ;
                        push @{$simb{$id}{$ns}}, $bend_7{$ss};
                };
                for (0..2) {push @{$simb{$id}{$ns}}, 0};
        };
        #
        for $sqi (0..$n_homeoboxes{$id}-1)
        {
                $stop  =  $ss2s{$id}[$sqi];
                $start =  $ss1s{$id}[$sqi];
                $qstart = $qq1s{$id}[$sqi];
```

```
                $qstop  = $qq2s{$id}[$sqi];
                print STDERR $id, " ", $sqi, " ", $start, " ", $stop, " ", abs($st
op - $start)  , "\n";
                next if ( abs($stop - $start) < 140);
                $hbx_a = $start-($qstart-1)*3;
                $hbx_b = $start-($qstart-1)*3 + 180;
                $hbx_seq = substr $dna{$id}, $hbx_a-1, 180;
                $fiv_seq = substr $dna{$id}, 0, $hbx_a-1;
                $thr_seq = substr $dna{$id}, $hbx_a-1 + 180;
                $hbx_gcc = gcc ($hbx_seq);
                $fiv_gcc = gcc ($fiv_seq);
                $thr_gcc = gcc ($thr_seq);
                # test print homeobox // and translation
                $hbx_seq_obj = Bio::Seq->new(-seq => $hbx_seq, -display_id => $i
d."_hbx_".$sqi, -desc => $id."_hbx_".$sqi, -alphabet => "dna" );
                $hbx_tr8_obj = $hbx_seq_obj -> translate;
                $tseq = $hbx_tr8_obj -> seq;
                #print STDERR "Actual: ", $tseq , "\n";
                $j = $hbx_a-1;
                $j5 = $j-180; # 5' of HBX
                $j0 = 0;     # 5' end
                # jstarts -> different starts we consired here
                $jstarts{jh} = $j;  # at start of homeobox; others can be added
                $show_non_hbx = 0 ; # zero to show homeobox only; not 5' or 3' e
nds
                if ($show_non_hbx)
                {
                        $jstarts{j5} = $j5;
                        $jstarts{j0} = $j0;
                };
                # loop over all j starts
#               for $js ( keys %jstarts )
#               { print $js,":" , $jstarts{$js}, "\t"; };
#               print "\n";
                # loop over all j starts
                for $js ( keys %jstarts )
                {
# gcc here
                        for $ns (1..$nsims)
                        {
                                $sim_hbx_seq = substr $sim_dna{$id}{$ns} , $jsta
rts{$js}, 180;
                                $sim_hbx_gcc{$ns} = gcc ($sim_hbx_seq);
                                $dd = Bio::Seq->new(-seq => $sim_hbx_seq, -displ
ay_id => $id."_sim_hbx_".$sqi, -desc => $id."_sim_hbx_".$sqi, -alphabet => "dna" );
                                $dd_tr8 = $dd -> translate;
                                #print STDERR "Sim $ns : ", $dd_tr8->seq , "\n";
                        }
                        $scx =  0; $ssx =  0; $sum =  0; $ssq =  0;
# fourier here
                        for $ns (1..$nsims)
                        {
                                $scxs{$ns} =  0; $ssxs{$ns} =  0; $sums{$ns} =
0; $ssqs{$ns} =  0;
                        }
                        for ($k = 0; $k < $tlen ; ++$k)
                        {
        # real
                                $scx += $b{$id}[$jstarts{$js}+$k] * $comt[$k];
                                $ssx += $b{$id}[$jstarts{$js}+$k] * $somt[$k];
                                $ssq += $b{$id}[$jstarts{$js}+$k] * $b{$id}[$jst
arts{$js}+$k];
                                $sum += $b{$id}[$jstarts{$js}+$k];
```

```
		# simulated
					for $ns (1..$nsims)
					{
						$scxs{$ns} += $simb{$id}{$ns}[$jstarts{$
js}+$k] * $comt[$k];
						$ssxs{$ns} += $simb{$id}{$ns}[$jstarts{$
js}+$k] * $somt[$k];
						$ssqs{$ns} += $simb{$id}{$ns}[$jstarts{$
js}+$k] * $simb{$id}{$ns}[$jstarts{$js}+$k];
						$sums{$ns} += $simb{$id}{$ns}[$jstarts{$
js}+$k];
					};
				}
				$sigsq = $ssq / $tlen - $sum*$sum/($tlen*$tlen);
				$hrc3_f = ( $ssq > 0.0000001 ) ?  1./ ($sigsq * $tlen) *
 ( $scx*$scx  + $ssx*$ssx ) : 0 ;
# OUTPUT HERE
				print +join "\t", $id, $gene{$id}, $js, $sqi, "hrc3", spri
ntf ("%.3f",$hrc3_f);
				for $ns (1..$nsims)
				{
					$sigsqs{$ns} = $ssqs{$ns} / $tlen - $sums{$ns}*$
sums{$ns}/($tlen*$tlen);
					$hrc3_fs{$ns} = ( $ssqs{$ns} > 0.0000001 ) ?  1.
/ ($sigsqs{$ns} * $tlen) * ( $scxs{$ns}*$scxs{$ns}  + $ssxs{$ns}*$ssxs{$ns} ) :
0 ;
				};
				print "\tsim";
				$nsm = 0; $nlg = 0;
				for $ns (1..$nsims) { if ( $hrc3_fs{$ns} >  $hrc3_f ) {
$nsm++ ; } else { $nlg++ }; };
				print "\t$nsm/$nlg";
				print "\t",$nsm/$nsims;
				print "\n";
				$output_gcc = 0;
			}
		}
}

exit;

sub gcc
{
		my $gc = 0; my $at = 0; my $tl = 0;
		my @s = split //, $_[0];
		for (@s) { ++$tl; $_ =~ /[gcGC]/ and ++$gc; };
		return $gc / $tl;
};

sub corr {
		my ($aref, $bref) = @_;
		my $n;
		my @zna, @znb, @za, @zb;
		if ( $#$aref != $#$bref )
		{
			#print STDERR "ERROR in sub corr!   $#$aref != $#$bref \n";
			return "NA"
		};
		$n = $#$bref + 1;
		@za = @{$aref}; @zb = @{$bref};
		@zna = normalize_sigma (@za);
		@znb = normalize_sigma (@zb);
		my $ss = 0;
```

```
        for (0..$n-1)
        {
                $ss += $zna[$_] * $znb[$_];
        };
        $ss /= $n;
        return $ss;
};

sub normalize_sigma {
        my @num = @_;
        my $sum = 0;
        for (@num) { $sum += $_ ; };
        $sum /= (1.0 + $#num);
        for (0..$#num) { $num[$_] -= $sum ; } ;
        my $ssq = 0;
        for (@num) { $ssq += $_ * $_ ; };
        $ssq /= (1.0 + $#num);
        $ssq = sqrt($ssq);
        for (0..$#num) { $num[$_] /= $ssq ; } ;
        return @num;
};

sub stats {
        my ($aref) = @_;
        my $sum = 0.0;
        my $dssum = 0.0;
        for ($i=0; $i <= $#$aref; ++$i) { $sum += $$aref[$i]; };
        $avg = $sum / ($#$aref + 1.0);
        for ($i=1; $i <= $#$aref; ++$i) { $dssum += ($$aref[$i]-$avg)*($$aref[$i
]-$avg); };
        $sig = sqrt ($dssum / ($#$aref + 1.0));
        print ("STAT ", $avg, "\t", $sig, "\n");
        return ($avg, $sig);
};
```

```
# Fields: Triplet | Amino acid | Fraction | Frequency/Thousand | (Number)
#
TTT F 0.43 17.1(244935)
TCT S 0.19 15.9(228046)
TAT Y 0.43 12.2(175198)
TGT C 0.48 11.1(158573)
TTC F 0.57 22.3(319002)
TCC S 0.22 18.1(258787)
TAC Y 0.58 16.5(236579)
TGC C 0.52 12.1(173509)
TTA L 0.06 6.5( 92974)
TCA S 0.14 11.6(165371)
TAA * 0.26 0.6( 8861)
TGA * 0.52 1.2( 17004)
TTG L 0.13 13.3(189840)
TCG S 0.05 4.3( 61116)
TAG * 0.22 0.5( 7491)
TGG W 1.00 12.4(177261)
CTT L 0.13 13.2(188236)
CCT P 0.30 18.3(261478)
CAT H 0.40 10.2(145279)
CGT R 0.09 4.7( 66962)
CTC L 0.20 20.3(290198)
CCC P 0.31 18.4(262874)
CAC H 0.60 15.2(217261)
CGC R 0.18 9.5(136330)
CTA L 0.08 8.0(114707)
CCA P 0.28 17.1(244064)
CAA Q 0.25 11.4(163794)
CGA R 0.12 6.6( 94626)
CTG L 0.39 40.0(571592)
CCG P 0.10 6.3( 89477)
CAG Q 0.75 34.0(486041)
CGG R 0.19 10.3(147905)
ATT I 0.34 15.6(223528)
ACT T 0.25 13.6(194120)
AAT N 0.43 15.5(221760)
AGT S 0.15 12.3(176056)
ATC I 0.50 23.2(332072)
ACC T 0.35 19.2(274588)
AAC N 0.57 20.7(296083)
AGC S 0.24 19.5(279098)
ATA I 0.16 7.2(103441)
ACA T 0.29 15.9(227958)
AAA K 0.39 21.3(305128)
AGA R 0.21 11.4(163062)
ATG M 1.00 23.1(330431)
ACG T 0.11 5.8( 82653)
AAG K 0.61 33.7(482643)
AGG R 0.22 11.7(167166)
GTT V 0.17 10.7(152706)
GCT A 0.29 20.1(288132)
GAT D 0.44 21.1(301359)
GGT G 0.18 11.5(164971)
GTC V 0.25 15.7(224465)
GCC A 0.38 26.4(378142)
GAC D 0.56 26.6(380355)
GGC G 0.33 21.9(312846)
GTA V 0.12 7.4(105532)
GCA A 0.23 15.8(226309)
GAA E 0.40 26.5(379216)
GGA G 0.26 16.9(241435)
GTG V 0.46 29.1(416428)
```

```
GCG A 0.10 6.6( 94463)
GAG E 0.60 39.4(563999)
GGG G 0.23 15.4(220474)
```

GAGA	0.742	0.058	1.117	0.025
TACA	0.440	−0.185	1.078	−0.473
CTGG	0.417	−0.227	0.427	1.309
CTAC	−0.062	−0.377	−0.119	0.684
AGAT	−0.196	1.129	−0.001	0.489
GATA	0.730	0.151	0.801	−0.547
TCAG	0.565	0.392	−0.432	1.370
ATTT	−0.176	0.396	−0.544	−0.977
TAGC	0.176	−0.577	1.025	0.791
GACT	0.837	0.143	0.536	−0.371
CCAG	0.264	0.169	−0.407	1.777
ATCC	0.341	0.780	0.042	−0.147
TCGC	0.235	−0.120	0.712	1.229
GTCC	0.964	0.752	0.399	0.057
TACC	0.856	−0.115	0.814	0.093
GTCG	0.786	0.611	0.044	0.583
GCGA	1.053	1.233	0.823	0.802
TATT	0.599	−0.366	0.564	−0.110
ATCA	−0.302	0.475	0.122	−0.491
AGTA	−0.167	0.924	0.601	−0.349
GACA	0.258	0.116	0.979	−0.421
CAGA	0.700	−0.645	1.557	0.263
GGGT	0.163	0.792	0.930	0.179
TCGT	0.444	0.175	0.703	1.713
CGGT	0.574	0.594	1.912	1.119
CGTT	0.348	1.090	2.109	0.676
CGTG	0.516	0.696	1.746	0.821
AGGT	−0.185	1.115	1.282	0.355
CTTC	0.684	0.328	0.447	0.365
TCCT	0.675	0.185	−0.060	0.269
TGTG	0.656	0.724	0.941	0.871
TAAG	0.859	−0.216	0.106	0.717
TCGG	−0.028	−0.132	0.135	1.318
CCTG	0.456	0.027	−0.061	0.745
TTCG	0.129	−0.073	−0.273	0.490
AACT	−0.013	0.158	−0.093	−0.934
GTTG	1.156	1.354	−0.116	0.410
CCCT	0.387	−0.194	0.088	0.309
TCGA	0.594	0.126	0.779	0.637
ACCG	0.048	0.705	0.008	0.582
TGAA	0.162	0.563	0.060	−0.045
CGAA	0.336	0.542	0.543	0.250
ATAC	−0.212	0.916	−0.520	0.516
TCCG	0.363	0.257	0.050	0.846
CTGT	0.651	0.005	1.041	1.263
TAAA	0.092	−0.358	−0.068	−0.318
GGCT	0.557	1.023	1.143	0.088
GTCA	1.375	1.015	0.687	−0.061
GTAA	0.681	0.675	−0.313	0.222
TAGA	0.550	−0.413	0.980	0.109
ATGC	−0.152	0.721	0.316	0.809
GCTG	0.912	1.295	−0.144	0.861
TAAC	0.179	−0.393	0.204	0.561
GGTG	0.616	1.576	0.739	0.444
TTGG	0.354	−0.187	0.064	1.082
TGGT	0.264	0.162	1.410	0.602
GGGA	0.676	0.727	0.800	−0.024
GGCG	0.153	1.085	1.021	0.476
TATC	0.504	−0.348	0.843	0.158
GGCC	0.567	0.840	0.634	−0.352
ACGA	−0.226	0.633	0.521	0.440
ACGG	−0.383	0.811	0.518	1.086
GAAA	0.649	0.204	0.046	−0.685

TGTT	0.180	0.829	1.267	0.016
TACT	0.258	−0.359	0.523	0.042
TCTT	0.072	−0.268	0.052	0.462
TTGA	−0.517	−0.773	0.365	−0.035
GCCT	0.810	0.992	−0.014	0.118
TTAC	−0.320	−0.328	−0.416	0.644
GGAA	0.566	1.044	0.155	−0.081
TCTC	0.370	−0.005	−0.108	0.426
ATAT	−0.197	0.882	−0.515	0.306
CGCC	0.613	0.412	0.977	−0.036
ATTC	0.291	0.836	−0.054	−0.441
GTAG	0.871	1.005	−0.287	1.119
CAAA	0.061	−0.418	0.359	−0.018
ACAC	0.083	1.133	−0.362	1.315
GTAT	0.716	0.944	−0.262	0.941
TATA	0.813	−0.060	0.974	−0.461
GATC	1.031	0.298	0.510	−0.111
ATCT	−0.016	0.633	−0.287	−0.416
CGAG	0.630	0.731	0.494	1.177
TAGT	0.496	−0.360	1.113	0.785
GGGC	0.344	0.778	0.741	0.798
TCCC	−0.043	−0.104	−0.332	0.089
CTCA	0.140	−0.287	0.485	−0.214
GTAC	0.771	0.960	−0.169	1.140
AAGG	−0.385	−0.239	0.275	0.389
CGGA	0.161	0.262	1.028	0.252
ACAA	−0.520	0.673	−0.666	0.192
GAGG	0.575	0.132	0.757	1.090
GGTC	0.970	1.429	0.417	0.146
GTTT	0.987	1.166	−0.092	−0.289
CCCG	0.434	−0.037	0.083	0.896
CCAA	−0.293	−0.114	−0.141	0.693
GAGC	0.936	−0.005	0.902	0.400
TCTG	0.213	0.119	−0.136	1.023
CCCA	−0.238	−0.330	0.482	0.125
ACGT	−0.195	0.603	0.731	1.349
AGCA	−0.170	1.123	0.635	−0.572
CTTA	0.275	−0.110	0.456	−0.192
GGGG	0.056	0.722	0.646	0.656
AAGC	0.140	0.246	0.619	0.288
AGCC	0.127	1.337	0.416	0.010
CCGA	0.632	0.078	0.863	0.621
TTTC	0.611	−0.252	−0.366	−0.582
CCGT	0.510	0.089	1.117	2.249
CATA	0.223	−0.703	1.251	−0.218
CGCT	0.192	0.354	1.498	0.031
GTTA	0.950	0.701	0.067	−0.413
AGTG	−0.458	0.903	0.525	0.501
CTCG	0.437	0.179	0.506	0.889
AATT	−0.088	−0.018	−0.044	−0.867
CGAC	0.518	0.694	0.582	0.972
AGCG	−0.243	0.949	0.747	0.492
TTCA	0.825	0.295	0.172	−0.372
TGCA	0.384	0.499	1.396	−0.129
CCCC	0.271	−0.268	−0.117	0.076
GGAG	0.468	0.797	−0.106	0.803
GTGA	0.932	0.835	0.896	0.508
AGGC	−0.518	0.600	0.699	0.802
TCTA	0.574	0.103	−0.124	−0.225
ATTA	−0.131	0.344	−0.235	−0.696
CAGG	0.512	−0.764	1.329	0.987
AGAA	−0.382	1.096	0.020	−0.200
AATC	0.009	0.025	−0.170	−0.693

GTGC	1.363	1.291	1.053	1.528
CTCT	0.311	−0.062	0.475	0.330
GCAA	0.827	1.265	−0.138	0.342
AGAG	−0.120	0.893	−0.012	0.906
AATA	−0.569	−0.519	0.010	−0.960
ACTA	−0.278	0.263	−0.410	−0.264
ATGT	−0.022	1.006	0.600	0.764
AGGG	−0.383	0.655	0.690	0.597
AGAC	−0.278	1.094	−0.016	0.639
TGGG	0.143	−0.148	0.748	0.921
GGCA	0.700	1.070	1.013	−0.534
CATC	0.958	−0.075	1.232	0.295
CCTA	0.169	0.001	0.594	0.281
TCAA	0.691	0.734	−0.002	0.621
CACC	0.623	−0.286	0.946	−0.020
CTGC	0.884	0.030	1.126	1.585
GCTT	0.762	1.077	0.086	0.421
CTAT	0.458	0.154	0.020	1.346
CTTT	0.125	0.009	0.421	0.171
TAGG	0.211	−0.805	0.573	0.922
ATCG	−0.123	0.527	−0.228	0.368
CGGC	0.691	0.229	1.414	1.128
GTCT	1.089	0.866	0.285	0.222
AACA	−0.243	−0.106	0.371	−0.904
CACT	0.771	−0.177	1.079	0.083
GCAC	0.436	0.669	−0.679	0.998
TGAG	0.214	0.563	0.206	0.950
AACG	−0.267	0.134	0.506	0.114
GCCC	0.818	0.534	−0.193	0.118
TCAT	0.709	0.502	−0.240	1.468
GCGG	0.573	0.867	−0.117	1.493
TAAT	0.205	−0.358	−0.030	0.399
AAAT	−0.068	−0.127	−0.767	−0.884
AGGA	−0.709	0.400	0.613	−0.250
TGCT	0.782	1.002	1.453	0.225
TGGA	0.204	0.174	0.970	0.117
CGCG	0.315	0.601	1.447	0.487
GCCG	0.978	0.742	−0.170	0.913
GAAC	0.588	0.244	0.070	0.302
AAAA	−0.215	−0.095	−0.536	−1.126
CCTC	0.386	−0.202	0.107	0.597
ATAG	−0.158	0.713	−0.588	0.899
CAGT	0.686	−0.330	1.859	1.245
TTTA	0.073	−0.342	−0.426	−0.849
GGAT	0.294	0.996	0.038	0.386
AAGA	−0.058	−0.108	0.639	−0.397
TTGT	0.777	0.070	0.596	0.996
CCGC	0.326	−0.110	0.672	1.462
GAAT	1.064	0.264	−0.068	0.098
CCAT	0.573	0.188	−0.236	1.755
TGCG	0.204	0.898	1.363	0.627
TGTA	0.574	0.633	1.065	−0.258
CTCC	0.437	−0.394	0.207	−0.062
GTTC	1.314	1.254	0.247	0.071
AGCT	−0.308	0.712	0.588	−0.215
AAAG	−0.021	0.070	−0.324	0.162
CATG	0.768	−0.155	1.373	0.724
CACA	0.137	−0.511	1.400	−0.134
TTAG	0.276	−0.137	−0.800	0.627
CAAG	0.829	−0.411	0.260	0.734
AATG	0.057	−0.281	0.110	−0.024
CGGG	0.034	0.037	1.027	1.054
TGCC	0.598	0.713	1.182	−0.118

GCTA	0.635	1.162	0.134	0.083
TCAC	−0.466	−0.161	−0.465	1.009
CTTG	0.299	−0.171	0.108	0.620
TTAA	0.305	0.056	−0.514	−0.013
TGAT	−0.196	0.826	0.443	0.774
GACG	0.468	0.131	0.622	0.459
CGTC	0.841	0.781	1.411	0.536
ACTT	−0.007	0.558	−0.175	0.103
GCCA	0.664	0.773	−0.096	−0.407
CACG	0.383	−0.560	1.152	0.552
TTTG	0.507	−0.105	−0.404	0.315
GAGT	0.675	0.329	1.072	0.690
GCAG	0.938	1.318	−0.477	1.721
TTTT	0.111	−0.212	−0.648	−0.809
GTGT	0.747	0.957	0.650	1.232
GATT	1.138	0.557	0.520	−0.381
TTCC	0.967	0.222	−0.058	−0.024
CGCA	0.320	0.542	1.351	−0.392
AAAC	−0.379	−0.158	−0.668	−0.445
TTGC	0.504	−0.073	0.617	1.471
ACAT	−0.096	1.117	−0.224	0.937
CGAT	0.586	1.019	0.881	0.829
ACCA	−0.305	0.457	−0.008	−0.310
ACTC	0.302	0.423	−0.508	0.071
ATAA	−0.563	0.526	−0.562	−0.314
AACC	−0.155	−0.088	0.077	−0.576
ACTG	−0.226	0.652	−0.200	0.687
ATGG	−0.378	0.559	0.067	0.815
GGAC	0.222	0.574	−0.001	0.519
TGTC	0.627	0.701	0.983	0.391
GAAG	1.134	0.272	0.051	0.535
CAAT	0.714	−0.113	0.425	0.659
CCGG	0.200	−0.086	0.585	1.485
TGGC	0.402	0.412	1.210	1.168
AGTT	0.081	1.358	0.801	−0.313
CATT	0.916	−0.218	1.329	0.308
ATGA	−0.263	0.177	0.527	−0.006
ACCT	0.072	0.681	−0.216	−0.059
CCAC	0.608	0.273	−0.027	1.255
CGTA	0.526	0.415	1.553	0.101
TGAC	−0.288	0.558	0.207	0.683
ACGC	−0.040	0.820	0.337	1.208
GACC	1.266	0.382	0.679	0.125
ATTG	−0.343	0.326	−0.529	0.181
GATG	1.071	0.355	0.647	0.362
GTGG	0.856	0.868	0.041	1.133
GCAT	1.034	1.143	−0.331	1.121
GCTC	0.781	1.149	0.052	0.591
CTAA	0.125	−0.013	0.008	0.273
TTAT	0.162	0.094	−0.531	0.417
AAGT	0.017	0.196	0.615	−0.194
TTCT	0.163	−0.215	−0.557	−0.383
CTGA	0.141	−0.348	0.721	0.449
TACG	−0.382	−0.566	0.570	0.835
CTAG	0.557	0.037	−0.229	1.220
GGTA	0.510	1.068	0.365	−0.524
TCCA	0.321	0.250	0.138	−0.220
GCGT	1.129	1.431	0.740	1.809
CCTT	0.668	0.016	0.093	0.445
GGTT	0.518	1.365	0.653	−0.208
AGTC	0.365	1.453	0.625	0.327
ACCC	0.051	0.625	−0.144	0.235
CAGC	0.885	−0.257	1.576	0.908

GCGC	0.624	0.900	0.442	1.522
ACAG	−0.152	0.904	−0.679	1.452
TATG	0.158	−0.514	0.629	0.610
CAAC	0.862	−0.169	0.678	0.598
